# Generation of [(N4Py)Fe(IV)=O]^2+^ through Heterolytic O–O Bond Cleavage in [(N4Py)Fe(II)(OOH)]^+^

**DOI:** 10.1021/acs.inorgchem.4c05172

**Published:** 2025-05-02

**Authors:** Juan Chen, Andy S. Sardjan, C. Maurits de Roo, Marika Di Berto Mancini, Apparao Draksharapu, Davide Angelone, Ronald Hage, Marcel Swart, Wesley R. Browne

**Affiliations:** † Department of Applied Chemistry, School of Science, Northwestern Polytechnical University, Xi’an, Shaanxi 710072, China; ‡ Molecular Inorganic Chemistry, Stratingh Institute for Chemistry, Faculty of Science and Engineering, University of Groningen, Nijenborgh 3, 9747 AG Groningen, The Netherlands; ¶ ICREA, Pg. Lluis Companys 23, 08010 Barcelona, Spain; § Institut de Quimica Computacional i Catalisi (IQCC), Departament de Quimica, Universitat de Girona, Campus Montilivi, 17003 Girona, Catalonia, Spain

## Abstract

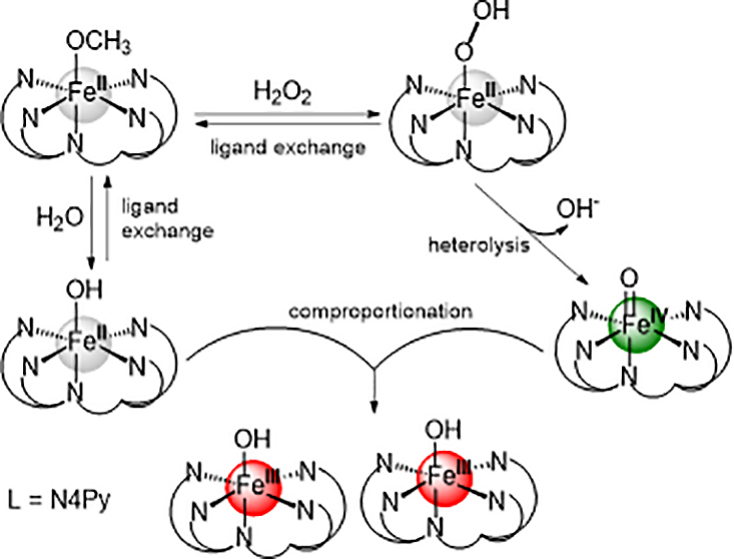

High-valent Fe(IV) oxido species are important intermediates in the catalyzed oxidation of organic compounds by nonheme iron enzymes. These species can be generated in biomimetic model complexes directly using oxygen atom transfer oxidants, e.g., PhIO and ClO^–^. Their formation by heterolysis of the O–O bond of putative Fe(II)-OOH species (formed from Fe(II) precursors and H_2_O_2_) has scarcely been observed. Reaction with near-stoichiometric H_2_O_2_ typically shows initial formation of Fe(III)-OH and Fe(III)-OOH species, with homolytic O–O bond cleavage thereafter proposed to generate the Fe(IV)=O state. Here, we show that [(N4Py)Fe(IV)=O]^2+^ (where N4Py = 1,1-di(pyridin-2-yl)-*N,N*-bis(pyridin-2-ylmethyl)methanamine) is formed with substoichiometric H_2_O_2_ in methanol through heterolytic cleavage of the O–O bond of an Fe(II)-OOH intermediate. Temperature-dependent studies show that the ligand exchange reactions preceding formation of the Fe(II)-OOH species and subsequent comproportionations limit the yield of the Fe(IV)=O species. Furthermore, comproportionation proceeds through hydrogen atom transfer from [(N4Py)Fe(II)(OH_2_)]^2+^ to [(N4Py)Fe(IV)=O]^2+^. These data rationalize the extent of the initial conversion of [(N4Py)Fe(II)(CH_3_CN)]^2+^ to [(N4Py)Fe(IV)=O]^2+^ under conditions relevant to catalytic oxidations. The heterolytic pathway to formation of [(N4Py)Fe(IV)=O]^2+^ is a key step in the development of iron(II) oxidation catalysts that can cycle between the Fe(II) and Fe(IV)=O states, avoiding nonselective reactive oxygen species.

## Abstract 2

Reaction of stoichiometric H_2_O_2_ with a nonheme Fe(II) complex shows heterolytic O–O bond cleavage to form a reactive Fe(IV)=O complex with comproportionation blocked by solvent coordination.

## Introduction

**Figure 1:**
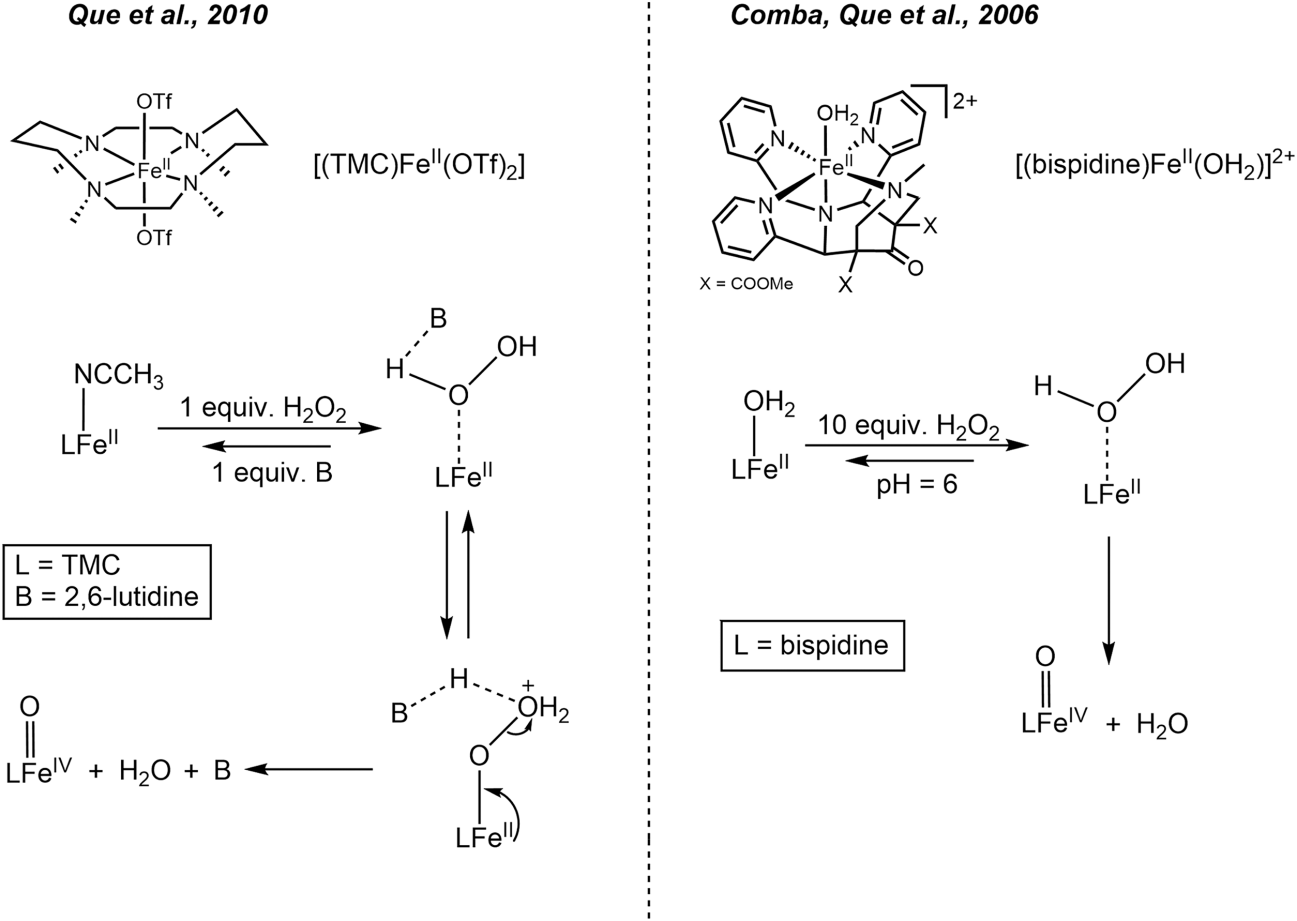
Fe(II) complexes discussed and the formation of Fe(IV)=O species through O–O bond heterolysis reported earlier.

High-valent Fe(IV)=O species are invoked frequently as the reactive species in the oxidation of organic substrates by heme and nonheme enzymes.^1−3^ In nature, enzymes use O_2_ and electron donors to generate these species from the Fe(II) redox state, e.g., through electron transfer chains or oxidative decarboxylation. Biomimetic nonheme Fe(IV)=O species have been generated from the corresponding Fe^II^ complexes of tetradentate N4 (TMC (tetramethylcyclam), BPMCN (*N*,*N*-bis(2-pyridylmethyl)-*N*,*N*-dimethyl-trans-1,2-diaminocyclohexane), etc.) and pentadentate N5 (N4Py, Bn-TPEN (N-benzyl-*N*,*N′N′*-tris(2-pyridylmethyl)-1,2-diaminoethane), bispidine, etc.) ligands,^4^ using two-electron oxidants, such as *m*-CPBA and peracetic acid,^5,6^ PhIO,^7−9^ HOCl,^10^ and hydroperoxides (e.g., *tert*-butyl-hydroperoxide and H_2_O_2_).^7,11−16^ In these catalysts, typically rapid net oxidation to an Fe^III^ (resting) state is observed with H_2_O_2_, and the relatively stable Fe^III^ (hydro)peroxy species is the starting point in catalytic cycles, not least because an Fe(III)-OOH species is observed in many cases.^14,17,18^ Indeed, it is the last observed intermediate in the cleavage of DNA with O_2_ by the antibiotic, iron bleomycin.^19^ O–O bond homolysis of the Fe(III)-OOH species to yield an Fe(IV)=O species and a hydroxyl radical is generally assumed, although, more recently O–O bond heterolysis to form a Fe(V)=O species has been proposed.^20−26^ The initial reaction between the Fe(II) complexes and H_2_O_2_, however, can be slow in solvents such as acetonitrile. Recently, McKenzie and Que groups have shown that including carboxylato motifs in the ligand makes the Fe(III) oxidation state the most stable form in solvents such as acetonitrile, which facilitates their reactions with oxidants.^27−31^ Catalytic pathways that involve only an Fe^II^/Fe^IV^ redox cycle are involved less often, despite the finding that the rebound mechanism,^32^ invoked often in C–H oxidation by Fe(IV)=O species, recovers the Fe(II) oxidation state. Chemical reduction of Fe(III)-OOH complexes and stoichiometric reactions between nonheme Fe^II^ complexes and H_2_O_2_ have received some attention, most notably by Nam,^33,34^ and by Que, Hirao, Comba, Banse, and co-workers, respectively.^35−39^ Under basic conditions at −40 °C, the N4-coordinated [(TMC)Fe^II^]^2+^ complex (Figure 1) provides the corresponding Fe(IV)=O species directly in over 90% yield with sub- and near-stoichiometric H_2_O_2_.^35^ Intramolecular base-promoted heterolysis was observed during the reaction of [(^NH^Bn-TPEN)Fe^II^]^2+^ with H_2_O_2_ in which a pendant base (an alkylamine) was introduced into the second coordination sphere to facilitate a proton-transfer-driven heterolytic O–O bond cleavage step.^35,36,38^ The quantitative generation of an Fe(IV)=O species from Fe^II^ complexes is not reported in other N4 and N5 systems; however, for the N5-coordinated Fe^II^(bispidine) complex (Figure 1), an Fe(IV)=O species was formed in water upon reaction with H_2_O_2_ in up to 60% yield.^36^ The lack of quantitative conversion in the latter case may be due to competing reactions, in particular, comproportionation between the Fe^II^ precursor and the Fe(IV)=O species generated, or reaction of H_2_O_2_ with the Fe(IV)=O species formed.^40^ Nevertheless, these studies show that the generation of Fe(IV)=O species in a Fe^II^/Fe^IV^ cycle is feasible.

Conditions in which Fe(IV)=O species form by O–O bond heterolysis in Fe(II)-OOH species is of fundamental interest since this pathway avoids the hydroxyl radicals that accompany O–O bond homolysis in Fe(III)-OOH species.^32,41^

The complex [(N4Py)Fe(II)(NCCH_3_)](ClO_4_)_2_ (**1**), bearing an N5 ligand, reacts with excess H_2_O_2_ in acetonitrile, forming a relatively stable Fe(III)-OOH intermediate, which was proposed to generate an Fe(IV)=O species upon homolytic cleavage of the O–O bond.^17,18,42^ Although the Fe(IV)=O species ([(N4Py)Fe(IV)=O]^2+^, **4**) is also relatively stable (can be isolated),^4,8^ its formation from the Fe(III)-OOH species is not readily observed, and its absence under reaction conditions with H_2_O_2_ is expected considering that it reacts rapidly with H_2_O_2_ to form Fe(III)-OH and O_2_^•–^.^43^ However, our recent studies in methanol show that the rate of cleavage of the O–O bond of Fe(III)-OOH is unexpectedly low and does not compete with H_2_O_2_ disproportionation by Fe(III)-OOH, nor with the reaction between two molecules of Fe(III)-OOH.^44^

Heterolytic O–O bond cleavage in an initially formed Fe(II)-OOH species would yield the same Fe(IV)=O species; however, the rapid reaction between Fe(IV)=O and H_2_O_2_, as well as the comproportionation reaction between Fe^II^ and Fe(IV)=O species can be expected to preclude the observation of **4**. In acetonitrile, **1** shows little if any reaction with stoichiometric H_2_O_2_, and only slow oxidation to the Fe(III) state is observed with excess (>50 equiv) H_2_O_2_.^40,43^

The low reaction rate is due to the kinetic and thermodynamic inertness of the low-spin CH_3_CN-bound Fe(II) complex. Exchange of the CH_3_CN ligand, ultimately with H_2_O_2_, is required for oxidation of the complex to take place; however, ligand exchange is unfavorable, even with excess H_2_O_2_. Hence, the initial reaction of H_2_O_2_ with the N5 Fe(II) complexes to form a putative Fe(II)-OOH precursor or Fe(IV)=O species is too slow, compared to subsequent reactions, to allow for a buildup in the concentration of either species in CH_3_CN.

**Scheme 1:**
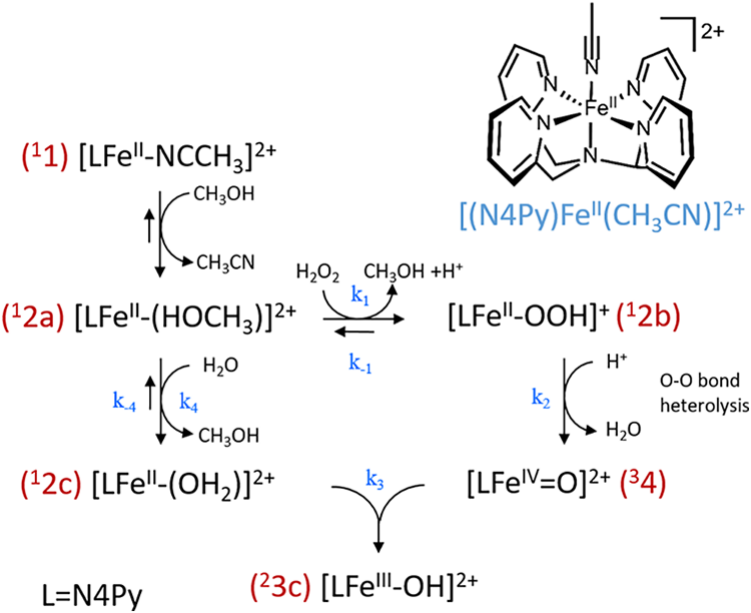
Reactions Following the Addition of Stoichiometric H_2_O_2_ to **1** in Methanol Note that in the Fe(II) oxidation state, the coordination of the solvent (CH_3_OH and H_2_O), as shown for **2a** and **2c**, is favored thermodynamically, while in the Fe(III) oxidation state, the CH_3_O^–^ and HO^–^ bound complexes are lower in energy, based on DFT calculated energies; vide infra.

In methanol, exchange of the acetonitrile ligand of **1** with methanol is immediate (from **1** to **2a**, Scheme 1).^43,45^ A preference for the sixth ligand for [(N4Py)Fe(II)] follows the order CH_3_CN ≫ H_2_O > HOCH_3_, and hence displacement of aquo and methanol/methoxido ligands by H_2_O_2_ is rapid, in contrast to the rate of exchange in acetonitrile.^45^

In this contribution, we make use of the higher rates of ligand exchange in methanol compared to those in acetonitrile to demonstrate that heterolytic cleavage of the O–O bond of a putative Fe(II)-OOH species (**2b**) generates an Fe(IV)=O species (**4**) directly. The influence of potential side reactions, for example, comproportionation between Fe(IV)=O and Fe(II), spin-crossover/ligand exchange of the Fe(II) complex at various temperatures, and reaction of Fe(IV)=O with H_2_O_2_, is evaluated through experiments and quantum chemical calculations. We show that the temperature dependence of the equilibria between acetonitrile, water, and methanol as the sixth ligand in **1** has a decisive impact on the species observed and, therefore, the catalytic reactivity of **1** with H_2_O_2_ that can be expected.

## Results and Discussion

The extent to which the Fe(II) species can be oxidized to the Fe(IV)=O state, rather than the Fe(III) state, depends on the rates of ligand exchange between the solvent and H_2_O_2_, as well as other reactions such as comproportionation. Hence, the temperature dependence of speciation in methanol is established before exploring reactions with H_2_O_2_.

### Speciation of **1** in Methanol

**Scheme 2:**
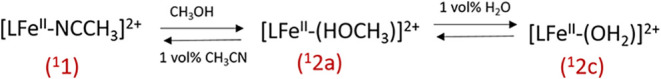
Ligand Exchange Reactions of **1** in Methanol

The differences in redox potentials^45^ and stability calculated by DFT methods (*vide infra*) indicate that **1** is thermodynamically more stable than its H_2_O/HO^–^/MeOH/MeO^–^ bound [(N4Py)Fe(II)] analogues, e.g., **2a**. However, the exchange of the CH_3_CN ligand in **1** for methanol and water occurs immediately upon dissolution in either solvent manifested in changes in UV/vis absorption, cyclic voltammetry, and ESI mass spectrometry.^43^ Addition of 1 vol% (0.25 M) acetonitrile to methanol is sufficient to see an almost complete recovery of the visible absorption spectrum of **1** (Figure S1), i.e., an increase in molar absorptivity of the ^1^MLCT absorption band due to exchange of the methanol ligand with CH_3_CN. One vol% amount of water is sufficient to decrease the visible absorbance in methanol due to exchange of the methanol ligand with H_2_O (Figure S2). These effects highlight the fine balance between the various Fe(II) species in solution and the relatively rapid interconversion between them (Scheme 2).

Consistent with this, the UV/vis absorption spectrum of **1** in methanol shows a strong temperature dependence (Figure S3). As the temperature is decreased to −30 °C, the characteristic visible absorption band of **1** recovers almost completely and is lost reversibly as the temperature is raised to 30 °C. These changes are consistent with a interconversion between the high-spin **2a** and low-spin **1**. Hence, ligand exchange is temperature-dependent in methanol: at room temperature, **2a** is the most abundant complex in solution, while at low temperature it is **1**. The assignment of these temperature-dependent changes in the 6^th^ ligand is confirmed by the absence of temperature dependence in the UV/vis absorption spectrum of **2a**, formed by mixing the ligand N4Py with Fe(II)SO_4_ in methanol (i.e., without CH_3_CN) (Figure S4). Taken together, the data indicate that at the concentrations of **1** used in the present study, i.e., between 0.25 and 1 mM in methanol, at room temperature, ca. 15% of the Fe(II) complex is present as **1** and 85% as **2a**.

### Reaction with Near-Stoichiometric H_2_O_2_

Addition of stoichiometric amounts of H_2_O_2_ to **1** in acetonitrile does not significantly affect its UV/vis absorption spectrum (i.e., <1% of the complex is oxidized to the Fe(III) state) despite that the H_2_O_2_ is consumed (Figure S5). The exchange of the CH_3_CN ligand of **1** with the solvent in methanol and water reduces the barrier to ligand exchange with, e.g., H_2_O_2_, and hence the reaction of **1** with stoichiometric H_2_O_2_ in methanol is faster and proceeds to the Fe(III) state to a greater extent than in CH_3_CN.

**Figure 2:**
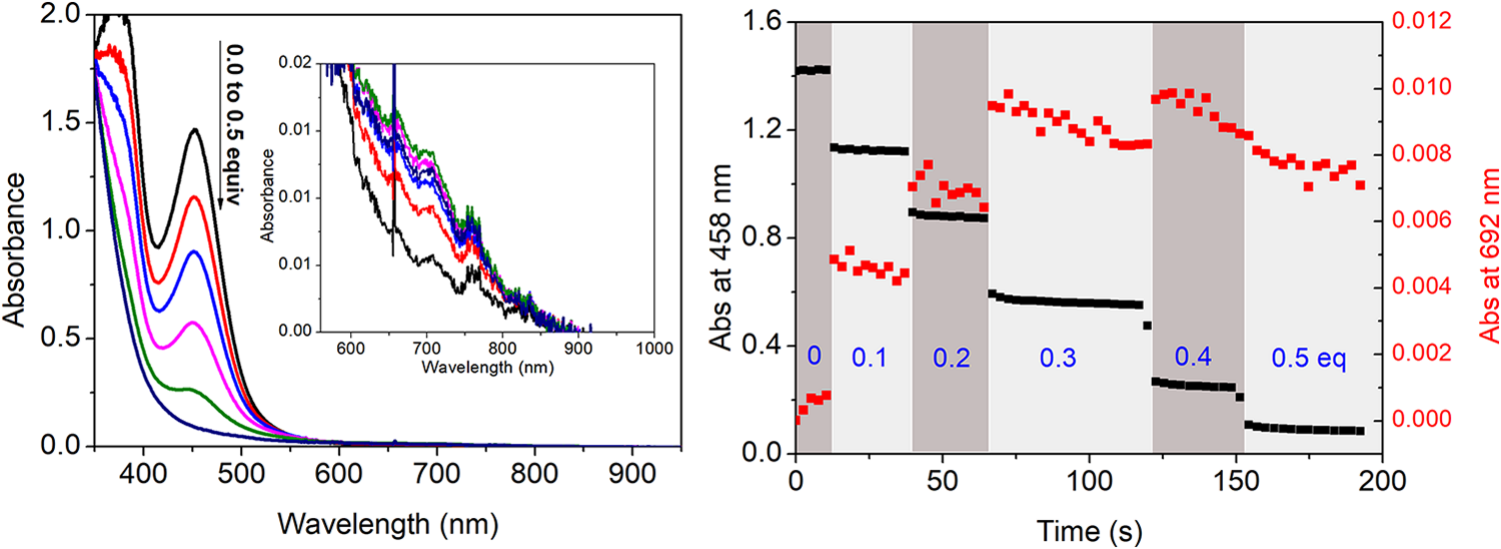
Addition of H_2_O_2_ (50% v/v in H_2_O) in 0.1 equiv increments to **1** (0.5 mM) in CH_3_OH at 21 °C. (left) UV/vis absorption spectra (black: initial, red: 0.1 equiv, blue: 0.2 equiv, magenta: 0.3 equiv, green: 0.4 equiv, navy: 0.5 equiv) with the NIR region shown as an inset. (right) Changes in absorbance at 458 nm (Fe(II), black) and 692 nm (Fe(IV)=O, red).

Accordingly, the addition of H_2_O_2_ in a stepwise manner to **1** in methanol shows a stepwise decrease (20% per step, Figure 2) in absorbance (350–500 nm), corresponding to the net oxidation of the Fe(II) complex to the Fe(III) state. A stepwise appearance of a weak absorption band at 692 nm accompanies the decrease. This absorption band is characteristic of [(N4Py)Fe(IV)=O]^2+^ (**4**). The extent of formation is up to 12% yield of **4** w.r.t. H_2_O_2_ added (Figure 2). UV/vis absorption spectroscopy shows complete oxidation of **1** to, eventually, [(N4Py)Fe(III)(OCH_3_)]^2+^ (**3a**), consistent with the expected 2:1 stoichiometry (**1**:H_2_O_2_), indicating relatively little loss of H_2_O_2_ due to disproportionation under these conditions.^43^ X-band EPR spectroscopy (*S* =  signal at *g* = 2.29, 2.12, and 1.96; see Figure S6) and resonance Raman spectroscopy (Figure S7) show that the major product is **3a**. Resonance Raman spectroscopy (λ_exc_ 355 nm, Figure S7) shows the appearance of the Fe(III)-OCH_3_ stretching band at 554 cm^–1^, the intensity of which increases with the addition of each 0.1 equiv of H_2_O_2_ (up to 0.6 equiv). This band is assigned to ν_str_ Fe-OCH_3_ based on the shift from 554 to 531 cm^–1^ (Δ*ν̃* = 23 cm^–1^) in CD_3_OD (a shift with CH_3_OD is not observed) and the DFT calculated frequency (585 cm^–1^) and corresponding shift (22 cm^–1^) for [(N4Py)Fe(III)(OCH_3_)]^2+^ to [(N4Py)Fe(III)(OCD_3_)]^2+^ (Figure S8). Resonance enhancement of the band at 355 nm is consistent with a ligand-to-metal charge transfer transition, and the Fe-OCH_3_ vibration is close to the same band in the Fe(III)-OCH_3_ form of Bleomycin (530 cm^–1^).^46^ The efficiency in the oxidant suggests that the reaction of H_2_O_2_ with **2a** is faster than with **4** or **3a**.

**Figure 3:**
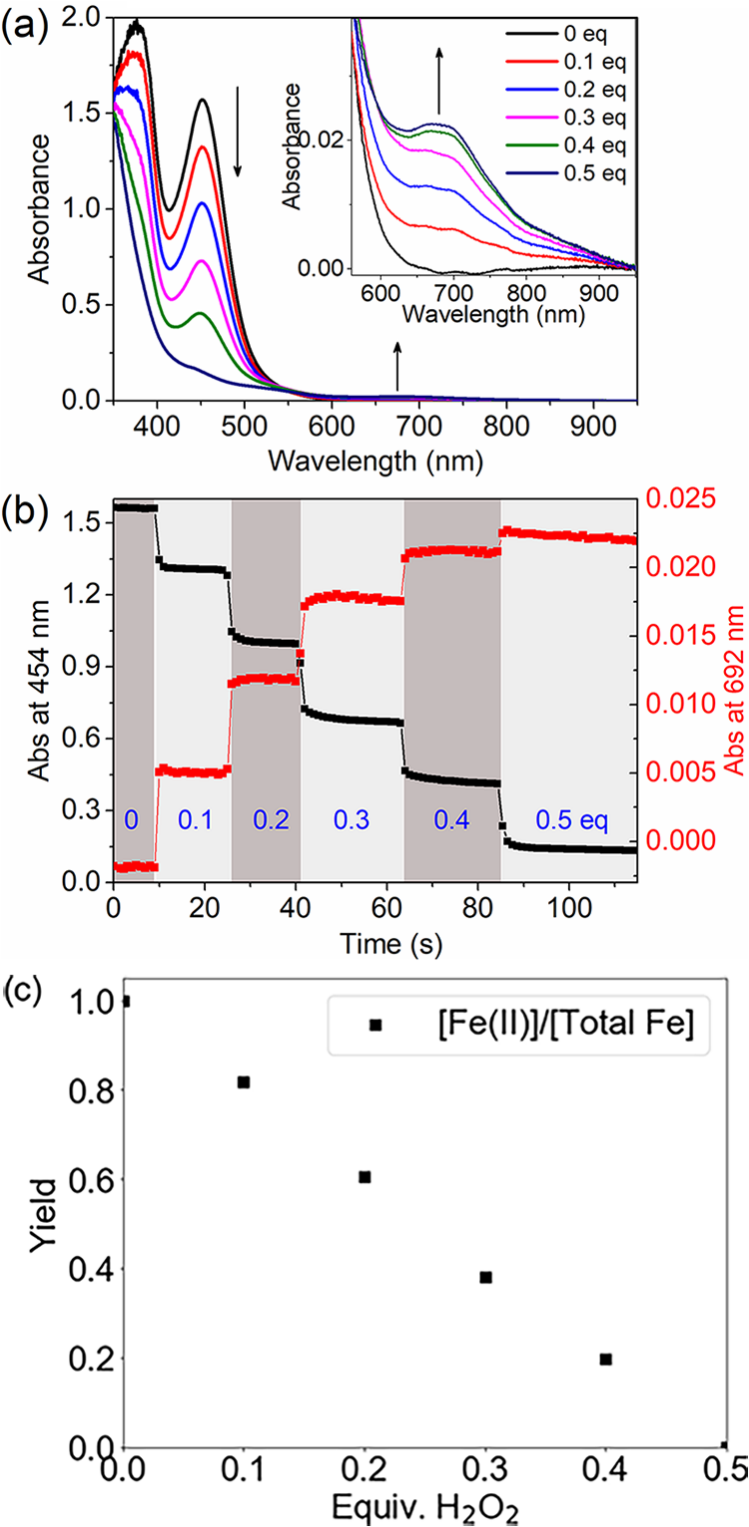
(a) Addition of H_2_O_2_ in steps to **1** (0.5 mM) in CD_3_OD at 21 °C. UV/vis absorption spectra; initial (black) and at 0.1 (red), 0.2 (blue), 0.3 (magenta), 0.4 (green), and 0.5 (navy) equiv H_2_O_2_. Inset: 560–950 nm region. (b) Change in abs. at 454 nm (Fe(II), black) and 692 nm (Fe(IV)=O, red). (c) Extent of oxidation of **1** with respect to the total concentration of iron during stepwise addition of H_2_O_2_ in CD_3_OD.

The <12% yield of Fe(IV)=O obtained with a 2:1 ratio of **1**:H_2_O_2_ in the present study is much less efficient than that obtained earlier with [(TMC)Fe^II^]/H_2_O_2_ (1:1 ratio) and [Fe^II^(bispidine)]/H_2_O_2_ (>1:1) systems.^35,36^ The minor amounts of **4** (Figure 3) obtained could arise either by O–O bond homolysis in an Fe(III)-OOH intermediate (eq 1), as proposed for Fe^II^(bispidine) in methanol (vide supra),^36,46^ or upon O–O bond heterolysis in an Fe(II)-OOH species formed initially (**2b**) (eq 2)
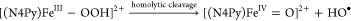
1
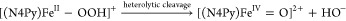
2

**Scheme 3:**
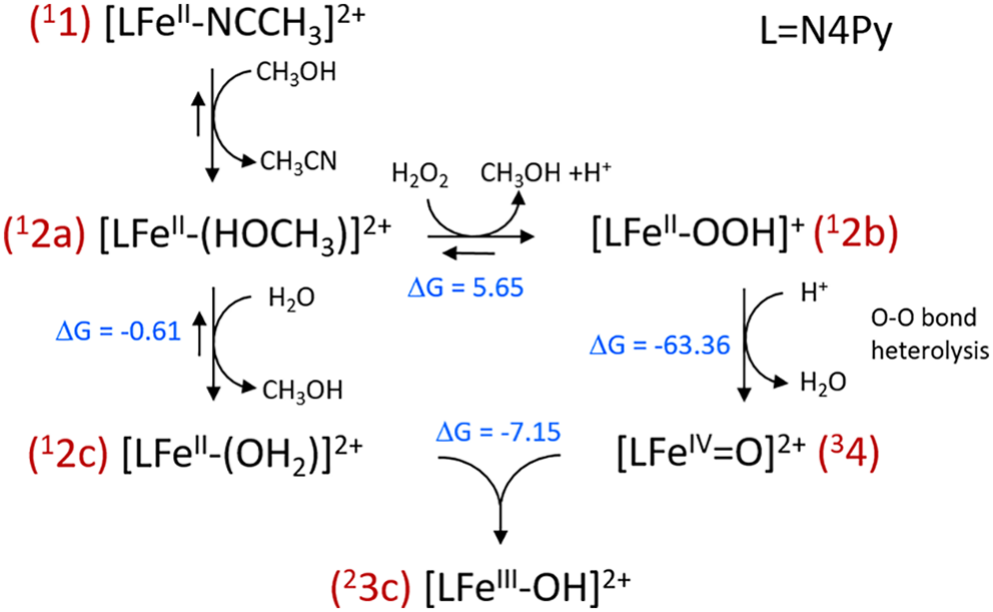
Gibbs Free-Energy Changes (S12g/TZ2P//BP86-D_3_/TDZP) for the Proposed Mechanism

The homolytic pathway (eq 1) can be excluded since the rate at which it proceeds was shown earlier to be 2.2 × 10^–4^ s^–1^ in methanol and is not a kinetically competent pathway on the time scale of oxidations here (within seconds).^43,44^ Indeed, addition of excess H_2_O_2_ (50 equiv) to Fe(III)-OCH_3_ (Figure S9) results in the appearance of the characteristic absorption band of Fe(III)-OOH (λ_*max*_ 550 nm). Its subsequent decay is slow (3.0 × 10^–4^ s^–1^)^43^ and contrasts with the instantaneous formation of **4** upon the addition of H_2_O_2_ to **1** (Figures 2 and 3). Furthermore, the barrier to the O–O bond homolysis in [(N4Py)Fe(III)(OOH)]^2+^ is estimated by DFT to be +19.1 kcal mol^–1^.^47^ Hence, both data reported earlier and in the present report (experimental and calculated) exclude the homolytic pathway from Fe(III)-OOH. The overall 2:1 stoichiometry is consistent with heterolytic O–O bond cleavage in a putative Fe(II)-OOH (eq 2) species, which, according to DFT calculations, is highly exergonic (*vide infra*, Scheme 3). Complex **4** can engage in the oxidation of both alcohol (solvent oxidation) and H_2_O_2_ through HAT,^40,48^ as well as comproportionation with **2c** in methanol. The subsequent comproportionation between the Fe(IV)=O species and Fe(II)-OCH_3_/H_2_O complexes, as shown earlier in H_2_O, can account for the essentially stoichiometric oxidation with the two-electron oxidant H_2_O_2_.^10^ These processes complicate the kinetic analysis of the reaction of **1** with H_2_O_2_. The availability of **4** prepared independently^10^ allows for the relative kinetic competence of various reactions to be established.

### Comproportionation and Solvent Kinetic Isotope Effects

**Figure 4:**
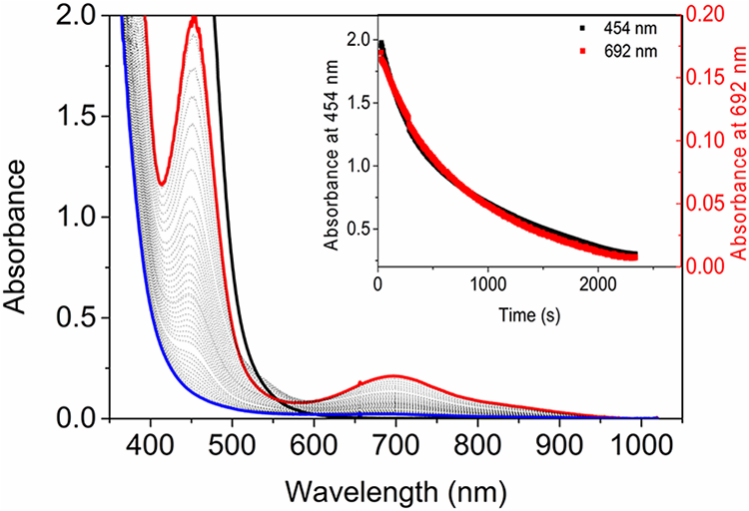
UV/vis absorption spectrum of **1** (1 mM in methanol) before (black line) and 5 s after (red line) the addition of an equimolar/volume solution of **4**; final analytical concentrations of **1** and **4** were 0.5 mM each; final spectrum shown in blue. Inset: Changes in absorbance at 454 nm due to Fe(II) and at 692 nm due to Fe(IV)=O over time.

Comproportionation between **1** and independently prepared **4** proceeds with the visible absorption band of **1** decreasing concomitant with the NIR absorption band of **4** (Figure 4). The rate of comproportionation (*k*_obs_ > 6.0 × 10^–3^ s^–1^, Table S1) is three times that of the oxidation of methanol to formaldehyde by **4** (Figure S10; 1.8 × 10^–3^ s^–1^, Table S1).^49^ Indeed, formaldehyde was not detected in significant amounts following comproportionation.^50^ The final product, [(N4Py)Fe(III)(OCH_3_)]^2+^, was corroborated by resonance Raman and EPR spectroscopy (Figure S11).

Comproportionation is slower in CD_3_OD (*k*_obs_ = 1.3 × 10^–3^ s^–1^, Table S1, apparent KIE = 4.6) but is still 20 times faster than the rate of oxidation of CD_3_OD by **4**. Notably, the rate of comproportionation increases upon the addition of H_2_O (Figure S12), which indicates that an inner sphere mechanism for electron transfer (e.g., HAT) between **2c** and **4** occurs rather than between **4** and **2a**. It should be noted that H_2_O_2_ is added as a (50 wt%) solution in water. Addition of excess H_2_O_2_ will also result in the addition of excess H_2_O, accelerating comproportionation.

Comproportionation of **1** with **4** in H_2_O was reported^10^ earlier and in the present study (Figure S1), and the reaction in H_2_O (*k*_obs_ = 1.12 s^–1^, Figure S13) is over 1000 times faster than in methanol but nevertheless still shows a KIE of ca. 3 (in D_2_O, *k*_obs_ = 0.4 s^–1^, Figure S1). These data support that comproportionation is between **4** and **2c** and that the KIE observed in CH_3_OH is due to rapid H/D equilibration between methanol and water. The conclusion that **2c**, and not **2a**, reacts with **4** is further supported by the observation that in methanol at −30 °C, comproportionation does not occur due to the recovery of the CH_3_CN-bound complex **1** at that temperature (*vide supra*). The absorption bands for both **1** and **4** do not change until 10 vol% H_2_O (*k*_obs_ = 3.9 × 10^–3^ s^–1^, Table S1) has been added, and the rate of change increases further with 50 vol% H_2_O added (*k*_obs_ = 4.3 × 10^–2^ s^–1^, Table S1, Figure S14).

The data indicate that conversion of **1** to the final Fe(III) species proceeds through ligand exchange, from **2a** to **2b** (Fe(II)-HOCH_3_ to Fe(II)-OOH), followed by O–O bond heterolysis to form **4** and OH^–^ (within the mixing time, <1 s). The formed Fe(IV)=O then comproportionates with [(N4Py)Fe(II)(OH_2_)]^2+^ (**2c**) to generate [(N4Py)Fe(III)(X)]^2+^ (X = OH or OCH_3_, Scheme 1). Therefore, the 2:1 stoichiometry (**1**:H_2_O_2_) seen in the conversion of **1** to an Fe(III) species is mainly due to equilibration of **2a** in methanol with adventitious H_2_O, and with H_2_O_2_.

This mechanism predicts that the concentration of **4** will build up significantly in methanol due to its slow reaction with the methanol-bound Fe(II) complex (**2a**). Solvent deuteration (KIE = 4) retards comproportionation sufficiently to allow for a higher concentration of **4** to accumulate (Figure 3). In methanol, the reaction of H_2_O_2_ with **2a** is much faster (<1 s) than the reaction of H_2_O_2_ with **4** (*k*_obs_ = 1.0 × 10^–2^ s^–1^ in CH_3_OH and 1.0 × 10^–3^ s^–1^ in CD_3_OD),^43^ and hence the latter reaction cannot compete, leading to a buildup of **4**. Furthermore, although **4** accumulates after each substoichiometric addition of H_2_O_2_, comproportionation with **2c** reduces the concentration of **4** again (Figure 3).

### Impact of Temperature and Ligand Exchange Rates

**Figure 5:**
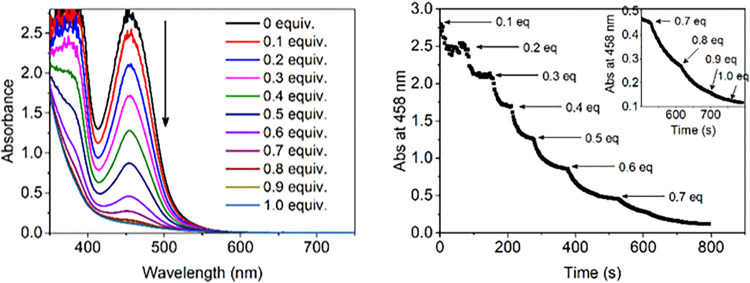
(Left) UV/vis absorption spectra of the addition of H_2_O_2_ in steps at −30 °C to **1** (0.5 mM) in CH_3_OH. (Right) Changes in abs. at 458 nm for 0.7–1.0 equiv shown in detail in the inset.

A common approach to stabilizing reactive species is to generate them at a low temperature. At −30 °C in methanol, stepwise addition of H_2_O_2_ to **1** results in the concomitant stepwise decrease in visible absorbance due to oxidation to the Fe(III) state (Figure 5). In contrast to ambient conditions, complete oxidation requires >0.5 equiv H_2_O_2_. In addition, the absorbance in the NIR region does not increase, i.e., **4** does not accumulate. Only 66% of **2a** is oxidized to the Fe(III) state by the addition of 0.5 equiv H_2_O_2_, but the extent increases to 77 and >90% when 5 and 10 vol % H_2_O is present, respectively (Figures S15 and S16).

With [(N4Py)Fe(II)-HOCH_3_]^2+^ instead of **1** (i.e., CH_3_CN is not present even as a ligand), only 0.5 equiv of H_2_O_2_ is necessary for full oxidation of [(N4Py)Fe(II)-HOCH_3_]^2+^ in MeOH at −40 °C (Figure S17). Hence, without the CH_3_CN ligand, exchange of the methanol ligand of [(N4Py)Fe(II)-HOCH_3_]^2+^ with H_2_O_2_ is sufficiently rapid for the oxidation of the Fe(II) complex to compete with other reactions. These data indicate that solvent exchange reactions are retarded sufficiently for the reaction of **4**, formed initially, with H_2_O_2_. In addition, disproportionation of H_2_O_2_ via a Fe(III)-OOH species can occur.^44^

The differences in behavior at low and ambient temperatures can be rationalized by consideration of the rates of each of the individual steps in the multistep reaction, together with the temperature-dependent equilibrium between **1** and **2a** in both methanol (Scheme 3) and H_2_O (Figure S18). The singlet–quintet spin-state switching accompanying ligand exchange is manifested by a reversible change in the molar absorptivity of the MLCT bands at 380 and 454 nm as the temperature decreases and increases (Figure S19). At −30 °C (Table S1), both the oxidation of **2a** (1.0 × 10^–2^ s^–1^, due to its low concentration), and comproportionation between **2c** and **4** (*vide supra*, Figure S14) are slower, and hence oxidation of **2a** via a comproportionation pathway is not significant. Hence, H_2_O_2_ is likely consumed by other processes, e.g., reaction with Fe(IV)=O and direct reaction between Fe(III)-OOH and H_2_O_2_ as shown earlier.^40,43^ As is the case in CH_3_CN at room temperature, in methanol at −30 °C, the CH_3_CN/H_2_O_2_ ligand exchange (in **1**) is slow and is the rate-determining step. Hence, any **4** produced can react with H_2_O_2_ present, which rationalizes why a greater number of equivalents of H_2_O_2_ are needed at −30 °C. Notably, with excess H_2_O_2_, the Fe(III)-OOH species is formed much more slowly than at 21 °C and persists for ≫1 h (Figure S20). The acceleration observed with H_2_O together with full oxidation of **1** is consistent with the acceleration of methanol/water ligand exchange and the increased opportunity for comproportionation between **2c** and **4**.

### DFT Calculations

The changes in free energy over each step (Scheme 3) indicate that upon dissolving in methanol, **1** (*S* = 0) undergoes ligand exchange as observed experimentally. Ligand exchange from **2a** (*S* = 0) to form **2b** (*S* = 1) is endergonic (5.65 kcal mol^–1^); however, heterolysis of the O–O bond yielding **4** (*S* = 1) is highly exergonic (−63.36 kcal mol^–1^), rationalizing the absence of spectroscopic evidence for the putative intermediate **2b**. Adventitious water, as well as water that is added with H_2_O_2_ (50 wt % in H_2_O), facilitates ligand exchange to yield [(N4Py)Fe(II)(OH_2_)]^2+^ (**2c**, *S* = 0) from **2a** (−0.61 kcal mol^–1^). As reported earlier,^10^ comproportionation of **2c** (*S* = 0) and **4** (*S* = 1) to form [(N4Py)Fe(III)(OH)]^2+^ (**3c**, *S* = ) is also highly exergonic (−7.15 kcal mol^–1^). [(N4Py)Fe(III)(OCH_3_)]^2+^ (**3a**, *S* = ), but not **3c**, is observed by EPR and Raman spectroscopy due to the exergonicity of ligand exchange in favor of the solvent methanol.^43^

**Figure 6:**
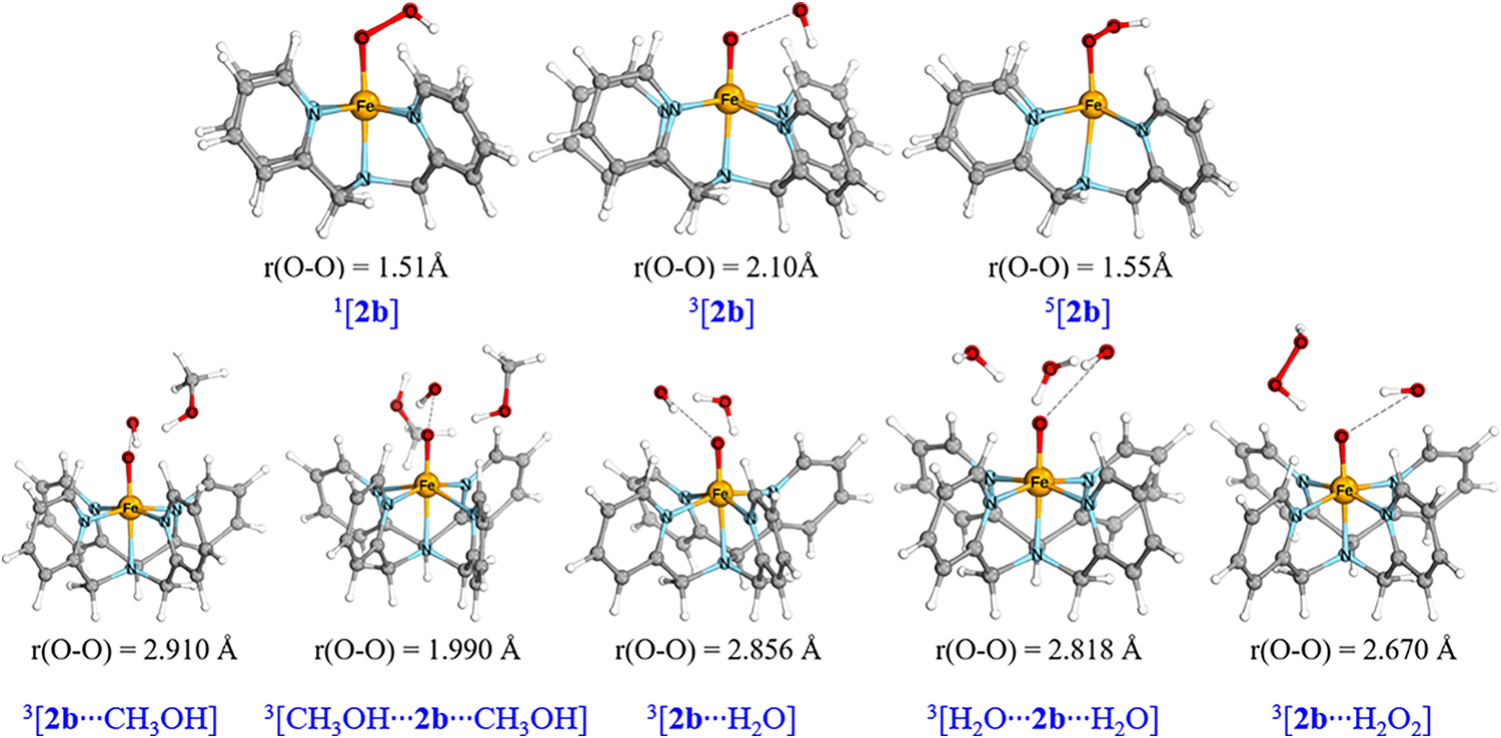
(Top) Optimized geometries (BP86-D_3_/TDZP) for **2b** in different spin states. (Bottom) Corresponding optimized geometries of **2b** with S = 1 with various adducts, including one or two methanol molecules, one or two H_2_O molecules, and a H_2_O_2_ molecule; only four representative structures are shown (the complete table is shown in Table S4).

**Figure 7:**
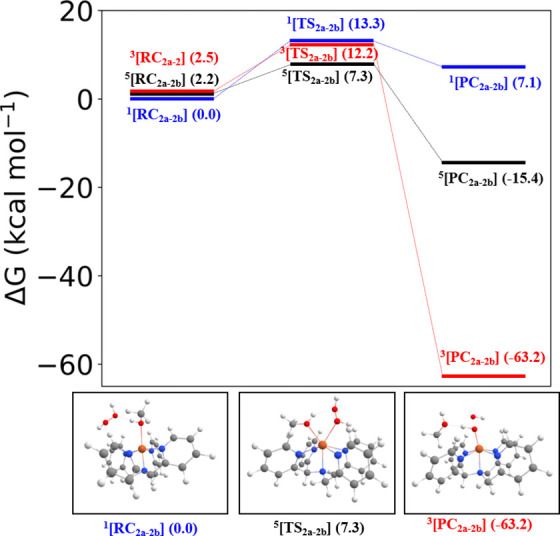
(Top) Energy profile (in kcal mol^–1^) for the ligand exchange (the reaction of **2a** with H_2_O_2_ to form [(N4Py)Fe(II)(HOOH)]^2+^), obtained with the S12g/TZ2P//BP86-D3/TDZP level. (Bottom) Corresponding structures are shown below.

The heterolysis of the O–O bond was investigated by DFT calculations. The low (*S* = 0) and high (*S* = 2) spin states of **2b** are lowest in energy and have essentially the same energy with the low-spin state (*S* = 0) only 2.9 kcal mol^–1^ higher than the *S* = 2 state. The intermediate spin (*S* = 1) state is 17.5 kcal mol^–1^ higher in Gibbs free energy. The activation energy for the heterolysis of **2b** is 1.3 kcal mol^–1^ at *S* = 2 and the reaction is exergonic (Figure S21). Unfortunately, the transition state for heterolysis at *S* = 1 was not obtained but already shows the cleavage of the O–O bond during the geometry optimization (i.e., a spontaneous process). This is consistent with its transient nature and the large exergonicity at *S* = 1 (−63.36 kcal mol^–1^, Scheme 3). The influence of solvent and different adducts in solution on heterolysis was considered by adding CH_3_OH (solvent), H_2_O (residues in methanol and 50 wt % H_2_O_2_), and H_2_O_2_ (added). Seven different conditions were calculated by altering the hydrogen bond acceptor and donor to Fe(II)-OOH (Table S4). In all cases, the triplet state is the lowest in energy (Table S3) and all O–O bonds show spontaneous heterolytic cleavage upon geometry optimization (Figure 6 and Table S4). Overall, the conversion of **2b** to **4** and hydroxide is energetically downhill and has negligible activation energy. Hence, temperature will only affect the outcome of the reaction by its effect on prior ligand exchange steps, i.e., reaction of **1** rather than **2a** with H_2_O_2_ to form the transient **2b**. The barriers for the exchange of the methanol ligand of **2a** with H_2_O_2_ were calculated at all three spin states (Figure 7), which showed that the lowest barrier is observed for the *S* = 2 (high) spin state. This exchange occurs with a relatively high activation free energy (7.3 kcal mol^–1^) and is the rate-determining step. After this exchange, the complex then moves toward CH_3_OH-bound **2b** (Figure 6, bottom) for which the *S* = 0 state is again lowest in energy. Therefore, between the exchange TS and the CH_3_OH-bound **2b**, a minimum energy crossing point (MECP)^51,52^ must be passed.^53^

## Conclusions

Generation of (L)Fe(IV)=O species from the corresponding Fe(II) complexes of the ligands TMC and bispidine in relatively high yield upon reaction with stoichiometric H_2_O_2_ indicated heterolysis of the O–O bond of putative (L)Fe(II)-OOH intermediates. In the present study, we show that this pathway is followed by Fe(II) complexes of the ligand N4Py and that the low yield of [(N4Py)Fe(IV)=O]^2+^ is due to competition with other reactions. The lower potency of [(TMC)Fe(IV)=O]^2+^ toward HAT, compared to that of [(N4Py)Fe(IV)=O]^2+^,^8,54^ means that comproportionation of the ferrous and ferryl complexes has less impact. Although an initial O–O bond heterolysis in [(bispidine)Fe(II)(OOH)]^2+^ to form [(bispidine)Fe(IV)=O]^2+^ in methanol is likely, it can be masked by homolysis of the O–O bond of [(bispidine)Fe(III)-OOH]^2+^, subsequent comproportionation between [(bispidine)Fe(II)–OH]^2+^ and Fe(IV)=O, as well as the HAT between H_2_O_2_ and Fe(IV)=O. We reported earlier that homolytic cleavage of the O–O bond of [(N4Py)Fe(III)(OOH)]^2+^ is not kinetically relevant^44^ and in the present study, we show that comproportionation and HAT reactions of [(N4Py)Fe(IV)=O]^2+^ are important but can be slow enough in methanol to allow for the observation of the initially formed [(N4Py)Fe(IV)=O]^2+^. DFT calculations support the facile formation of [(N4Py)Fe(IV)=O]^2+^ from a putative [(N4Py)Fe(II)-(OOH)]^+^ species; however, we also show through temperature-dependent studies that ligand exchange equilibria in the Fe(II) oxidation state have a major impact on reactivity and reaction outcomes.

Despite being present in only submillimolar concentrations, the driving force for binding of CH_3_CN is strong, which is driven exchange of methanol/water ligands with acetonitrile temperature is lowered. These data rationalize observations made in acetonitrile, also where a large excess of H_2_O_2_ is required to oxidize the complex to the Fe(III) state. Furthermore, we show that ligand exchange equilibria prior to reaction with H_2_O_2_ can greatly impact the temperature dependence of reactions. Finally, understanding the overall mechanism for the formation of [(N4Py)Fe(IV)=O]^2+^ by heterolytic O–O bond cleavage is important in strategies to harness the benefit of rebound reactions by [(L)Fe(IV)=O]^2+^ complexes in regenerating Fe(II) species. Controlling the relative rates of ligand exchange and comproportionation is essential in achieving a hydroxyl radical free Fe(II)/Fe(IV)=O redox cycle.

## Experimental Section

[(N4Py)Fe(II)(NCCH_3_)](ClO_4_)_2_ (**1**) and [(N4Py)Fe(IV)=O](PF_6_)_2_ (**4**) were available from previous studies.^10,43^ Solvents and chemicals were obtained from Sigma-Aldrich and used without further purification. Solvents for spectroscopy were UVASOL (Merck) grade. H_2_O_2_ (50 wt % in water; Aldrich Chemicals) was diluted as required in methanol.

### Caution

The concentration or drying of solutions that may contain H_2_O_2_ should be strictly avoided. Before drying or concentration, peroxide test strips should be used to confirm that H_2_O_2_ is present or not, and where required, neutralization on solid NaHSO_3_ or an alternative appropriate reducing agent should be performed. Suitable protective safeguards should be in place at all times when working with H_2_O_2_, due to the risk of explosion.

### Caution

When working with perchlorate salts, suitable protective safeguards should be in place at all times due to the risk of explosion. Perchlorate salts should be handled in small (milligram) quantities and used only where necessary.

### Physical Methods

UV/vis absorption spectra were recorded by using a Specord600 (AnalytikJena) spectrometer in quartz (1 cm path length) cuvettes. EPR spectra (X-band, 9.46 GHz) were recorded on a Bruker ECS106 or EMX Nano spectrometer at 77 K (in liquid N_2_). Samples (0.5 mL) were transferred from a solution determined by UV/vis absorption spectroscopy to 3-mm-diameter quartz EPR tubes and flash-frozen in liquid N_2_ immediately. Raman spectra at 355 nm are reported earlier.^45^ Spectra were calibrated using acetonitrile/toluene, 50:50 (v/v), and processed (baseline correction/solvent subtraction where necessary) with Spectragryph V.1.15.

### Computational Details

ADF and QUILD^55^ were used to perform computational studies.^43^ Geometries were optimized and frequency calculated using an unrestricted density functional BP86-D_3_^56^ with a triple-ζ valence plus polarization basis set on iron combined with a double-ζ valence plus polarization on all other atoms (TDZP). Single-point energy calculations were made on these geometries with the S12g spin-state consistent functional^57^ in a triple-ζ valence plus double polarization (TZ2P) basis set. Free-energy corrections (ΔG) were obtained from the BP86-D_3_ data and corrected for zero-point energy (ZPE); thermal and entropic corrections were made from frequency calculations at 298 K. The solvation energy was considered with the solvent methanol using the COSMO solvation model, implemented in ADF.^58^ Molecular depictions for all structures were made using the IboView program (iboview.org).^59,60^

## Supporting Information Available

Data for experimental section; colorimetric quantification of formaldehyde; complete electronic and Gibbs energy data; optimized structures; and Cartesian coordinates for all structures (PDF)
