# Supplementary material for: Generation of [(N4Py)Fe(IV)=O]2+ through Heterolytic O–O Bond Cleavage in [(N4Py)Fe(II)(OOH)]+
Source: Inorg Chem. 2025 May 2;64(19):9408–17. doi: 10.1021/acs.inorgchem.4c05172 (PMC12093294; doi:10.1021/acs.inorgchem.4c05172)
Supplement: Supplementary file 1 — ic4c05172_si_001.pdf [file ic4c05172_si_001.pdf]

# Supporting information

## Generation of $[(N4Py)Fe(IV)=O]^{2+}$ through Heterolytic O-O Bond Cleavage in $[(N4Py)Fe(II)(OOH)]^{+}$

Juan Chen,[a] Andy S. Sardjan,[b] C. Maurits de Roo,[b] Marika Di Berto Mancini,[b] Apparao Draksharapu,[b,c] Davide Angelone,[b] Ronald Hage,[b] Marcel Swart,[d,e,*] and Wesley R. Browne[b,*]

[a]Department of Applied Chemistry, School of Science, Northwestern Polytechnical University, Xi'an, Shaanxi 710072, China

[b]Molecular Inorganic Chemistry, Stratingh Institute for Chemistry, Faculty of Science and Engineering, University of Groningen, Nijenborgh 3, 9747 AG, Groningen, The Netherlands

[c]current address: Southern Laboratories - 208A, Department of Chemistry, Indian Institute of Technology Kanpur, Kanpur-208016, India

[d]ICREA, Pg. Lluis Companys 23, 08010 Barcelona, Spain

[e] Institut de Quimica Computacional i Catalisi (IQCC), Departament de Quimica, Universitat de Girona, Campus Montilivi, 17003, Girona, Catalonia, Spain

E-mail: marcel.swart@udg.edu, w.r.browne@rug.nl

## Colorimetric quantification of formaldehyde

The formation of formaldehyde was quantified as described in the literature.(Nash, T. Biochem. J. 1953, 55, 416–421.) The colorimetric reagent was prepared by dissolving $NH_4OAc$ (15 g, 0.19 mol), acetic acid (0.3 mL, 5.4 mol) and pentane-2,4-dione (0.2 mL, 1.9 mol) in 100 mL water. Take 1 mL reaction solution, diluted 10 times (1 mL solution dissolved in 9 mL water, the concentration of formaldehyde should not exceed 8 $\mu$g of formaldehyde per mL), then mixed with the other 10 mL of colorimetric reagent. The mixture was put into a 31 °C water bath. Every 2 min, 1 mL mixture was transferred to the cuvette and check the absorbance at 420 nm, after around 35 min, the absorbance will reach to its maximum and stable for ca. 10 min. This maximum absorbance was used to calculate the concentration of formaldehyde using equation (1).

$$C = \frac{A_{420nm}}{(L \times \varepsilon_{420nm})} \times 20 \ (1)$$

Where C is the concentration of formaldehyde in the reaction, $A_{420nm}$ is the maximum absorbance of the mixture at 420 nm, L is the path length of the cuvette, $\varepsilon_{420nm}$ is the molar absorptivity of diacetyldihydrolutidine (DDL), which was determined by calibration with solutions containing known amounts of formaldehyde.

## Supplementary experimental data

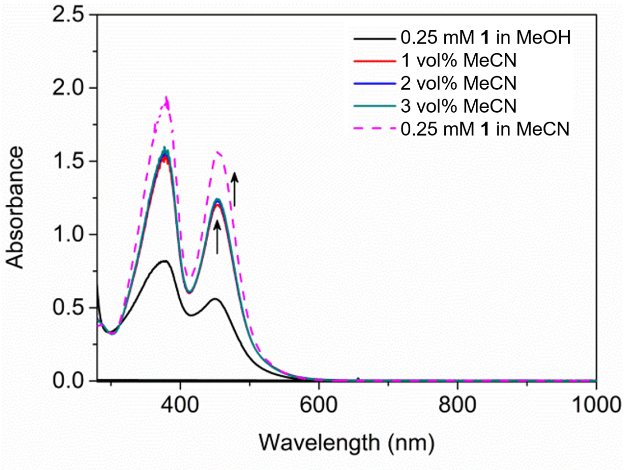


**Figure S1.** UV/vis absorption spectrum of **1** (0.25 mM) in methanol (black), and after addition of 1-3 vol% acetonitrile, and in acetonitrile (magenta).

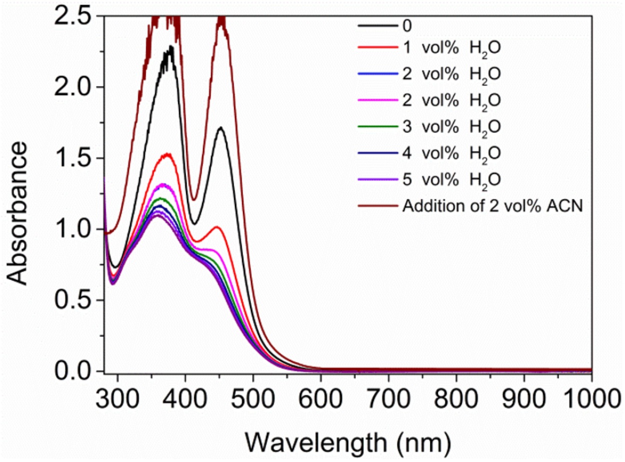


**Figure S2.** UV/vis absorption spectrum of **1** (0.5 mM) in methanol (black), with an addition of 1-5 vol% $H_2O$, followed by addition of 2 vol% acetonitrile.

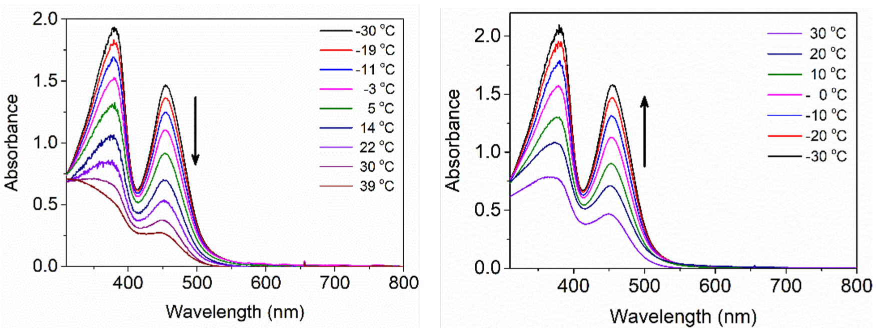


**Figure S3.** (left) UV/vis absorption spectrum of **1** (0.25 mM) in deoxygenated methanol with increase in temperature (-30 °C to 39 °C). (right) UV/vis absorption spectrum of **1** in methanol with decrease in temperature (30 °C to -30 °C).

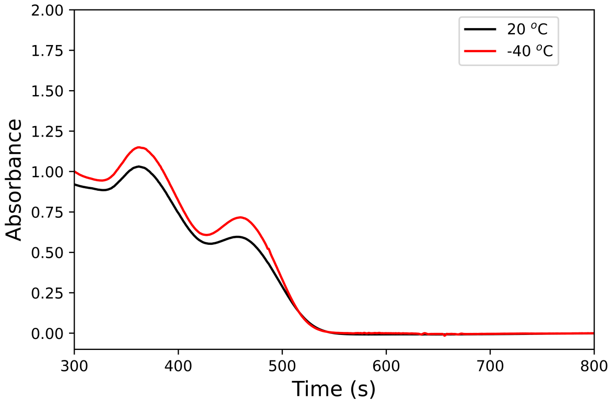


**Figure S4.** UV/vis absorption spectra of **2a** (0.2 mM) in MeOH at room temperature (black) and at -40 °C (red).

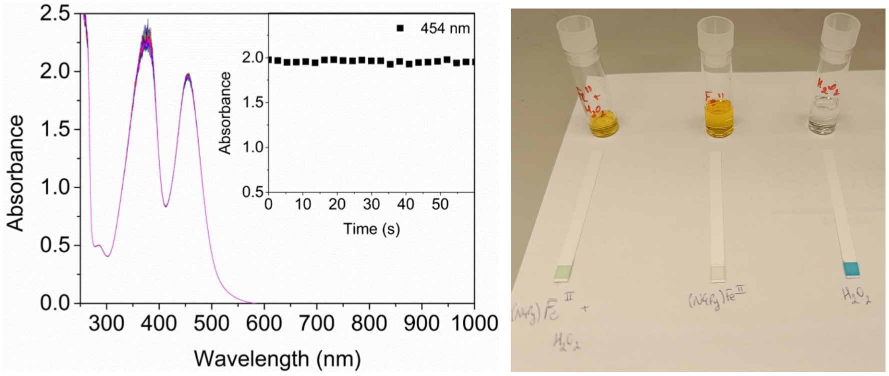


**Figure S5.** Left: UV/vis absorption spectrum of **1** (0.25 mM) in acetonitrile after addition of 0.5 equiv. $H_2O_2$. Right: 0.5 eq. of $H_2O_2$ was added to 0.25 mM **1** in $CH_3CN$ (left) and to $CH_3CN$ alone (right). A solution of only 0.25 mM **1** was used for comparison (center). 500 $\mu$l of each solution was mixed with 500 $\mu$l of water before dropping (10 $\mu$l) the solutions onto $H_2O_2$ test stripes (MQuant$^{TM}$ Peroxide test stripes 0.5-25 mg/l range).

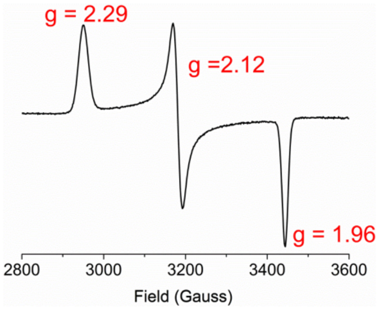


**Figure S6.** X-band (9.46 GHz) EPR spectrum of the flash frozen (at 77 K) solution of **1** in methanol after addition of 0.5 equiv. $H_2O_2$.

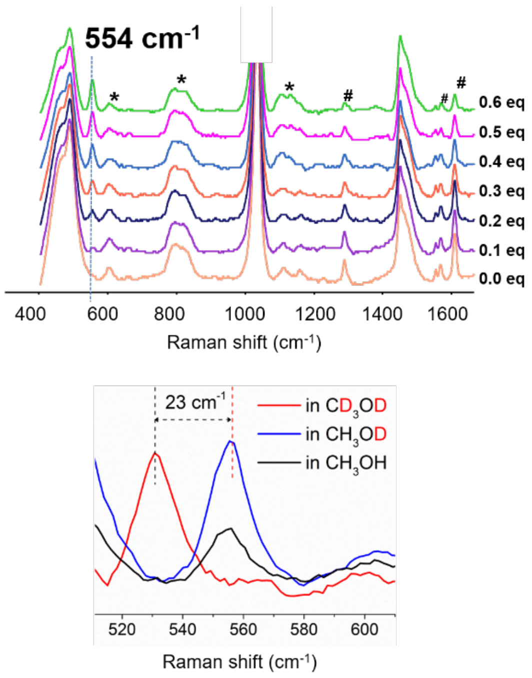


**Figure S7.** (Top) Resonance Raman spectrum ($\lambda_{exc}$ 355 nm) of **1** in methanol before and after the addition of 0.1 to 0.6 equiv. $H_2O_2$. The band at 1555 cm$^{-1}$ is due to $O_2$. *Raman scattering from quartz cuvette. # Raman bands of complex **1**. (bottom) Expansion of 510-570 cm$^{-1}$ region to show 554 cm$^{-1}$ band in $CH_3OH$, $CH_3OD$ and $CD_3OD$.

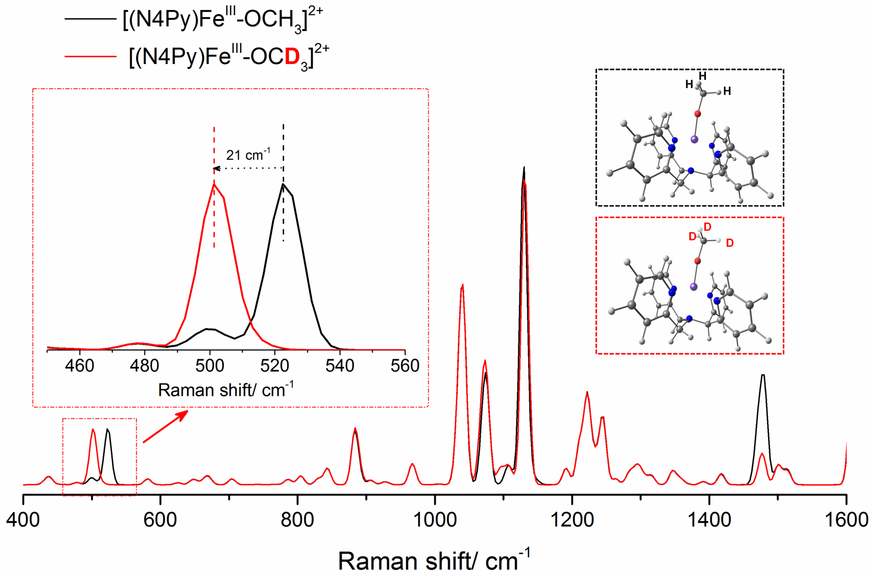


**Figure S8.** Calculated Raman spectra {(B3LYP/*LANL2TZ+(f) on Fe and 6-311+G(d,p) on C,H,O,N*)} of complexes $[(N4Py)Fe^{III}\text{-}OCH_3]^{2+}$ (black) and $[(N4Py)Fe^{III}\text{-}OCD_3]^{2+}$ (red). Inserts are the expansion on Fe-OC(H/D)$_3$ stretching mode (left) and optimized structures for both (right).

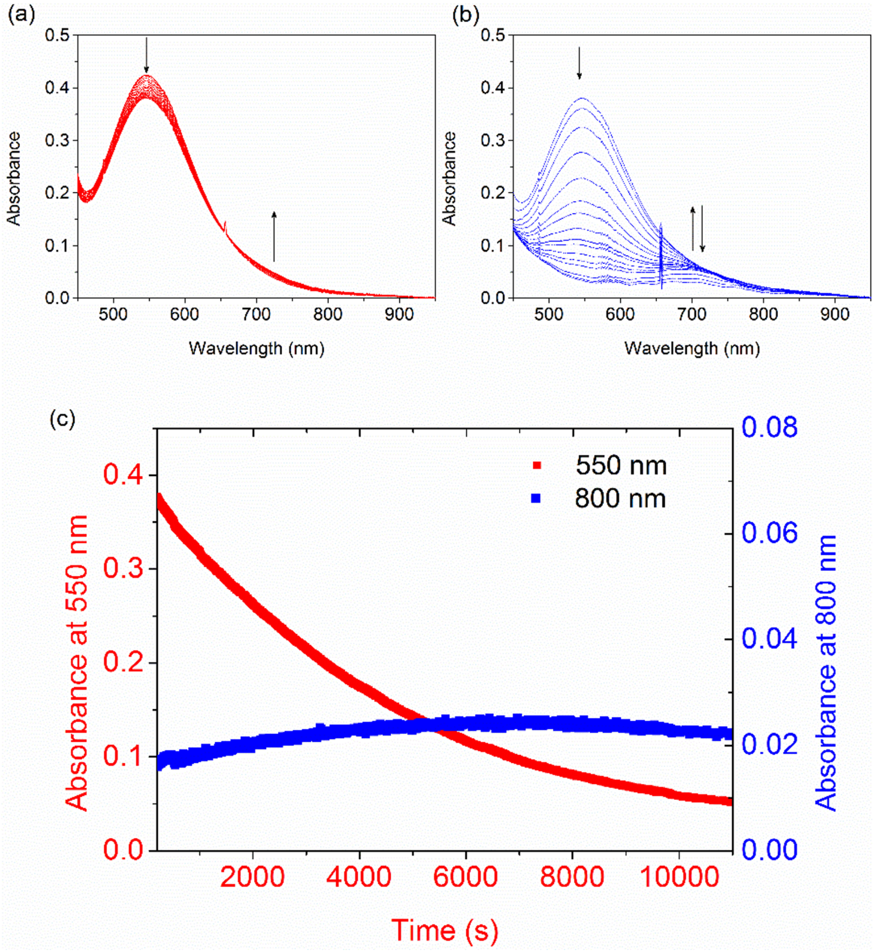


**Figure S9.** UV/vis absorption spectrum of $[(N4Py)Fe^{III}\text{-}OCH_3]^{2+}$ (0.5 mM) in $CD_3OD$ after addition of 50 equiv. $H_2O_2$ at 21 °C, (a) 50-200 s, (b) 200-11000 s, (c) absorbance at 550 nm and 800 nm over time, respectively.

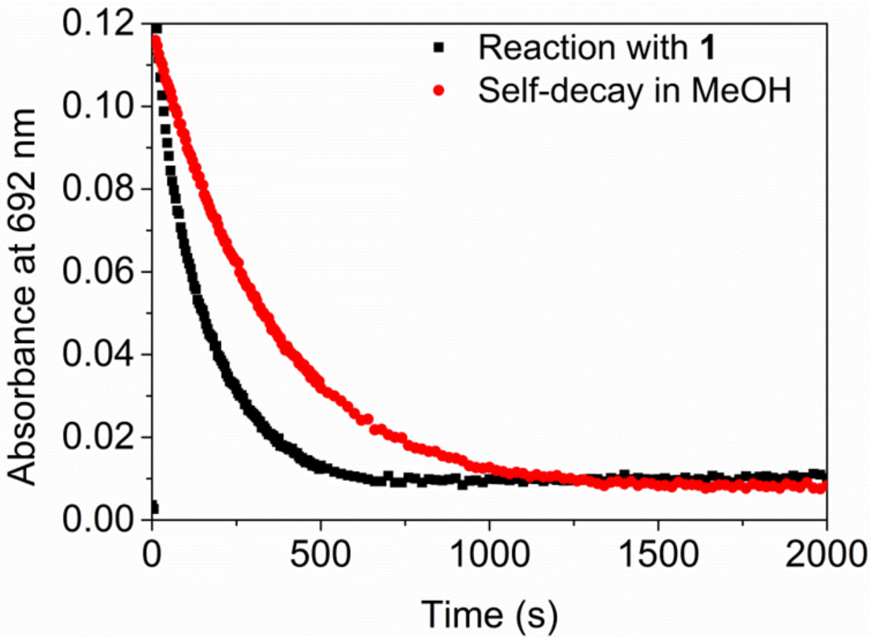


**Figure S10.** Absorbance over time at 692 nm following addition of 1 equiv. **4** (1 mM, 1 mL) to **1** (1 mM, 1 mL) in methanol (black), compared with the self-decay of **4** in methanol, in which the start point was taken to be where **4** had decayed to the same absorbance.

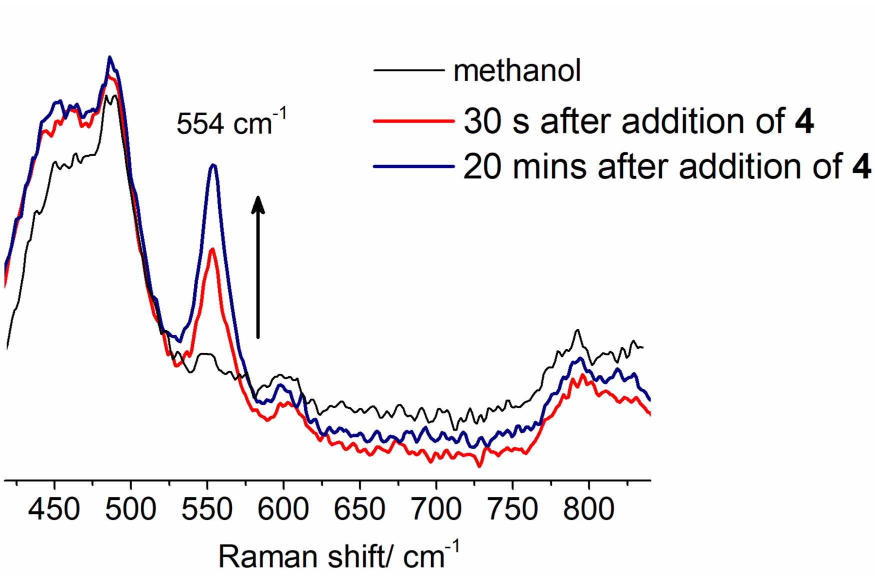


**Figure S11.** Raman spectrum ($\lambda_{exc}$ 355 nm) of **1** (1 mM in 1 mL) in methanol (black) and 30 s (red) and 20 min (blue) and after addition of 1 equiv. **4** (1 mM in 1 mL, final concentration 0.5 mM).

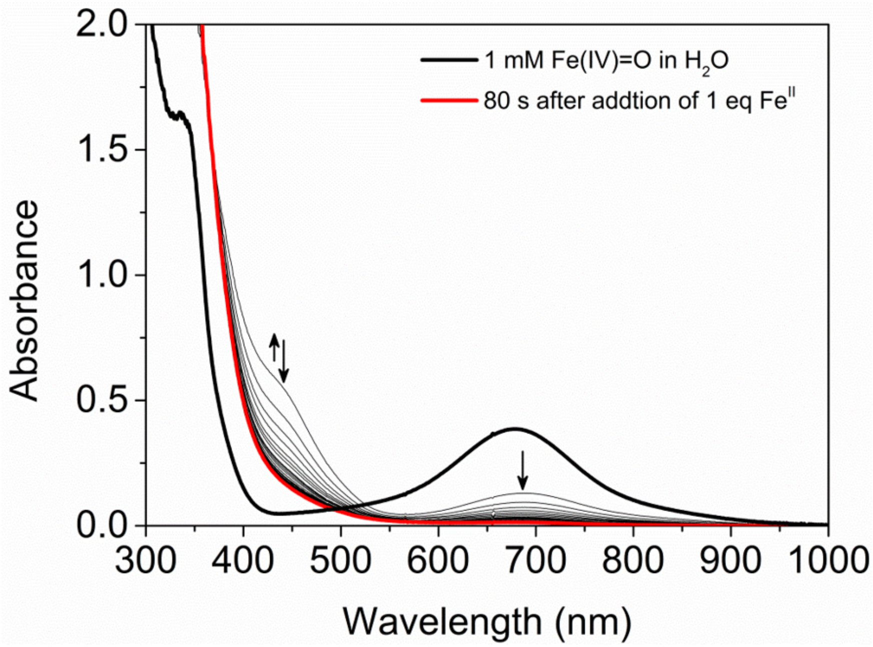


**Figure S12.** UV/vis absorption spectrum of **4** (1 mM, 1 mL) in $H_2O$, after addition of 1 equiv. $[(N4Py)Fe^{II}\text{-}Cl]^+$ (**1-Cl**) (1 mM, 1 mL) in methanol. **1-Cl** was used due to its greater solubility and was fully dissociated to form $[(N4Py)Fe^{II}\text{-}OCH_3]^+$ in methanol.

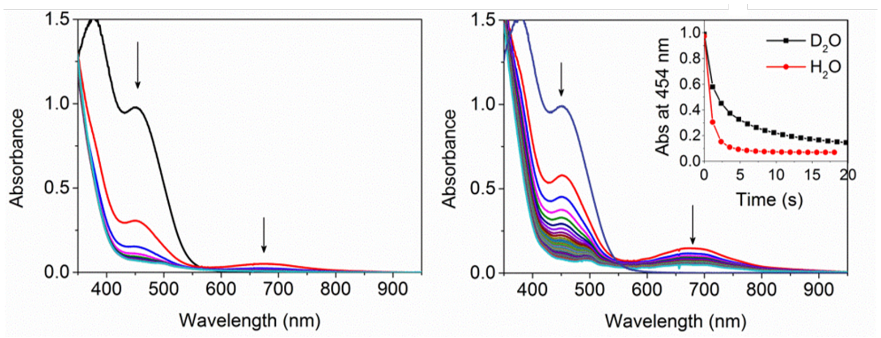


**Figure S13.** (left) UV/vis absorption spectrum of **1** (0.5 mM, 1.2 mL) in $H_2O$ after addition of 1 equiv. **4** (1.2 mM, 300 µL) in $H_2O$; (right). UV/vis absorption spectrum of **1** (0.5 mM, 1.2 mL) in $D_2O$ after addition of 1 equiv. **4** (1.2 mM, 300 µL) in $D_2O$.

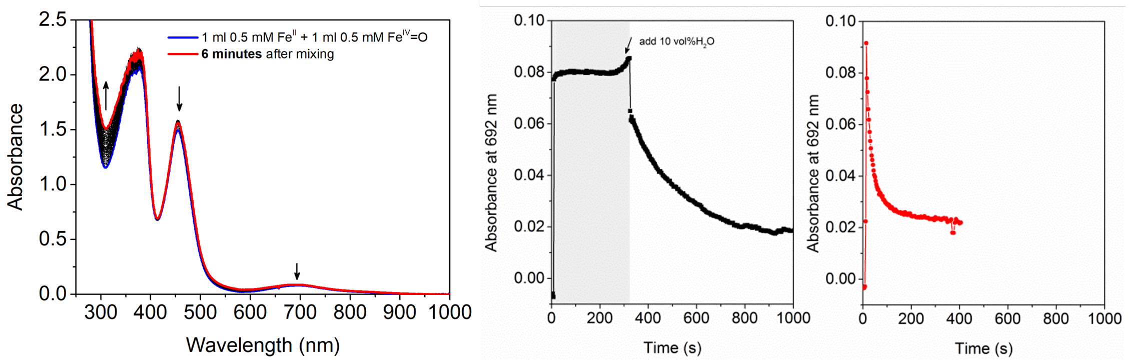


**Figure S14.** (left) UV/vis absorption spectrum of **1** (0.25 mM) with **4** (0.25 mM) in methanol at -30 °C. (middle) Absorbance at 692 nm during the reaction of **1** (0.5 mM, 1 mL) in methanol at -30 °C with **4** (0.5 mM, 1 mL) (grey shadowed), and after addition of 10 vol% of $H_2O$ (black). (right) Absorbance at 692 nm of **1** (0.5 mM, 1 mL) in methanol at -30 °C with addition of **4** (0.5 mM, 1 mL in $H_2O$).

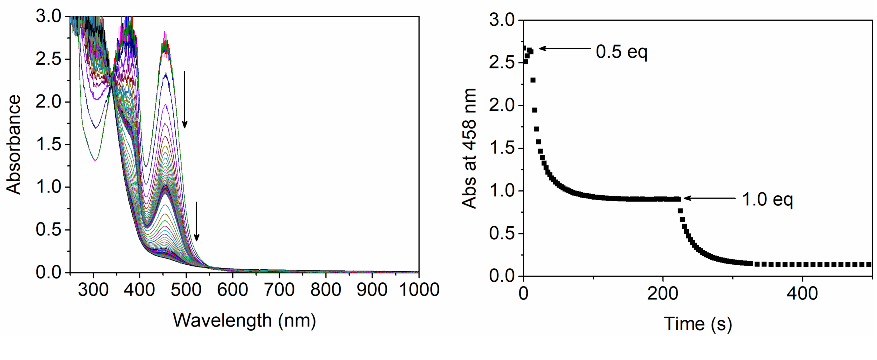


**Figure S15.** (left) UV/vis absorption spectrum of **1** in methanol at -30 °C with two-step addition of $H_2O_2$ (0.5 equiv. for each step). (right) The corresponding change in absorbance at 458 nm over time.

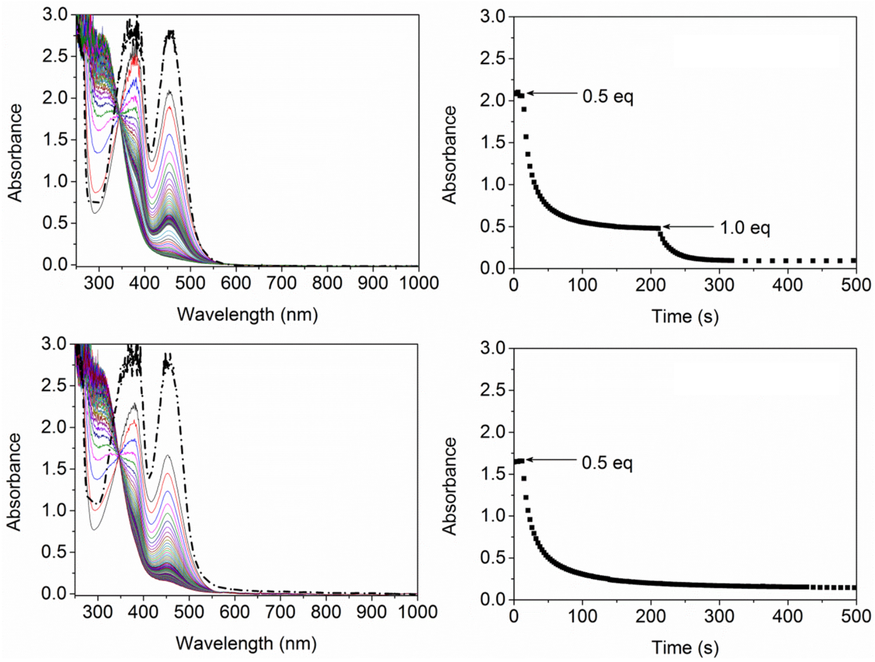


**Figure S16.** (left) UV/vis absorption spectrum of **1** in methanol with 5 vol% $H_2O$ (upper) and 10 vol% $H_2O$ (lower) at -30 °C with two-step addition of $H_2O_2$ (0.5 equiv. for each step). (right) The corresponding change in absorbance at 454 nm.

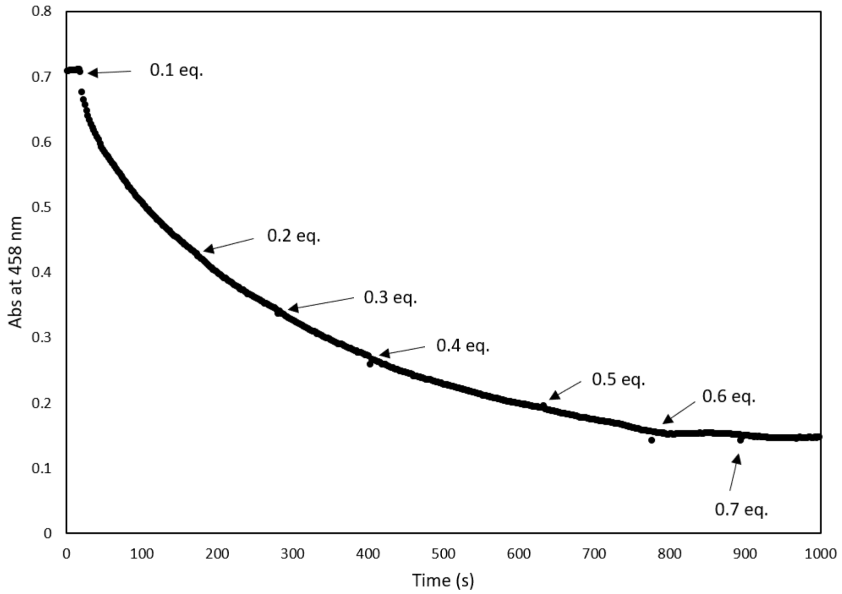


**Figure S17.** Absorbance changes at 458 nm over time during the stepwise addition of $H_2O_2$ to **2a** (0.2 mM) in $CH_3OH$ at -40 °C.

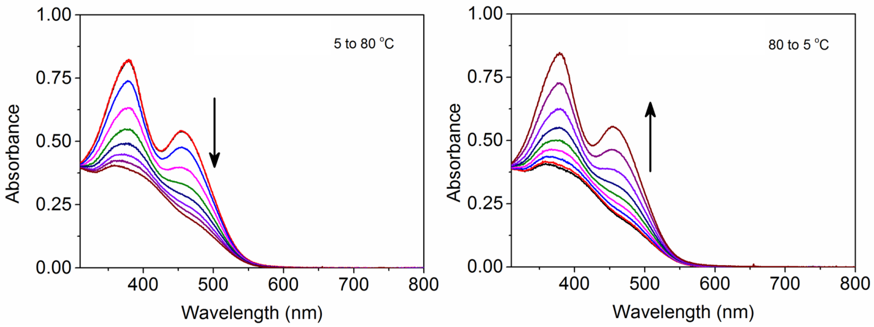


**Figure S18.** Temperature dependence of the UV/vis absorption spectrum of **1** (0.25 mM) (left) in $H_2O$ with increasing temperature (5 °C to 80 °C) and (right) with decreasing temperature (80 °C to 5 °C).

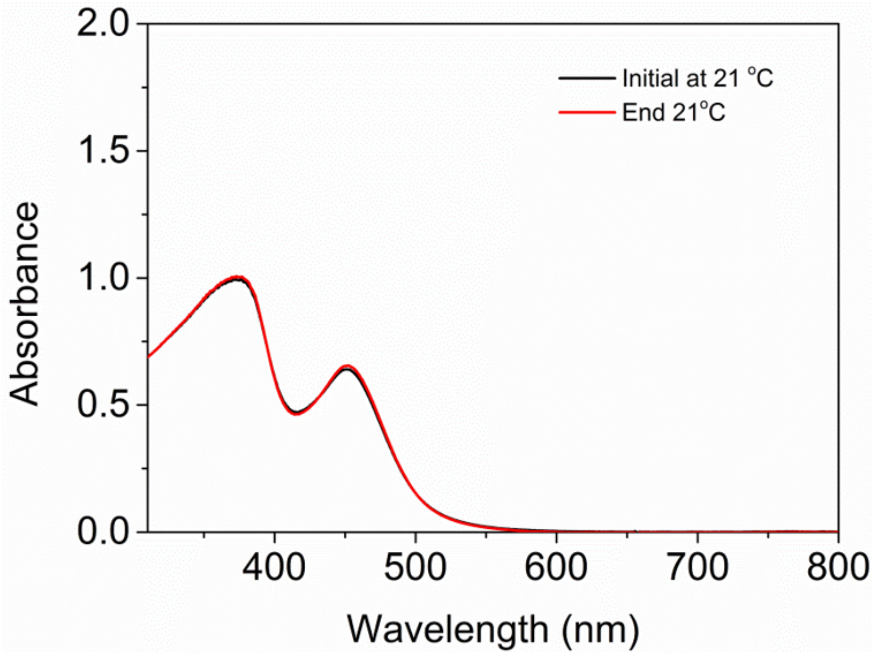


**Figure S19.** UV/vis absorption spectrum of **1** in deoxygenated methanol (black) and after increasing (21 °C to 40 °C), decreasing the temperature (40 °C to -30 °C) and then returning to 21 °C.

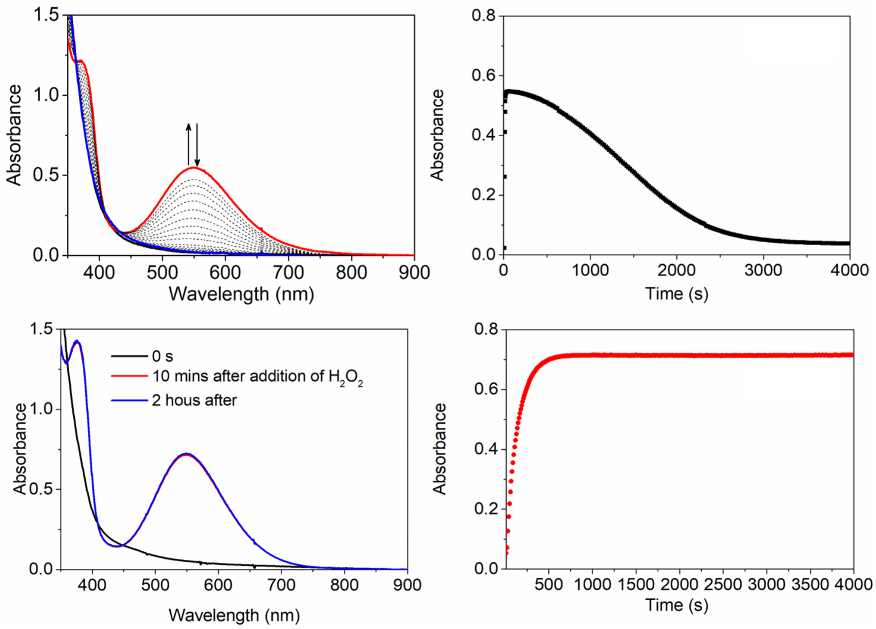


**Figure S20.** (left) UV/vis absorption spectrum of **1** (0.5 mM) in methanol before and after addition of 50 equiv. $H_2O_2$ at 21 °C (top) and - 30 °C (bottom), (right) absorbance at 550 nm over time at 21 °C (top) and - 30 °C (bottom).

# DFT Calculations

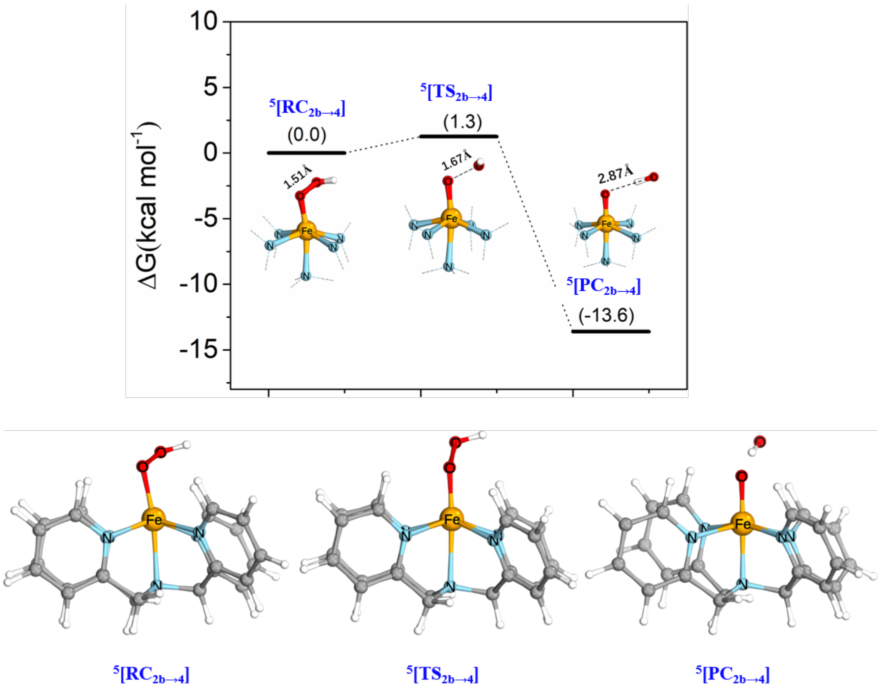


**Figure S21.** (top) Energy profile (in kcal mol$^{-1}$) of the heterolysis of **2b** at quintet state, as obtained by S12g/TZ2P//BP86-D3/TDZP level. (bottom) Corresponding structures are shown.

**Table S1.** Comparison of the rate of reactions.

| | in methanol (rt) | in methanol (-30 °C) | in $H_2O$ (rt) |
|---|---|---|---|
| $[(L)Fe^{IV}{=}O]$<br>Self-decay | $[(L)Fe^{IV}{=}O] \xrightarrow{k_{obs}}$<br>in $CH_3OH$: 1.0 mM, $1.8 \times 10^{-3}\ s^{-1}$; 0.5 mM, $3.0 \times 10^{-3}\ s^{-1}$<br>in $CD_3OD$: 0.5 mM, $6.48 \times 10^{-5}\ s^{-1}$ | $[(L)Fe^{IV}{=}O]$ + (0.5 mM) $\xrightarrow[-30\ ^{o}C]{k_{obs}}$<br>in $CH_3OH$ no reaction<br>in $CD_3OD$ no reaction | $[(L)Fe^{IV}{=}O]$ (0.5 mM) $\xrightarrow{k_{obs}}$<br>in $H_2O$ no reaction<br>in $D_2O$ no reaction |
| $[(L)Fe^{II}] + H_2O_2$ | $[(L)Fe^{II}\text{-}OCH_3] + H_2O_2$ (0.5 eq) $\xrightarrow{k_{obs}}$<br>in $CH_3OH$, fast, within mixing<br>in $CD_3OD$, fast, within mixing | $[(L)Fe^{II}\text{-}OCH_3]$ (0.5 mM) $+ H_2O_2$ (0.5 eq) $\xrightarrow[CH_3OH]{-30\ ^{o}C,\ k_{obs}}$<br>in $CH_3OH$ $7.0 \times 10^{-1}\ s^{-1}$<br>in $CD_3OD$ $5.0 \times 10^{-2}\ s^{-1}$ | $[(L)Fe^{II}\text{-}OCH_3]$ (0.5 mM) $+ H_2O_2$ (0.5 eq) $\xrightarrow[H_2O]{k_{obs}}$ fast, within mixing |
| $[(L)Fe^{IV}{=}O] + H_2O_2$ | $[(L)Fe^{IV}{=}O]$ (1.0 mM) $+ H_2O_2$ (1 eq) $\xrightarrow[CH_3OH]{k_{obs}}$ $1.0 \times 10^{-2}\ s^{-1}$<br>$[(L)Fe^{IV}{=}O]$ (1.0 mM) $+ D_2O_2$ (1 eq) $\xrightarrow[CD_3OD]{k_{obs}}$ $1.0 \times 10^{-3}\ s^{-1}$ | $[(L)Fe^{IV}{=}O]$ (0.5 mM) $+ H_2O_2$ (0.5 eq) $\xrightarrow[CH_3OH]{-30\ ^{o}C,\ k_{obs}}$ $3.0 \times 10^{-4}\ s^{-1}$ | |
| $[(L)Fe^{IV}{=}O] + [(L)Fe^{II}]$ | $[(L)Fe^{IV}{=}O]$ (1.0 mM) $+ [(L)Fe^{II}\text{-}OCH_3]$ (1 eq) $\xrightarrow{k_{obs}}$<br>in $CH_3OH$, $6.0 \times 10^{-3}\ s^{-1}$<br>in $CD_3OD$, $1.3 \times 10^{-3}\ s^{-1}$ | $[(L)Fe^{IV}{=}O]$ (1.0 mM) $+ [(L)Fe^{II}\text{-}OCH_3]$ (1 eq) $+ H_2O$ (0 vol%, 10 vol%, 50 vol%)<br>$\xrightarrow[CH_3OH]{-30\ ^{o}C,\ k_{obs}}$<br>0 vol%, no reaction<br>10 vol%, $3.9 \times 10^{-3}\ s^{-1}$<br>50 vol%, $4.3 \times 10^{-2}\ s^{-1}$ | $[(L)Fe^{IV}{=}O]$ (0.5 mM) $+ [(L)Fe^{II}\text{-}OH_2]$ (1 eq) $\xrightarrow[H_2O]{k_{obs}}$ $1.12\ s^{-1}$<br>$[(L)Fe^{IV}{=}O]$ (0.5 mM) $+ [(L)Fe^{II}\text{-}OH_2]$ (1 eq) $\xrightarrow[D_2O]{k_{obs}}$ $0.4\ s^{-1}$ |

**Table S2: Gibbs energy table for all complex and intermediates discussed (kcal mol$^{-1}$)**

| | geometry optimization/frequency calculation<br><br>BP86-$D_3$/TDZP | | Free energy corrections<br><br>(ΔG) | Single-point energy<br><br>S12g/ TZ2P | Final energy |
|---|---|---|---|---|---|
| | Electronic energy | Gibbs-free energy | | | |
| $[(N4Py)Fe^{II}(CH_3CN)]^{2+}$ ($^1$**1**) | -8263.88 | -8020.33 | 243.55 | -8482.34 | -8238.79 |
| $[(N4Py)Fe^{II}(CH_3CN)]^{2+}$ ($^3$**1**) | -8232.32 | -7991.79 | 240.47 | -8458.27 | -8217.80 |
| $[(N4Py)Fe^{II}(CH_3CN)]^{2+}$ ($^5$**1**) | -8227.22 | -7988.59 | 238.63 | -8461.69 | -8223.06 |
| $[(N4Py)Fe^{II}(OCH_3)]^{+}$ $^1$[**2a - H**$^+$] | -8116.73 | -7877.67 | 239.06 | -8312.87 | -8073.81 |
| $[(N4Py)Fe^{II}(OCH_3)]^{+}$ $^3$[**2a - H**$^+$] | -8098.72 | -7861.52 | 237.20 | -8299.06 | -8061.86 |
| $[(N4Py)Fe^{II}(OCH_3)]^{+}$ $^5$[**2a - H**$^+$] | -8101.21 | -7865.89 | 235.32 | -8313.27 | -8077.94 |
| $[(N4Py)Fe^{II}(HOCH_3)]^{2+}$ $^1$[**2a**] | -8109.71 | -7862.35 | 247.36 | -8312.41 | -8065.05 |
| $[(N4Py)Fe^{II}(HOCH_3)]^{2+}$ $^3$[**2a**] | -8087.29 | -7842.45 | 244.84 | -8298.41 | -8053.58 |
| $[(N4Py)Fe^{II}(HOCH_3)]^{2+}$ $^5$[**2a**] | -8083.46 | -7840.24 | 243.22 | -8302.42 | -8059.20 |
| $[(N4Py)Fe^{II}(OH)]^{+}$ $^1$[**2c - H**$^+$] | -7751.53 | -7528.18 | 223.34 | -7942.82 | -7719.47 |
| $[(N4Py)Fe^{II}(OH)]^{+}$ $^3$[**2c - H**$^+$] | -7729.50 | -7507.93 | 221.56 | -7920.70 | -7699.13 |
| $[(N4Py)Fe^{II}(OH)]^{+}$ $^5$[**2c - H**$^+$] | -7735.36 | -7516.68 | 218.67 | -7942.32 | -7723.65 |
| $[(N4Py)Fe^{II}(H_2O)]^{2+}$ $^1$[**2c**] | -7743.32 | -7512.69 | 230.63 | -7942.33 | -7711.71 |
| $[(N4Py)Fe^{II}(H_2O)]^{2+}$ $^3$[**2c**] | -7720.28 | -7492.19 | 228.08 | -7927.64 | -7699.56 |
| $[(N4Py)Fe^{II}(H_2O)]^{2+}$ $^5$[**2c**] | -7715.43 | -7488.78 | 226.65 | -7930.22 | -7703.57 |
| $[(N4Py)Fe^{II}(OOH)]^{+}$ $^1$[**2b**] | -7852.68 | -7628.61 | 224.07 | -8043.49 | -7819.42 |
| $[(N4Py)Fe^{II}(OOH)]^{+}$ $^3$[**2b**] | -7832.29 | -7610.43 | 221.86 | -8023.72 | -7801.86 |
| $[(N4Py)Fe^{II}(OOH)]^{+}$ $^5$[**2b**] | -7831.11 | -7610.38 | 220.73 | -8037.29 | -7816.56 |
| $[(N4Py)Fe^{II}(HOOH)]^{2+}$ $^1$[H**2b**] | -7832.62 | -7600.75 | 231.87 | -8032.60 | -7800.73 |
| $[(N4Py)Fe^{II}(HOOH)]^{2+}$ $^3$[H**2b**] | -7808.84 | -7580.14 | 228.70 | -8017.17 | -7788.47 |
| $[(N4Py)Fe^{II}(HOOH)]^{2+}$ $^5$[H**2b**] | -7802.89 | -7576.00 | 226.89 | -8019.10 | -7792.21 |
| $[(N4Py)Fe^{III}(OH)]^{2+}$ $^2$[**3c**] | -7645.4 | -7420.73 | 224.67 | -7846.61 | -7621.94 |
| $[(N4Py)Fe^{III}(OH)]^{2+}$ $^4$[**3c**] | -7621.1 | -7399.44 | 221.66 | -7831.28 | -7609.62 |
| $[(N4Py)Fe^{III}(OH)]^{2+}$ $^6$[**3c**] | -7619.68 | -7399.63 | 220.05 | -7838.02 | -7617.97 |
| $[(N4Py)Fe^{III}(H_2O)]^{2+}$ $^2$[H**3c**] | -7600.91 | -7370.51 | 230.4 | -7812.61 | -7582.21 |
| $[(N4Py)Fe^{III}(H_2O)]^{2+}$ $^4$[H**3c**] | -7582.04 | -7354.16 | 227.88 | -7803.15 | -7575.27 |
| $[(N4Py)Fe^{III}(H_2O)]^{2+}$ $^6$[H**3c**] | -7573.11 | -7346.38 | 226.73 | -7801.45 | -7574.72 |
| $[(N4Py)Fe^{IV}(O)]^{2+}$ $^1$[**4**] | -7533.46 | -7315.15 | 218.31 | -7712.60 | -7494.29 |
| $[(N4Py)Fe^{IV}(O)]^{2+}$ $^3$[**4**] | -7541.33 | -7322.98 | 218.35 | -7743.37 | -7525.02 |
| $[(N4Py)Fe^{IV}(O)]^{2+}$ $^5$[**4**] | -7517.93 | -7301.93 | 216 | -7729.15 | -7513.15 |

| | | | | | |
|---|---|---|---|---|---|
| $OH^-$ | -306.33 | -311.28 | -4.95 | -315.77 | -320.72 |
| $H_2O$ | -327.18 | -325.68 | 1.50 | -335.15 | -333.65 |
| $CH_3O^-$ | -667.08 | -658.51 | 8.57 | -681.77 | -673.20 |
| $CH_3OH$ | -688.86 | -672.29 | 16.57 | -704.17 | -687.60 |
| $OOH^-$ | -400.74 | -406.66 | -5.91 | -406.88 | -412.79 |
| HOOH | -415.59 | -413.66 | 1.93 | -425.44 | -423.51 |
| $OH^{\cdot}$ | -174.16 | -179.40 | -5.24 | -177.39 | -182.63 |
| $H_3O^+$ | -277.13 | -267.74 | 9.39 | -294.69 | -285.30 |
| $CH_3CN$ | -837.14 | -824.17 | 12.97 | -860.57 | -847.60 |

**Table S3.** Gibbs energy table for intermediate **2b** with considering the present of different adducts (kcal mol$^{-1}$), structures are shown in Table S4.

| | geometry optimization/frequency calculation BP86-D$_3$/TDZP | | | Free energy corrections (ΔG) | Single-point energy S12g/ TZ2P | |
|---|---|---|---|---|---|---|
| | | Electronic energy | Gibbs-free energy | | | |
| {[(N4Py)Fe$^{II}$(OOH)(**MeOH**)]$^{+}$}-**a** (with one $CH_3OH$ present as hydrogen bond donator to O31) | S=0 | -8551.19 | -8298.14 | 253.05 | -8754.09 | -8501.04 |
| | S=1 | -8571.65 | -8319.68 | 251.97 | -8782.12 | -8530.15 |
| | S=2 | -8528.21 | -8277.90 | 250.31 | -8747.42 | -8497.11 |
| {[(N4Py)Fe$^{II}$(OOH)(**MeOH**)]$^{+}$}-**b** (with one $CH_3OH$ present as hydrogen bond donator to O30) | S=0 | -8552.35 | -8298.75 | 253.60 | -8755.20 | -8501.60 |
| | S=1 | -8558.76 | -8306.76 | 252.00 | -8769.54 | -8517.54 |
| | S=2 | -8529.68 | -8279.51 | 250.17 | -8749.02 | -8498.85 |
| {[(N4Py)Fe$^{II}$(OOH)(**MeOH**)]$^{+}$}-**c** (with one $CH_3OH$ present as hydrogen bond acceptor ) | S=0 | -8547.53 | -8294.30 | 253.23 | -8751.90 | -8498.67 |
| | S=1 | -8542.46 | -8290.33 | 252.13 | -8749.25 | -8497.12 |
| | S=2 | -8531.86 | -8280.22 | 251.64 | -8744.30 | -8492.66 |
| {[(N4Py)Fe$^{II}$(OOH)(**MeOH**)$_2$]$^{+}$}-**a** (with two $CH_3OH$ present as hydrogen bond donator/acceptor to O31) | S=0 | -9249.10 | -8965.69 | 283.41 | -9464.98 | -9181.57 |
| | S=1 | -9270.41 | -8987.87 | 282.54 | -9491.91 | -9209.37 |
| | S=2 | -9234.48 | -8952.13 | 282.35 | -9456.56 | -9174.21 |
| {[(N4Py)Fe$^{II}$(OOH)(**MeOH**)$_2$]$^{+}$}-**b** (with two $CH_3OH$ present as hydrogen bond donator/acceptor to O30/O31) | S=0 | -9250.65 | -8966.99 | 283.66 | -9465.13 | -9181.47 |
| | S=1 | -9244.83 | -8963.24 | 281.59 | -9462.44 | -9180.85 |
| | S=2 | -9241.34 | -8959.54 | 281.80 | -9456.56 | -9174.76 |

| | | | | | | |
|---|---|---|---|---|---|---|
| {[(N4Py)$Fe^{II}$(OOH)(**$H_2O$**)]$^+$}-**a**<br>(with one $H_2O$ present as hydrogen donator to O31) | S=0 | -8187.65 | -7951.04 | 236.61 | -8383.78 | -8147.17 |
| | S=1 | -8206.98 | -7971.41 | 235.57 | -8409.49 | -8173.92 |
| | S=2 | -8165.14 | -7930.94 | 234.20 | -8375.41 | -8141.21 |
| {[(N4Py)$Fe^{II}$(OOH)(**$H_2O$**)]$^+$}-**b**<br>(with one $H_2O$ present as hydrogen acceptor) | S=0 | -8183.77 | -7946.91 | 236.86 | -8382.57 | -8145.71 |
| | S=1 | -8195.19 | -7958.20 | 236.99 | -8399.54 | -8162.55 |
| | S=2 | -8166.80 | -7931.72 | 235.08 | -8374.56 | -8139.48 |
| {[(N4Py)$Fe^{II}$(OOH)(**$H_2O$)$_2$**]$^+$}<br>(with two $H_2O$ present as hydrogen bond donator to O31 and O30) | S=0 | -8526.65 | -8276.13 | 250.52 | -8725.94 | -8475.42 |
| | S=1 | -8541.41 | -8293.24 | 248.17 | -8749.64 | -8501.47 |
| | S=2 | -8500.79 | -8253.85 | 246.94 | -8718.14 | -8471.20 |
| {[(N4Py)$Fe^{II}$(OOH)(**HOOH**)]$^+$}-**a**<br>(with one $H_2O_2$ present as hydro-gen bond donator to O30) | S=0 | -8283.96 | -8045.20 | 238.76 | -8478.31 | -8239.55 |
| | S=1 | -8288.88 | -8052.49 | 236.39 | -8512.62 | -8276.23 |
| | S=2 | -8260.01 | -8023.49 | 236.52 | -8470.09 | -8233.57 |

**Table S4.** Optimized structures for intermediate **2b** with considering the present of different adducts.

| {[(N4Py)Fe$^{II}$(OOH)(**MeOH**)]$^{+}$}**-a** (with one CH3OH present as hydrogen bond donator to O31) | | |
|---|---|---|
| S=0 | S=1 | S=2 |
| 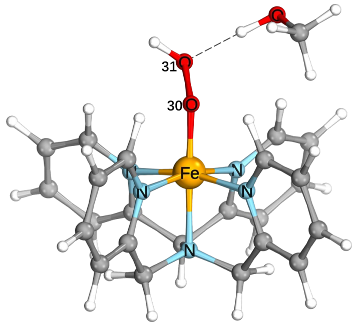 r(O-O) = 1.670 Å | 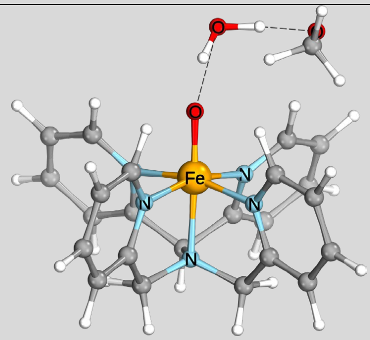 r(O-O) = 2.768 Å | 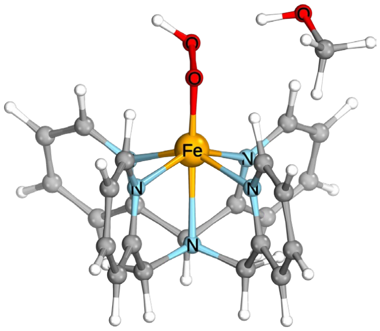 r(O-O) = 1.510 Å |
| {[(N4Py)Fe$^{II}$(OOH)(**MeOH**)]$^{+}$}**-b** (with one CH3OH present as hydrogen bond donator to O30) | | |
| S=1 | S=1 | |
| 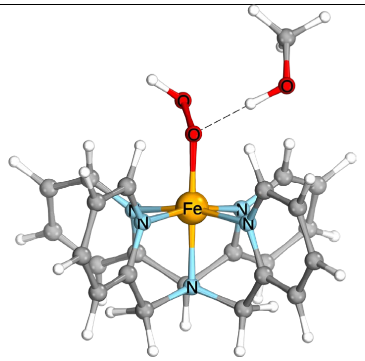 | 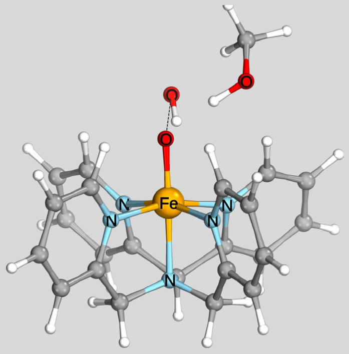 | 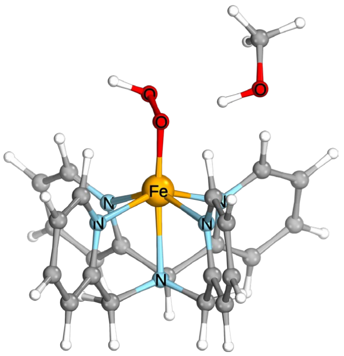 |

| | | |
|---|---|---|
| r(O-O) = 1.558 Å | r(O-O) = 2.910 Å | r(O-O) = 1.506 Å |
| {[(N4Py)$Fe^{II}$(OOH)(**MeOH**)]$^{+}$}**-c** (with one $CH_3OH$ present as hydrogen bond acceptor ) | | |
| S=0 | S=1 | S=2 |
| 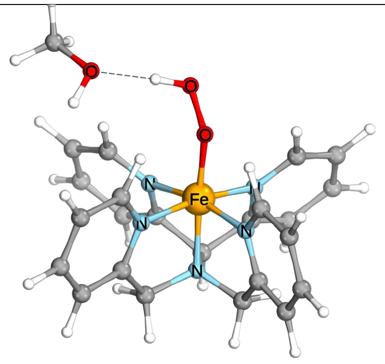<br>r(O-O) = 1.532 Å | 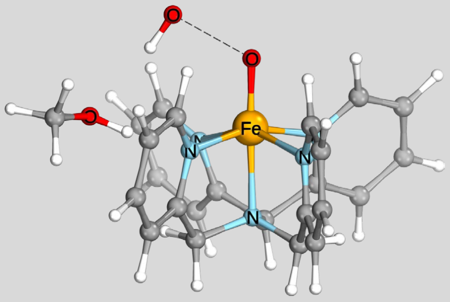<br>r(O-O) = 2.117Å | 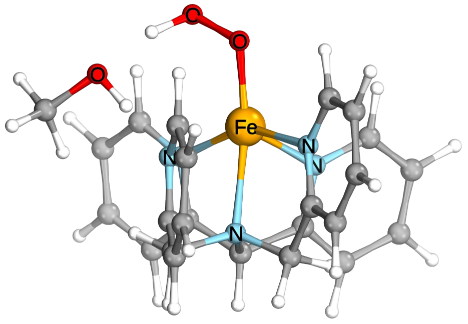<br>r(O-O) = 1.502Å |
| {[(N4Py)$Fe^{II}$(OOH)(**MeOH**)$_2$]$^{+}$}**-a** (with two $CH_3OH$ present as hydrogen bond donator/acceptor to O31) | | |
| S=0 | S=1 | S=2 |
| 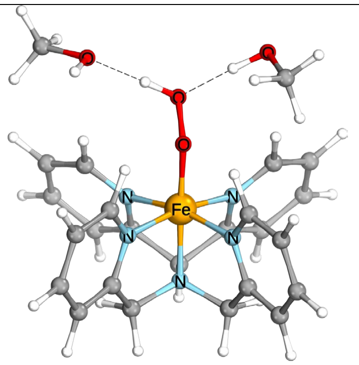<br>r(O-O) = 1.556 Å | 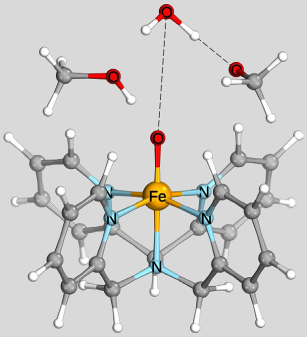<br>r(O-O) = 3.548 Å | 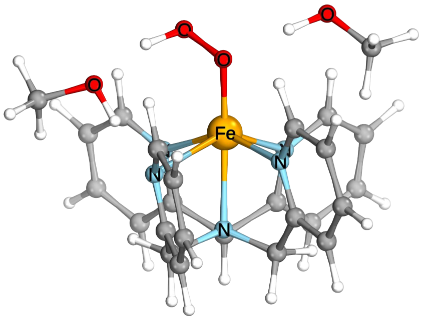<br>r(O-O) = 1.500 Å |

| {[(N4Py)$Fe^{II}$(OOH)(**MeOH)$_2$**]$^+$}-**b**(with two $CH_3OH$ present as hydrogen bond donator/acceptor to O30/O31) | | |
|---|---|---|
| S=0 | S=1 | S=2 |
| 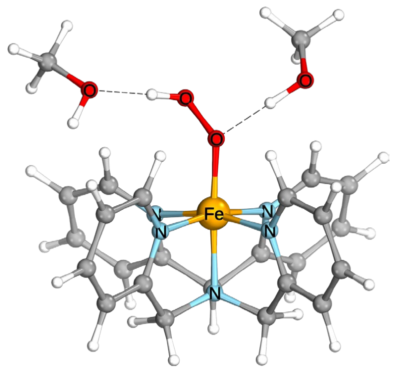<br>r(O-O) = 1.528 Å | 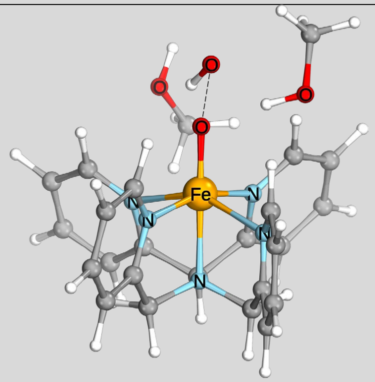<br>r(O-O) = 1.990 Å | 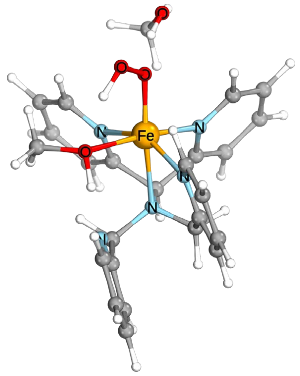<br>r(O-O) = 1.518 Å |

| {[(N4Py)$Fe^{II}$(OOH)(**$H_2$O**)]$^+$}-**b** (with one $H_2O$ present as hydrogen donator to O31) | | |
|---|---|---|
| S=0 | S=1 | S=2 |
| 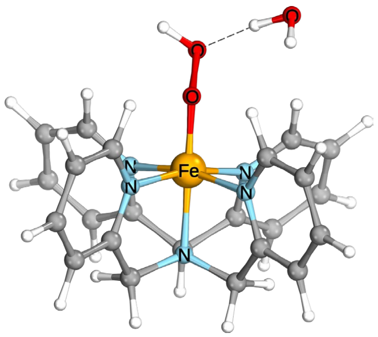<br>r(O-O) = 1.724 Å | 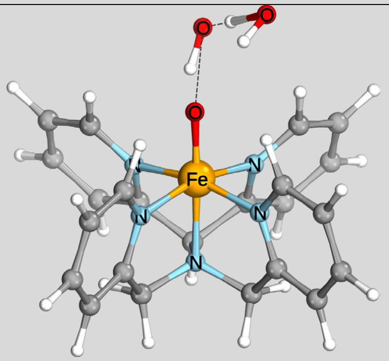<br>r(O-O) = 2.807 Å | 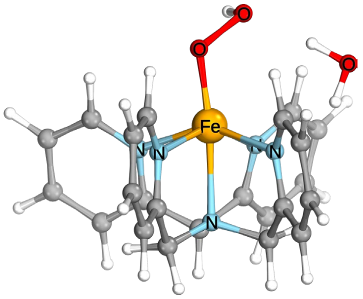<br>r(O-O) = 1.511 Å |

| {[(N4Py)$Fe^{II}$(OOH)(**$H_2O$**)]$^+$}**-a** (with one $H_2O$ present as hydrogen acceptor) | | |
|---|---|---|
| S=0 | S=1 | S=2 |
| 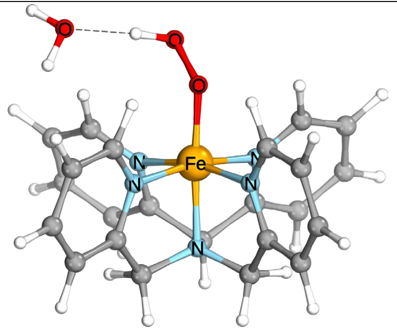 r(O-O) = 1.531 Å | 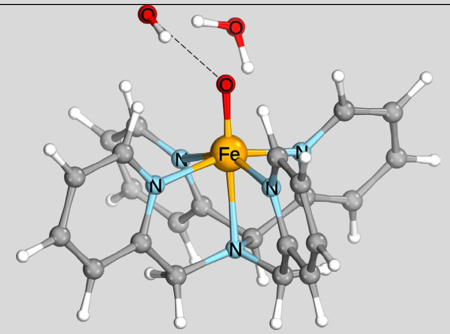 r(O-O) = 2.856 Å | 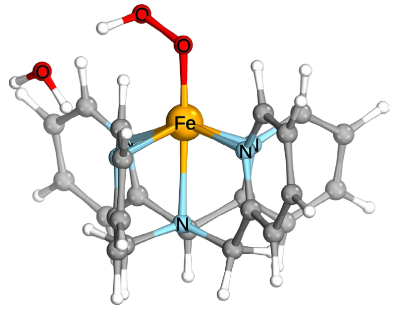 r(O-O) = 1.503 Å |
| {[(N4Py)$Fe^{II}$(OOH)(**$H_2O$**)$_2$]$^+$} (with two $H_2O$ present as hydrogen bond donator to O31 and O30) | | |
| S=0 | S=1 | S=2 |
| 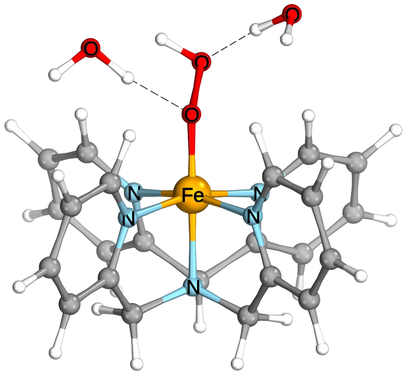 r(O-O) = 1.815 Å | 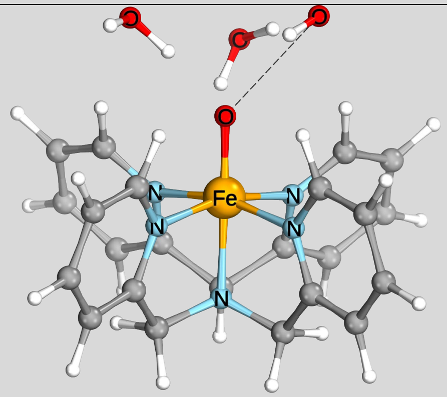 r(O-O) = 2.818 Å | 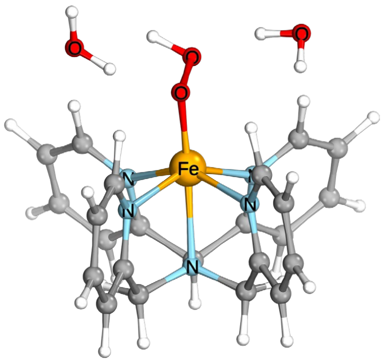 r(O-O) = 1.511 Å |

| {[(N4Py)$Fe^{II}$(OOH)(**HOOH**)]$^{+}$} (with one $H_2O_2$ present as hydrogen bond donator to O30) | | |
|---|---|---|
| S=0 | S=1 | S=2 |
| 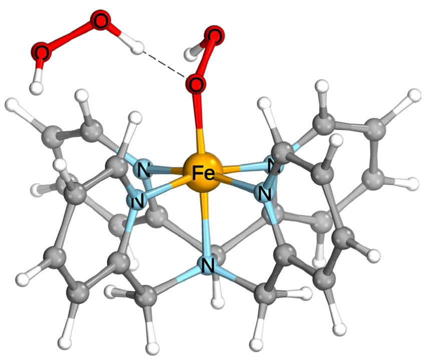 r(O-O) = 1.553 Å | 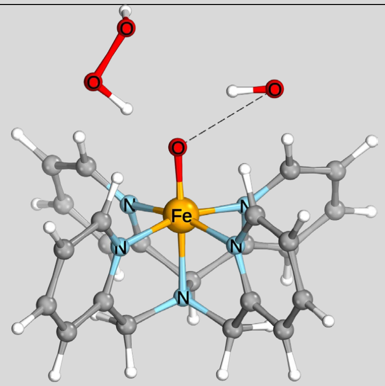 r(O-O) = 2.670 Å | 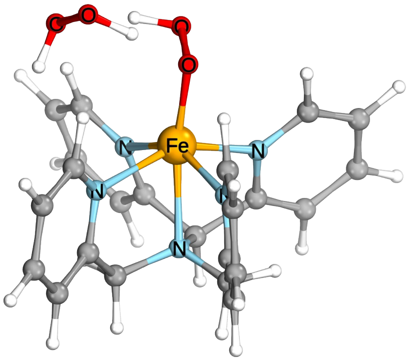 r(O-O) = 1.510 Å |

**Table S5.** Cartesian coordinates for all the optimized structures (*method described in the experimental section*).

| $^{1}$[(N4Py)Fe$^{II}$(CH$_3$CN)]$^{2+}$ | | | | $^{3}$[(N4Py)Fe$^{II}$(CH$_3$CN)]$^{2+}$ | | | |
|---|---|---|---|---|---|---|---|
| 56 | | | | 56 | | | |
| symmetry c1 | | | | symmetry c1 | | | |
| C | 1.638385000 | -2.377433000 | -1.047954000 | C | 1.854980000 | 2.343391000 | 0.888475000 |
| C | 2.642290000 | -3.297787000 | -0.769067000 | C | 2.916264000 | 3.165774000 | 0.525962000 |
| C | 3.400121000 | -3.155881000 | 0.392670000 | C | 3.605102000 | 2.902642000 | -0.657793000 |
| C | 3.129819000 | -2.093722000 | 1.258019000 | C | 3.213733000 | 1.824185000 | -1.453226000 |
| C | 2.117675000 | -1.214042000 | 0.917336000 | C | 2.150419000 | 1.046236000 | -1.025782000 |
| N | 1.388713000 | -1.345344000 | -0.223006000 | N | 1.490840000 | 1.300170000 | 0.126618000 |
| C | 1.678652000 | -0.009367000 | 1.713772000 | C | 1.607201000 | -0.166322000 | -1.753866000 |
| C | 2.122270000 | 1.204002000 | 0.933281000 | C | 1.976871000 | -1.369288000 | -0.904660000 |
| C | 3.137456000 | 2.075343000 | 1.285902000 | C | 2.910455000 | -2.326319000 | -1.265912000 |
| C | 3.411282000 | 3.148360000 | 0.435215000 | C | 3.155657000 | -3.387230000 | -0.392559000 |
| C | 2.653875000 | 3.308782000 | -0.724446000 | C | 2.452843000 | -3.451717000 | 0.809194000 |
| C | 1.646844000 | 2.395695000 | -1.016046000 | C | 1.531586000 | -2.457149000 | 1.114147000 |
| N | 1.393470000 | 1.353391000 | -0.205119000 | N | 1.301862000 | -1.427271000 | 0.278041000 |
| N | 0.160457000 | -0.005485000 | 1.610347000 | N | 0.115456000 | -0.067129000 | -1.721175000 |
| C | -0.470762000 | 1.236579000 | 2.162127000 | C | -0.598600000 | -1.260379000 | -2.210611000 |
| C | -0.476276000 | -1.251580000 | 2.146034000 | C | -0.478141000 | 1.209780000 | -2.171089000 |
| C | -1.557880000 | -1.699541000 | 1.207646000 | C | -1.504854000 | 1.715865000 | -1.182217000 |
| C | -1.550102000 | 1.701401000 | 1.229374000 | C | -1.716950000 | -1.639415000 | -1.267681000 |
| C | -2.614511000 | 2.493677000 | 1.636144000 | C | -2.851500000 | -2.321642000 | -1.691926000 |
| C | -3.531640000 | 2.938137000 | 0.684570000 | C | -3.812042000 | -2.691207000 | -0.752130000 |
| C | -3.354020000 | 2.569765000 | -0.647325000 | C | -3.608506000 | -2.367497000 | 0.588779000 |
| C | -2.277223000 | 1.761419000 | -0.987170000 | C | -2.456782000 | -1.676760000 | 0.942494000 |
| N | -1.388028000 | 1.334270000 | -0.068891000 | N | -1.532436000 | -1.322832000 | 0.031188000 |
| N | -1.393225000 | -1.318244000 | -0.086178000 | N | -1.342933000 | 1.364338000 | 0.123740000 |
| C | -2.282122000 | -1.733028000 | -1.010303000 | C | -2.201987000 | 1.845856000 | 1.045923000 |
| C | -3.361170000 | -2.542866000 | -0.681214000 | C | -3.250233000 | 2.692030000 | 0.711127000 |
| C | -3.542489000 | -2.924101000 | 0.646536000 | C | -3.430911000 | 3.045705000 | -0.623831000 |
| C | -2.625845000 | -2.492151000 | 1.604359000 | C | -2.545985000 | 2.546343000 | -1.578034000 |
| Fe | 0.003200000 | 0.007974000 | -0.346080000 | Fe | -0.017492000 | -0.015670000 | 0.462120000 |
| N | -0.036725000 | 0.016283000 | -2.199194000 | N | -0.061959000 | -0.020496000 | 2.483409000 |
| C | 0.017275000 | 0.010977000 | -3.362085000 | C | 0.237417000 | 0.165176000 | 3.592629000 |
| C | 0.087263000 | -0.004537000 | -4.799124000 | C | 0.609096000 | 0.398082000 | 4.959332000 |
| H | 1.010737000 | -2.446208000 | -1.934279000 | H | 1.272825000 | 2.505582000 | 1.795267000 |
| H | 2.820338000 | -4.120348000 | -1.460946000 | H | 3.190352000 | 4.004925000 | 1.164462000 |
| H | 4.186527000 | -3.871920000 | 0.631304000 | H | 4.434960000 | 3.538412000 | -0.966665000 |
| H | 3.683213000 | -1.957320000 | 2.186620000 | H | 3.715310000 | 1.596441000 | -2.393304000 |
| H | 2.040001000 | -0.016934000 | 2.751710000 | H | 2.019593000 | -0.245190000 | -2.771770000 |
| H | 3.690292000 | 1.924177000 | 2.212577000 | H | 3.423336000 | -2.248490000 | -2.224127000 |
| H | 4.200004000 | 3.858506000 | 0.683775000 | H | 3.878614000 | -4.159774000 | -0.654666000 |
| H | 2.834597000 | 4.140412000 | -1.404737000 | H | 2.610438000 | -4.268328000 | 1.512948000 |
| H | 1.019575000 | 2.478373000 | -1.901523000 | H | 0.954012000 | -2.460898000 | 2.036517000 |
| H | -0.845941000 | 1.068497000 | 3.179848000 | H | -0.978923000 | -1.110691000 | -3.232054000 |
| H | 0.297635000 | 2.022416000 | 2.216197000 | H | 0.105487000 | -2.106134000 | -2.246338000 |
| H | 0.288457000 | -2.041784000 | 2.188884000 | H | 0.315442000 | 1.968819000 | -2.246244000 |
| H | -0.849577000 | -1.095488000 | 3.166332000 | H | -0.923396000 | 1.111722000 | -3.172551000 |
| H | -2.720408000 | 2.755545000 | 2.688608000 | H | -2.977824000 | -2.555125000 | -2.749322000 |
| H | -4.377632000 | 3.557546000 | 0.982654000 | H | -4.713498000 | -3.218403000 | -1.064927000 |
| H | -4.047896000 | 2.891594000 | -1.423391000 | H | -4.336310000 | -2.632520000 | 1.355022000 |
| H | -2.107458000 | 1.429897000 | -2.008569000 | H | -2.253265000 | -1.380041000 | 1.971379000 |
| H | -2.110467000 | -1.390489000 | -2.027655000 | H | -2.029299000 | 1.522872000 | 2.070291000 |
| H | -4.054325000 | -2.854674000 | -1.461969000 | H | -3.918253000 | 3.053227000 | 1.492419000 |
| H | -4.390766000 | -3.544204000 | 0.936609000 | H | -4.254698000 | 3.694427000 | -0.922153000 |
| H | -2.734320000 | -2.764934000 | 2.653768000 | H | -2.659071000 | 2.796508000 | -2.632873000 |
| H | -0.303062000 | 0.938996000 | -5.205232000 | H | 0.226676000 | -0.420374000 | 5.584247000 |
| H | -0.509977000 | -0.838113000 | -5.193693000 | H | 0.182935000 | 1.348535000 | 5.307201000 |
| H | 1.131151000 | -0.128313000 | -5.118292000 | H | 1.703747000 | 0.432929000 | 5.043269000 |

| $^{5}[(N4Py)Fe^{II}(CH_3CN)]^{2+}$ | | | |
|---|---|---|---|
| 56 | | | |
| symmetry c1 | | | |
| C | -2.013900000 | -2.428573000 | 0.983124000 |
| C | -3.038524000 | -3.278972000 | 0.578836000 |
| C | -3.678379000 | -3.034276000 | -0.635302000 |
| C | -3.272253000 | -1.949684000 | -1.414245000 |
| C | -2.239186000 | -1.152476000 | -0.945392000 |
| N | -1.625795000 | -1.382316000 | 0.237174000 |
| C | -1.720952000 | 0.073824000 | -1.679107000 |
| C | -2.207167000 | 1.277813000 | -0.891564000 |
| C | -3.240533000 | 2.107416000 | -1.301049000 |
| C | -3.614000000 | 3.159392000 | -0.463766000 |
| C | -2.943393000 | 3.343116000 | 0.744917000 |
| C | -1.916531000 | 2.467653000 | 1.083592000 |
| N | -1.564238000 | 1.451776000 | 0.282278000 |
| N | -0.231217000 | 0.056166000 | -1.585931000 |
| C | 0.430574000 | 1.291667000 | -2.078151000 |
| C | 0.401523000 | -1.168105000 | -2.134185000 |
| C | 1.638653000 | -1.502076000 | -1.339282000 |
| C | 1.642635000 | 1.597968000 | -1.232760000 |
| C | 2.853717000 | 2.005923000 | -1.774346000 |
| C | 3.925006000 | 2.267395000 | -0.917938000 |
| C | 3.744876000 | 2.126952000 | 0.455797000 |
| C | 2.502357000 | 1.715579000 | 0.926523000 |
| N | 1.474367000 | 1.444467000 | 0.102410000 |
| N | 1.515039000 | -1.378375000 | 0.003611000 |
| C | 2.566375000 | -1.670868000 | 0.790356000 |
| C | 3.787563000 | -2.086328000 | 0.271585000 |
| C | 3.921538000 | -2.202312000 | -1.109530000 |
| C | 2.827701000 | -1.910457000 | -1.926347000 |
| Fe | 0.000157000 | -0.020844000 | 0.629498000 |
| N | 0.122826000 | -0.085803000 | 2.644485000 |
| C | 0.128231000 | -0.168962000 | 3.802853000 |
| C | 0.132399000 | -0.277755000 | 5.230762000 |
| H | -1.481687000 | -2.571724000 | 1.924036000 |
| H | -3.327070000 | -4.118590000 | 1.210579000 |
| H | -4.483120000 | -3.685221000 | -0.977919000 |
| H | -3.740374000 | -1.732138000 | -2.373942000 |
| H | -2.087617000 | 0.100858000 | -2.717482000 |
| H | -3.733041000 | 1.939901000 | -2.258615000 |
| H | -4.417642000 | 3.835084000 | -0.757840000 |
| H | -3.205598000 | 4.158302000 | 1.418780000 |
| H | -1.352190000 | 2.566487000 | 2.011248000 |
| H | 0.695518000 | 1.206370000 | -3.142750000 |
| H | -0.273610000 | 2.130471000 | -1.976126000 |
| H | -0.304375000 | -2.005018000 | -2.026436000 |
| H | 0.629701000 | -1.058116000 | -3.205246000 |
| H | 2.961876000 | 2.099580000 | -2.854925000 |
| H | 4.891263000 | 2.569119000 | -1.323336000 |
| H | 4.555104000 | 2.320698000 | 1.158083000 |
| H | 2.311823000 | 1.568944000 | 1.990291000 |
| H | 2.412614000 | -1.540706000 | 1.861937000 |
| H | 4.617988000 | -2.300208000 | 0.943751000 |
| H | 4.870549000 | -2.507786000 | -1.550960000 |
| H | 2.900451000 | -1.983490000 | -3.011399000 |
| H | 0.562177000 | 0.634150000 | 5.666807000 |
| H | 0.740454000 | -1.143586000 | 5.525141000 |
| H | -0.895346000 | -0.411179000 | 5.593318000 |

| $^{1}[(N4Py)Fe^{II}(OCH_3)]^{+}$ | | | |
|---|---|---|---|
| 55 | | | |
| symmetry c1 | | | |
| C | 1.494589000 | 2.413345000 | 1.174190000 |
| C | 2.468610000 | 3.370678000 | 0.922339000 |
| C | 3.290174000 | 3.240715000 | -0.200961000 |
| C | 3.100000000 | 2.154913000 | -1.060814000 |
| C | 2.110370000 | 1.237287000 | -0.752128000 |
| N | 1.332179000 | 1.348547000 | 0.361903000 |
| C | 1.718271000 | 0.031085000 | -1.571180000 |
| C | 2.116818000 | -1.193799000 | -0.785580000 |
| C | 3.104931000 | -2.098209000 | -1.135694000 |
| C | 3.286169000 | -3.229646000 | -0.336100000 |
| C | 2.449050000 | -3.418572000 | 0.765940000 |
| C | 1.482876000 | -2.466745000 | 1.064496000 |
| N | 1.333269000 | -1.349989000 | 0.321591000 |
| N | 0.188631000 | 0.020650000 | -1.539350000 |
| C | -0.435239000 | -1.221461000 | -2.092401000 |
| C | -0.440165000 | 1.263066000 | -2.082952000 |
| C | -1.543331000 | 1.713086000 | -1.161712000 |
| C | -1.522600000 | -1.700960000 | -1.164221000 |
| C | -2.566809000 | -2.523801000 | -1.566608000 |
| C | -3.477415000 | -2.979500000 | -0.611142000 |
| C | -3.314811000 | -2.594873000 | 0.720266000 |
| C | -2.260141000 | -1.754816000 | 1.056127000 |
| N | -1.384832000 | -1.317649000 | 0.131244000 |
| N | -1.398245000 | 1.330274000 | 0.132599000 |
| C | -2.287683000 | 1.743394000 | 1.054776000 |
| C | -3.363886000 | 2.555187000 | 0.716452000 |
| C | -3.533981000 | 2.936691000 | -0.614546000 |
| C | -2.607600000 | 2.507473000 | -1.567334000 |
| H | 2.139323000 | 0.045219000 | -2.587688000 |
| Fe | -0.020099000 | 0.006591000 | 0.428683000 |
| O | -0.314710000 | 0.082928000 | 2.340448000 |
| H | 0.815553000 | 2.450141000 | 2.025206000 |
| H | 2.579161000 | 4.215274000 | 1.602634000 |
| H | 4.055421000 | 3.986222000 | -0.417303000 |
| H | 3.694785000 | 2.033796000 | -1.966168000 |
| H | 3.701889000 | -1.932392000 | -2.032553000 |
| H | 4.051241000 | -3.965009000 | -0.585544000 |
| H | 2.540026000 | -4.303392000 | 1.396055000 |
| H | 0.796907000 | -2.569081000 | 1.901939000 |
| H | -0.810450000 | -1.060437000 | -3.112554000 |
| H | 0.336540000 | -2.004586000 | -2.146164000 |
| H | 0.324221000 | 2.055057000 | -2.110123000 |
| H | -0.797688000 | 1.114771000 | -3.111212000 |
| H | -2.663683000 | -2.803384000 | -2.615785000 |
| H | -4.306710000 | -3.623154000 | -0.906340000 |
| H | -4.004629000 | -2.932072000 | 1.494302000 |
| H | -2.060802000 | -1.385675000 | 2.063825000 |
| H | -2.079539000 | 1.383489000 | 2.063809000 |
| H | -4.065702000 | 2.870773000 | 1.488868000 |
| H | -4.380650000 | 3.556279000 | -0.911718000 |
| H | -2.707863000 | 2.785242000 | -2.616660000 |
| C | 0.779528000 | -0.127412000 | 3.193264000 |
| H | 0.849449000 | -1.171659000 | 3.573816000 |
| H | 1.756947000 | 0.091049000 | 2.713368000 |
| H | 0.712448000 | 0.529464000 | 4.086303000 |

| $^{3}[(N4Py)Fe^{II}(OCH_3)]^{+}$ | | | | $^{5}[(N4Py)Fe^{II}(OCH_3)]^{+}$ | | | |
|---|---|---|---|---|---|---|---|
| 55 | | | | 55 | | | |
| symmetry c1 | | | | symmetry c1 | | | |
| C | 2.303369000 | -2.246709000 | -0.965620000 | C | -1.875866000 | 2.480621000 | -1.273916000 |
| C | 3.405195000 | -2.950434000 | -0.486459000 | C | -2.911169000 | 3.344666000 | -0.933902000 |
| C | 3.968604000 | -2.573218000 | 0.733674000 | C | -3.565816000 | 3.167266000 | 0.286441000 |
| C | 3.418191000 | -1.499718000 | 1.437371000 | C | -3.166881000 | 2.124501000 | 1.124464000 |
| C | 2.326627000 | -0.845908000 | 0.881720000 | C | -2.129598000 | 1.301401000 | 0.709197000 |
| N | 1.779735000 | -1.210746000 | -0.295554000 | N | -1.492499000 | 1.472992000 | -0.471276000 |
| C | 1.651881000 | 0.363840000 | 1.497963000 | C | -1.653280000 | 0.090415000 | 1.496276000 |
| C | 2.022766000 | 1.558131000 | 0.643416000 | C | -2.218208000 | -1.114736000 | 0.759568000 |
| C | 2.946263000 | 2.524162000 | 1.008235000 | C | -3.300797000 | -1.852487000 | 1.217488000 |
| C | 3.202929000 | 3.577100000 | 0.128804000 | C | -3.769567000 | -2.905927000 | 0.430545000 |
| C | 2.510934000 | 3.624608000 | -1.084023000 | C | -3.135778000 | -3.179660000 | -0.782886000 |
| C | 1.600099000 | 2.623844000 | -1.389661000 | C | -2.053355000 | -2.395627000 | -1.168191000 |
| N | 1.361764000 | 1.586238000 | -0.555644000 | N | -1.602316000 | -1.378064000 | -0.415274000 |
| N | 0.157711000 | 0.201310000 | 1.322505000 | N | -0.174764000 | 0.030915000 | 1.400836000 |
| C | -0.591043000 | 1.370048000 | 1.891009000 | C | 0.432859000 | -1.210513000 | 1.921777000 |
| C | -0.412289000 | -1.087485000 | 1.824742000 | C | 0.532209000 | 1.237374000 | 1.877761000 |
| C | -1.491164000 | -1.560595000 | 0.882905000 | C | 1.769910000 | 1.471084000 | 1.042742000 |
| C | -1.945061000 | 1.412395000 | 1.237518000 | C | 1.633411000 | -1.592066000 | 1.086512000 |
| C | -3.123746000 | 1.134619000 | 1.919527000 | C | 2.813345000 | -2.068024000 | 1.644557000 |
| C | -4.310892000 | 1.032382000 | 1.190132000 | C | 3.871142000 | -2.416489000 | 0.803065000 |
| C | -4.270381000 | 1.228462000 | -0.188027000 | C | 3.706359000 | -2.284233000 | -0.575636000 |
| C | -3.046262000 | 1.521605000 | -0.788270000 | C | 2.499905000 | -1.792994000 | -1.060222000 |
| N | -1.896329000 | 1.603978000 | -0.099968000 | N | 1.481127000 | -1.445772000 | -0.250655000 |
| N | -1.335208000 | -1.179097000 | -0.416331000 | N | 1.603675000 | 1.311333000 | -0.291566000 |
| C | -2.232597000 | -1.601821000 | -1.332929000 | C | 2.655197000 | 1.522966000 | -1.105416000 |
| C | -3.316502000 | -2.398184000 | -0.991965000 | C | 3.910595000 | 1.880270000 | -0.627050000 |
| C | -3.491339000 | -2.773026000 | 0.339901000 | C | 4.088012000 | 2.023577000 | 0.748898000 |
| C | -2.558110000 | -2.348585000 | 1.287545000 | C | 2.995515000 | 1.821325000 | 1.594531000 |
| H | 1.942381000 | 0.501557000 | 2.550657000 | H | -2.025025000 | 0.125476000 | 2.534523000 |
| Fe | 0.062970000 | 0.141845000 | -0.737782000 | Fe | 0.015327000 | -0.011512000 | -0.909678000 |
| O | -0.040160000 | -0.053529000 | -2.563341000 | O | 0.123648000 | -0.014127000 | -2.770686000 |
| H | 1.814462000 | -2.501967000 | -1.906340000 | H | -1.322402000 | 2.577372000 | -2.208525000 |
| H | 3.810067000 | -3.784218000 | -1.060187000 | H | -3.192442000 | 4.149004000 | -1.613857000 |
| H | 4.824621000 | -3.114208000 | 1.138199000 | H | -4.374333000 | 3.835218000 | 0.585050000 |
| H | 3.823978000 | -1.181625000 | 2.397769000 | H | -3.648235000 | 1.955105000 | 2.087966000 |
| H | 3.446879000 | 2.456836000 | 1.974197000 | H | -3.761878000 | -1.611297000 | 2.175434000 |
| H | 3.918482000 | 4.356037000 | 0.392673000 | H | -4.614000000 | -3.509491000 | 0.764203000 |
| H | 2.669769000 | 4.438131000 | -1.791720000 | H | -3.469361000 | -3.996960000 | -1.422354000 |
| H | 1.027232000 | 2.623637000 | -2.314190000 | H | -1.514819000 | -2.572571000 | -2.099685000 |
| H | -0.651728000 | 1.285659000 | 2.986489000 | H | 0.704240000 | -1.115597000 | 2.985913000 |
| H | -0.033443000 | 2.281463000 | 1.634945000 | H | -0.304178000 | -2.023353000 | 1.839727000 |
| H | 0.387083000 | -1.842936000 | 1.837495000 | H | -0.130927000 | 2.105823000 | 1.748551000 |
| H | -0.787552000 | -0.981680000 | 2.853303000 | H | 0.782888000 | 1.166081000 | 2.948802000 |
| H | -3.107818000 | 0.965470000 | 2.996728000 | H | 2.906464000 | -2.150604000 | 2.727963000 |
| H | -5.247354000 | 0.783925000 | 1.690759000 | H | 4.812469000 | -2.776544000 | 1.219739000 |
| H | -5.170605000 | 1.144271000 | -0.797414000 | H | 4.507000000 | -2.540473000 | -1.269704000 |
| H | -2.967259000 | 1.660819000 | -1.868207000 | H | 2.324418000 | -1.634882000 | -2.125721000 |
| H | -2.040690000 | -1.253042000 | -2.347748000 | H | 2.463714000 | 1.361566000 | -2.167782000 |
| H | -4.024599000 | -2.701928000 | -1.763355000 | H | 4.736470000 | 2.026109000 | -1.323389000 |
| H | -4.345637000 | -3.379728000 | 0.641156000 | H | 5.064790000 | 2.279350000 | 1.160723000 |
| H | -2.664615000 | -2.612603000 | 2.339781000 | H | 3.096387000 | 1.917786000 | 2.676067000 |
| C | 1.127973000 | 0.081971000 | -3.357428000 | C | -1.052128000 | -0.056243000 | -3.560032000 |
| H | 1.071386000 | 0.971185000 | -4.014301000 | H | -1.110401000 | -1.002289000 | -4.131550000 |
| H | 2.048433000 | 0.168900000 | -2.750693000 | H | -1.976013000 | 0.018850000 | -2.953320000 |
| H | 1.243245000 | -0.806771000 | -4.006126000 | H | -1.067143000 | 0.777157000 | -4.288155000 |

$^{1}[(N4Py)Fe^{II}(OH_2)]^{2+}$

53
symmetry c1

| | | | |
|---|---|---|---|
| C | -1.665910000 | -2.386847000 | 1.275692000 |
| C | -2.686505000 | -3.292666000 | 1.011044000 |
| C | -3.459959000 | -3.138430000 | -0.139020000 |
| C | -3.185371000 | -2.079393000 | -1.007163000 |
| C | -2.157602000 | -1.213518000 | -0.679628000 |
| N | -1.413278000 | -1.351739000 | 0.452895000 |
| C | -1.709951000 | -0.019132000 | -1.485278000 |
| C | -2.133262000 | 1.203911000 | -0.710568000 |
| C | -3.129971000 | 2.094922000 | -1.065895000 |
| C | -3.371683000 | 3.185203000 | -0.227005000 |
| C | -2.594323000 | 3.347724000 | 0.919471000 |
| C | -1.605372000 | 2.416247000 | 1.213237000 |
| N | -1.391611000 | 1.347785000 | 0.422145000 |
| N | -0.185568000 | -0.032637000 | -1.386336000 |
| C | 0.462948000 | 1.198359000 | -1.949373000 |
| C | 0.431307000 | -1.294231000 | -1.913586000 |
| C | 1.526738000 | -1.728728000 | -0.984793000 |
| C | 1.528223000 | 1.679047000 | -1.005665000 |
| C | 2.589361000 | 2.481111000 | -1.401203000 |
| C | 3.486665000 | 2.941352000 | -0.437343000 |
| C | 3.292208000 | 2.581945000 | 0.894957000 |
| C | 2.219741000 | 1.762578000 | 1.223437000 |
| N | 1.353533000 | 1.317409000 | 0.292440000 |
| N | 1.378602000 | -1.323439000 | 0.305773000 |
| C | 2.304554000 | -1.694227000 | 1.211797000 |
| C | 3.389597000 | -2.494974000 | 0.880153000 |
| C | 3.542607000 | -2.910768000 | -0.440462000 |
| C | 2.597892000 | -2.513518000 | -1.386623000 |
| Fe | -0.025721000 | -0.011180000 | 0.541338000 |
| O | 0.006464000 | 0.085075000 | 2.584038000 |
| H | -1.026259000 | -2.476419000 | 2.151676000 |
| H | -2.864198000 | -4.114584000 | 1.703743000 |
| H | -4.259445000 | -3.843115000 | -0.367334000 |
| H | -3.748050000 | -1.935535000 | -1.929133000 |
| H | -3.692544000 | 1.946756000 | -1.987235000 |
| H | -4.146916000 | 3.909404000 | -0.477159000 |
| H | -2.743725000 | 4.196783000 | 1.585682000 |
| H | -0.955912000 | 2.509778000 | 2.082475000 |
| H | 0.856676000 | 1.008601000 | -2.956033000 |
| H | -0.301899000 | 1.984850000 | -2.033437000 |
| H | -0.341415000 | -2.077422000 | -1.928994000 |
| H | 0.788477000 | -1.157780000 | -2.942447000 |
| H | 2.708691000 | 2.739913000 | -2.453029000 |
| H | 4.330165000 | 3.568408000 | -0.726481000 |
| H | 3.968791000 | 2.920557000 | 1.678988000 |
| H | 2.028315000 | 1.440827000 | 2.245132000 |
| H | 2.179148000 | -1.319072000 | 2.224563000 |
| H | 4.109023000 | -2.768867000 | 1.651055000 |
| H | 4.393231000 | -3.525220000 | -0.735219000 |
| H | 2.690495000 | -2.804161000 | -2.432726000 |
| H | 0.409648000 | -0.665540000 | 3.077424000 |
| H | -0.912561000 | 0.131833000 | 2.939753000 |
| H | -2.074399000 | -0.028828000 | -2.522143000 |

$^{3}[(N4Py)Fe^{II}(OH_2)]^{2+}$

53
symmetry c1

| | | | |
|---|---|---|---|
| C | -1.910466000 | -2.309091000 | 1.208607000 |
| C | -2.983503000 | -3.130348000 | 0.880695000 |
| C | -3.684836000 | -2.893447000 | -0.300849000 |
| C | -3.294206000 | -1.839283000 | -1.128274000 |
| C | -2.220305000 | -1.058024000 | -0.735810000 |
| N | -1.546363000 | -1.286805000 | 0.416804000 |
| C | -1.679045000 | 0.127544000 | -1.508978000 |
| C | -2.007661000 | 1.358272000 | -0.681971000 |
| C | -2.919113000 | 2.332882000 | -1.052003000 |
| C | -3.110584000 | 3.427374000 | -0.206584000 |
| C | -2.373013000 | 3.510742000 | 0.973115000 |
| C | -1.474874000 | 2.498255000 | 1.287314000 |
| N | -1.305925000 | 1.429660000 | 0.484651000 |
| N | -0.187770000 | 0.000209000 | -1.516861000 |
| C | 0.554631000 | 1.175857000 | -2.006806000 |
| C | 0.373227000 | -1.294440000 | -1.951128000 |
| C | 1.412039000 | -1.798464000 | -0.971319000 |
| C | 1.653378000 | 1.574396000 | -1.044636000 |
| C | 2.775451000 | 2.284175000 | -1.455495000 |
| C | 3.715719000 | 2.683424000 | -0.507278000 |
| C | 3.506823000 | 2.361544000 | 0.833095000 |
| C | 2.372431000 | 1.640655000 | 1.179276000 |
| N | 1.468461000 | 1.256099000 | 0.257156000 |
| N | 1.286116000 | -1.410472000 | 0.328166000 |
| C | 2.166398000 | -1.873697000 | 1.238502000 |
| C | 3.194412000 | -2.744429000 | 0.904421000 |
| C | 3.332587000 | -3.141074000 | -0.423511000 |
| C | 2.430945000 | -2.655047000 | -1.369061000 |
| Fe | -0.017590000 | -0.007444000 | 0.651678000 |
| O | -0.056820000 | 0.027275000 | 2.844345000 |
| H | -1.320130000 | -2.454482000 | 2.112113000 |
| H | -3.256483000 | -3.949191000 | 1.545494000 |
| H | -4.523126000 | -3.530338000 | -0.583362000 |
| H | -3.804784000 | -1.632282000 | -2.068326000 |
| H | -3.454548000 | 2.244206000 | -1.996841000 |
| H | -3.815170000 | 4.214067000 | -0.476534000 |
| H | -2.483426000 | 4.357159000 | 1.650145000 |
| H | -0.862810000 | 2.524528000 | 2.187498000 |
| H | 0.966110000 | 0.998749000 | -3.011637000 |
| H | -0.138364000 | 2.027656000 | -2.087965000 |
| H | -0.435407000 | -2.039993000 | -1.995053000 |
| H | 0.801395000 | -1.226648000 | -2.962757000 |
| H | 2.906868000 | 2.518373000 | -2.512029000 |
| H | 4.605297000 | 3.234070000 | -0.813131000 |
| H | 4.217356000 | 2.651864000 | 1.606262000 |
| H | 2.162732000 | 1.347782000 | 2.207458000 |
| H | 2.041720000 | -1.514930000 | 2.257188000 |
| H | 3.879716000 | -3.088747000 | 1.678272000 |
| H | 4.138565000 | -3.811046000 | -0.723511000 |
| H | 2.515565000 | -2.933877000 | -2.419337000 |
| H | 0.326994000 | -0.660407000 | 3.434708000 |
| H | -0.946235000 | 0.210655000 | 3.226358000 |
| H | -2.121056000 | 0.187461000 | -2.515852000 |

| $^{5}[(N4Py)Fe^{II}(OH_2)]^{2+}$ | | | |
|---|---|---|---|
| 53 | | | |
| symmetry c1 | | | |
| C | -2.008039000 | 2.468677000 | -1.197796000 |
| C | -3.029185000 | 3.317632000 | -0.782882000 |
| C | -3.645274000 | 3.083353000 | 0.445469000 |
| C | -3.219281000 | 2.008950000 | 1.227588000 |
| C | -2.196886000 | 1.206974000 | 0.744385000 |
| N | -1.606227000 | 1.425961000 | -0.452491000 |
| C | -1.665180000 | -0.013220000 | 1.476711000 |
| C | -2.192300000 | -1.222323000 | 0.724022000 |
| C | -3.224580000 | -2.026168000 | 1.182979000 |
| C | -3.645966000 | -3.085639000 | 0.378643000 |
| C | -3.017154000 | -3.303962000 | -0.846408000 |
| C | -1.985349000 | -2.455380000 | -1.234496000 |
| N | -1.588549000 | -1.427331000 | -0.467789000 |
| N | -0.177686000 | -0.011063000 | 1.333334000 |
| C | 0.488176000 | -1.246800000 | 1.818644000 |
| C | 0.487418000 | 1.216696000 | 1.837410000 |
| C | 1.685426000 | 1.545996000 | 0.981341000 |
| C | 1.680391000 | -1.565630000 | 0.950848000 |
| C | 2.889354000 | -2.003901000 | 1.473597000 |
| C | 3.943199000 | -2.287363000 | 0.603712000 |
| C | 3.747502000 | -2.135418000 | -0.766649000 |
| C | 2.509462000 | -1.692127000 | -1.217704000 |
| N | 1.495250000 | -1.402311000 | -0.382059000 |
| N | 1.508742000 | 1.394386000 | -0.353306000 |
| C | 2.521189000 | 1.699672000 | -1.185397000 |
| C | 3.752371000 | 2.152268000 | -0.724787000 |
| C | 3.941771000 | 2.291863000 | 0.647868000 |
| C | 2.888648000 | 1.989273000 | 1.512462000 |
| Fe | -0.032216000 | 0.019364000 | -0.861947000 |
| O | -0.079367000 | 0.178364000 | -2.957438000 |
| H | -1.486830000 | 2.614918000 | -2.144151000 |
| H | -3.329562000 | 4.152024000 | -1.415961000 |
| H | -4.444455000 | 3.736032000 | 0.797472000 |
| H | -3.664881000 | 1.801711000 | 2.200099000 |
| H | -3.681129000 | -1.831653000 | 2.153087000 |
| H | -4.452028000 | -3.740107000 | 0.711152000 |
| H | -3.314501000 | -4.127151000 | -1.495388000 |
| H | -1.452130000 | -2.589816000 | -2.175948000 |
| H | 0.775170000 | -1.153491000 | 2.876577000 |
| H | -0.223084000 | -2.081820000 | 1.737719000 |
| H | -0.221539000 | 2.054245000 | 1.760487000 |
| H | 0.766801000 | 1.111138000 | 2.896294000 |
| H | 3.007988000 | -2.106402000 | 2.552196000 |
| H | 4.906711000 | -2.615364000 | 0.994722000 |
| H | 4.541825000 | -2.344779000 | -1.482318000 |
| H | 2.317698000 | -1.545736000 | -2.280664000 |
| H | 2.327792000 | 1.557693000 | -2.249132000 |
| H | 4.547459000 | 2.377341000 | -1.434876000 |
| H | 4.900588000 | 2.626066000 | 1.045172000 |
| H | 3.003036000 | 2.083177000 | 2.592267000 |
| H | 0.543579000 | -0.290129000 | -3.560564000 |
| H | -0.965917000 | 0.083869000 | -3.379813000 |
| H | -1.992197000 | -0.022418000 | 2.528167000 |

| $^{1}[(N4Py)Fe^{II}(OOH)]^{+}$ | | | |
|---|---|---|---|
| 53 | | | |
| symmetry c1 | | | |
| C | 1.569896000 | -2.409686000 | -1.121273000 |
| C | 2.530875000 | -3.357257000 | -0.789228000 |
| C | 3.283895000 | -3.198225000 | 0.375683000 |
| C | 3.038615000 | -2.095180000 | 1.197105000 |
| C | 2.060554000 | -1.194367000 | 0.811117000 |
| N | 1.347154000 | -1.334534000 | -0.340359000 |
| C | 1.622722000 | 0.026882000 | 1.583646000 |
| C | 2.057833000 | 1.235500000 | 0.787427000 |
| C | 3.035588000 | 2.146264000 | 1.146923000 |
| C | 3.274341000 | 3.234758000 | 0.302266000 |
| C | 2.508011000 | 3.377169000 | -0.856634000 |
| C | 1.537795000 | 2.429546000 | -1.157028000 |
| N | 1.332825000 | 1.359003000 | -0.361498000 |
| N | 0.101841000 | 0.020482000 | 1.484981000 |
| C | -0.545644000 | 1.262213000 | 2.003176000 |
| C | -0.544004000 | -1.221422000 | 2.007649000 |
| C | -1.590523000 | -1.698022000 | 1.034685000 |
| C | -1.609925000 | 1.713656000 | 1.040375000 |
| C | -2.688190000 | 2.504315000 | 1.414718000 |
| C | -3.585027000 | 2.936383000 | 0.436717000 |
| C | -3.371624000 | 2.560577000 | -0.888941000 |
| C | -2.282432000 | 1.753956000 | -1.195084000 |
| N | -1.417422000 | 1.337240000 | -0.250556000 |
| N | -1.397570000 | -1.322161000 | -0.255854000 |
| C | -2.240682000 | -1.761624000 | -1.208889000 |
| C | -3.312273000 | -2.594330000 | -0.910276000 |
| C | -3.529168000 | -2.970174000 | 0.415130000 |
| C | -2.653722000 | -2.512940000 | 1.401222000 |
| Fe | -0.016189000 | 0.014127000 | -0.503161000 |
| O | -0.225468000 | 0.088552000 | -2.362135000 |
| O | 1.051787000 | -0.285943000 | -3.163939000 |
| H | 0.961092000 | -2.465229000 | -2.020187000 |
| H | 2.685131000 | -4.214615000 | -1.444408000 |
| H | 4.041336000 | -3.932427000 | 0.651254000 |
| H | 3.580695000 | -1.945524000 | 2.130729000 |
| H | 3.584973000 | 2.017351000 | 2.079440000 |
| H | 4.034566000 | 3.972905000 | 0.557982000 |
| H | 2.655218000 | 4.224144000 | -1.526635000 |
| H | 0.892724000 | 2.485215000 | -2.034180000 |
| H | -0.945144000 | 1.113607000 | 3.015877000 |
| H | 0.217204000 | 2.053654000 | 2.062980000 |
| H | 0.223804000 | -2.005502000 | 2.091734000 |
| H | -0.962112000 | -1.062771000 | 3.011299000 |
| H | -2.821674000 | 2.775759000 | 2.461984000 |
| H | -4.441702000 | 3.553152000 | 0.709719000 |
| H | -4.047398000 | 2.877094000 | -1.683323000 |
| H | -2.059129000 | 1.412088000 | -2.205982000 |
| H | -2.010011000 | -1.407940000 | -2.214298000 |
| H | -3.972487000 | -2.931482000 | -1.709324000 |
| H | -4.372955000 | -3.607198000 | 0.682002000 |
| H | -2.791507000 | -2.784885000 | 2.447822000 |
| H | 1.704011000 | 0.318733000 | -2.738798000 |
| H | 1.995532000 | 0.037731000 | 2.618720000 |

| $^{3}[(N4Py)Fe^{II}(OOH)]^{+}$ | | | | $^{5}[(N4Py)Fe^{II}(OOH)]^{+}$ | | | |
|---|---|---|---|---|---|---|---|
| 53 | | | | 53 | | | |
| symmetry c1 | | | | symmetry c1 | | | |
| C | 1.534638000 | 2.651154000 | 1.311542000 | C | -1.954631000 | -2.575434000 | 0.943077000 |
| C | 2.479115000 | 3.623643000 | 1.006772000 | C | -2.932886000 | -3.435303000 | 0.453863000 |
| C | 3.178849000 | 3.545168000 | -0.196382000 | C | -3.560793000 | -3.129225000 | -0.754286000 |
| C | 2.923023000 | 2.483294000 | -1.066037000 | C | -3.186957000 | -1.970178000 | -1.434812000 |
| C | 1.972063000 | 1.545097000 | -0.702558000 | C | -2.204054000 | -1.162649000 | -0.879166000 |
| N | 1.283762000 | 1.633070000 | 0.467851000 | N | -1.597114000 | -1.452715000 | 0.294999000 |
| C | 1.646124000 | 0.294846000 | -1.493662000 | C | -1.736576000 | 0.134225000 | -1.514448000 |
| C | 2.354545000 | -0.850048000 | -0.789917000 | C | -2.300266000 | 1.251390000 | -0.653422000 |
| C | 3.484024000 | -1.483934000 | -1.287620000 | C | -3.374350000 | 2.043841000 | -1.032077000 |
| C | 4.064973000 | -2.489846000 | -0.512128000 | C | -3.834390000 | 3.013229000 | -0.139375000 |
| C | 3.495047000 | -2.822511000 | 0.715643000 | C | -3.202708000 | 3.147629000 | 1.098562000 |
| C | 2.354519000 | -2.140658000 | 1.135216000 | C | -2.133554000 | 2.312996000 | 1.405064000 |
| N | 1.803094000 | -1.170113000 | 0.396010000 | N | -1.687549000 | 1.378368000 | 0.546621000 |
| N | 0.162466000 | 0.088961000 | -1.349156000 | N | -0.254898000 | 0.184150000 | -1.406989000 |
| C | -0.332385000 | -1.248816000 | -1.795613000 | C | 0.337017000 | 1.469793000 | -1.832664000 |
| C | -0.613494000 | 1.191972000 | -1.995660000 | C | 0.450751000 | -0.974278000 | -2.001655000 |
| C | -1.958637000 | 1.277954000 | -1.330868000 | C | 1.652360000 | -1.347014000 | -1.163505000 |
| C | -1.405434000 | -1.731551000 | -0.858605000 | C | 1.609730000 | 1.703739000 | -1.059265000 |
| C | -2.446185000 | -2.555454000 | -1.264431000 | C | 2.794754000 | 2.107757000 | -1.661571000 |
| C | -3.367947000 | -3.005002000 | -0.319793000 | C | 3.935167000 | 2.262913000 | -0.872204000 |
| C | -3.214185000 | -2.620235000 | 1.010877000 | C | 3.843744000 | 2.015971000 | 0.496365000 |
| C | -2.164905000 | -1.778607000 | 1.350751000 | C | 2.620133000 | 1.616333000 | 1.024341000 |
| N | -1.280058000 | -1.336039000 | 0.435827000 | N | 1.519906000 | 1.456745000 | 0.267127000 |
| N | -1.893973000 | 1.328553000 | 0.016610000 | N | 1.458399000 | -1.323850000 | 0.176201000 |
| C | -3.037731000 | 1.350932000 | 0.717754000 | C | 2.476840000 | -1.675092000 | 0.984308000 |
| C | -4.287982000 | 1.320066000 | 0.104663000 | C | 3.725551000 | -2.042602000 | 0.496845000 |
| C | -4.351823000 | 1.262666000 | -1.286122000 | C | 3.933790000 | -2.041794000 | -0.881744000 |
| C | -3.164113000 | 1.245607000 | -2.019562000 | C | 2.875505000 | -1.693279000 | -1.722847000 |
| Fe | -0.026228000 | 0.179939000 | 0.756065000 | Fe | -0.079006000 | -0.003014000 | 0.880833000 |
| O | -0.235777000 | 0.299605000 | 2.387045000 | O | 0.360798000 | 0.302202000 | 2.715881000 |
| O | 1.558598000 | 0.375916000 | 3.475513000 | O | -0.223999000 | -1.014378000 | 3.169045000 |
| H | 0.966000000 | 2.636865000 | 2.238805000 | H | -1.423629000 | -2.772010000 | 1.874139000 |
| H | 2.656736000 | 4.436188000 | 1.710503000 | H | -3.191059000 | -4.334809000 | 1.012672000 |
| H | 3.917371000 | 4.303053000 | -0.458228000 | H | -4.324149000 | -3.789262000 | -1.167290000 |
| H | 3.451786000 | 2.382869000 | -2.013319000 | H | -3.641449000 | -1.700070000 | -2.388001000 |
| H | 3.895276000 | -1.204197000 | -2.257144000 | H | -3.834292000 | 1.909820000 | -2.011393000 |
| H | 4.952195000 | -3.013238000 | -0.869194000 | H | -4.670290000 | 3.658992000 | -0.409711000 |
| H | 3.923195000 | -3.604259000 | 1.342591000 | H | -3.530607000 | 3.895752000 | 1.820699000 |
| H | 1.861787000 | -2.358866000 | 2.083528000 | H | -1.599642000 | 2.373427000 | 2.354216000 |
| H | -0.687723000 | -1.209268000 | -2.835214000 | H | 0.514565000 | 1.496615000 | -2.920309000 |
| H | 0.501258000 | -1.964935000 | -1.755838000 | H | -0.369014000 | 2.276621000 | -1.585862000 |
| H | -0.071780000 | 2.133433000 | -1.823340000 | H | -0.232648000 | -1.836456000 | -2.007912000 |
| H | -0.687455000 | 1.026821000 | -3.080208000 | H | 0.742347000 | -0.776499000 | -3.045655000 |
| H | -2.534807000 | -2.832937000 | -2.314471000 | H | 2.827557000 | 2.276554000 | -2.738370000 |
| H | -4.199210000 | -3.642069000 | -0.622418000 | H | 4.883194000 | 2.558232000 | -1.323041000 |
| H | -3.910885000 | -2.948965000 | 1.781599000 | H | 4.711279000 | 2.114448000 | 1.149160000 |
| H | -2.010300000 | -1.407902000 | 2.363145000 | H | 2.501389000 | 1.373152000 | 2.081001000 |
| H | -2.929448000 | 1.363137000 | 1.802936000 | H | 2.269247000 | -1.615162000 | 2.053422000 |
| H | -5.192530000 | 1.325733000 | 0.712453000 | H | 4.524037000 | -2.304546000 | 1.191221000 |
| H | -5.314357000 | 1.217066000 | -1.796684000 | H | 4.908187000 | -2.298734000 | -1.298450000 |
| H | -3.172810000 | 1.185693000 | -3.107947000 | H | 3.002151000 | -1.673019000 | -2.805723000 |
| H | 2.037252000 | 0.180667000 | 2.639799000 | H | -1.116554000 | -0.711400000 | 3.469420000 |
| H | 1.953298000 | 0.378202000 | -2.546651000 | H | -2.099556000 | 0.216421000 | -2.552610000 |

| $^{1}[(N4Py)Fe^{IV}(O)]^{2+}$ | | | | $^{3}[(N4Py)Fe^{IV}(O)]^{2+}$ | | | |
|---|---|---|---|---|---|---|---|
| 51 | | | | 51 | | | |
| symmetry c1 | | | | symmetry c1 | | | |
| C | -2.286259000 | -3.553564000 | 1.014384000 | O | -0.072154000 | -2.317951000 | 0.000000000 |
| C | -3.058196000 | -3.478472000 | -0.142376000 | Fe | 0.015232000 | -0.664732000 | 0.000000000 |
| C | -2.909215000 | -2.387628000 | -1.001701000 | N | 0.164145000 | 1.388474000 | 0.000000000 |
| C | -1.991108000 | -1.411314000 | -0.661664000 | C | 1.673833000 | 1.477180000 | 0.000000000 |
| N | -1.251589000 | -1.489957000 | 0.471507000 | H | 2.041837000 | 2.512172000 | 0.000000000 |
| C | -1.684370000 | -0.158425000 | -1.450623000 | C | 2.119292000 | 0.689648000 | -1.211427000 |
| C | -2.225421000 | 1.003518000 | -0.649299000 | C | 3.126054000 | 1.043971000 | -2.090311000 |
| C | -3.311634000 | 1.790794000 | -0.983492000 | H | 3.664247000 | 1.981053000 | -1.955252000 |
| C | -3.667205000 | 2.824695000 | -0.114669000 | C | 3.411122000 | 0.178135000 | -3.148280000 |
| C | -2.924396000 | 3.035201000 | 1.044623000 | H | 4.195332000 | 0.428909000 | -3.862435000 |
| C | -1.840655000 | 2.208745000 | 1.320442000 | C | 2.680259000 | -0.998874000 | -3.289710000 |
| N | -1.515415000 | 1.210986000 | 0.486693000 | H | 2.876543000 | -1.691254000 | -4.107140000 |
| N | -0.179016000 | -0.014166000 | -1.394250000 | C | 1.677170000 | -1.294101000 | -2.372953000 |
| C | 0.330108000 | 1.286271000 | -1.927846000 | H | 1.064854000 | -2.192065000 | -2.425323000 |
| C | 0.565792000 | -1.190619000 | -1.939210000 | N | 1.418622000 | -0.462226000 | -1.354572000 |
| C | 1.684042000 | -1.556801000 | -1.007606000 | C | -0.476728000 | 1.912591000 | 1.244139000 |
| C | 1.355902000 | 1.852869000 | -0.989279000 | H | -0.875076000 | 2.922235000 | 1.082560000 |
| C | 2.319619000 | 2.771128000 | -1.384236000 | H | 0.287668000 | 1.980948000 | 2.032778000 |
| C | 3.176831000 | 3.308379000 | -0.426931000 | C | -1.539036000 | 0.958721000 | 1.706502000 |
| C | 3.049645000 | 2.910251000 | 0.902240000 | C | -2.598784000 | 1.340606000 | 2.517514000 |
| C | 2.081025000 | 1.975030000 | 1.235779000 | H | -2.715809000 | 2.388831000 | 2.790226000 |
| N | 1.253926000 | 1.464494000 | 0.304691000 | C | -3.492147000 | 0.370913000 | 2.966956000 |
| N | 1.516835000 | -1.199571000 | 0.288733000 | H | -4.332754000 | 0.652291000 | 3.601191000 |
| C | 2.434308000 | -1.543675000 | 1.211887000 | C | -3.302275000 | -0.956810000 | 2.590103000 |
| C | 3.563111000 | -2.272559000 | 0.867484000 | H | -3.979250000 | -1.744218000 | 2.918798000 |
| C | 3.754571000 | -2.636702000 | -0.463691000 | C | -2.235482000 | -1.277664000 | 1.763222000 |
| C | 2.802421000 | -2.272432000 | -1.412687000 | H | -2.040567000 | -2.289153000 | 1.412795000 |
| C | -1.379174000 | -2.538139000 | 1.297013000 | N | -1.374584000 | -0.334903000 | 1.337194000 |
| H | -2.377140000 | -4.394599000 | 1.700398000 | H | -2.040567000 | -2.289153000 | -1.412795000 |
| H | -3.767464000 | -4.270136000 | -0.383330000 | N | -1.374584000 | -0.334903000 | -1.337194000 |
| H | -3.483123000 | -2.301689000 | -1.923016000 | C | -2.235482000 | -1.277664000 | -1.763222000 |
| H | -3.855411000 | 1.606932000 | -1.909052000 | H | -2.715809000 | 2.388831000 | -2.790226000 |
| H | -4.514724000 | 3.468031000 | -0.350584000 | C | -3.492147000 | 0.370913000 | -2.966956000 |
| H | -3.174483000 | 3.836746000 | 1.738326000 | N | 1.418622000 | -0.462226000 | 1.354572000 |
| H | -1.214907000 | 2.318185000 | 2.204166000 | C | -0.476728000 | 1.912591000 | -1.244139000 |
| H | 0.729349000 | 1.161646000 | -2.942354000 | C | 2.680259000 | -0.998874000 | 3.289710000 |
| H | -0.508433000 | 1.995956000 | -1.987395000 | H | -0.875076000 | 2.922235000 | -1.082560000 |
| H | -0.122003000 | -2.047143000 | -2.002143000 | H | 2.876543000 | -1.691254000 | 4.107140000 |
| H | 0.930004000 | -0.985735000 | -2.953905000 | C | 1.677170000 | -1.294101000 | 2.372953000 |
| H | 2.390683000 | 3.057822000 | -2.432741000 | H | 1.064854000 | -2.192065000 | 2.425323000 |
| H | 3.942157000 | 4.028066000 | -0.718265000 | H | 0.287668000 | 1.980948000 | -2.032778000 |
| H | 3.701587000 | 3.305897000 | 1.679880000 | C | -2.598784000 | 1.340606000 | -2.517514000 |
| H | 1.939143000 | 1.602958000 | 2.248331000 | C | -1.539036000 | 0.958721000 | -1.706502000 |
| H | 2.231376000 | -1.209603000 | 2.227428000 | H | -4.332754000 | 0.652291000 | -3.601191000 |
| H | 4.285488000 | -2.535670000 | 1.638978000 | C | -3.302275000 | -0.956810000 | -2.590103000 |
| H | 4.641221000 | -3.194940000 | -0.763691000 | H | -3.979250000 | -1.744218000 | -2.918798000 |
| H | 2.918931000 | -2.537455000 | -2.462989000 | H | 4.195332000 | 0.428909000 | 3.862435000 |
| H | -0.741076000 | -2.534751000 | 2.178385000 | C | 2.119292000 | 0.689648000 | 1.211427000 |
| Fe | 0.012078000 | -0.004083000 | 0.653271000 | H | 3.664247000 | 1.981053000 | 1.955252000 |
| O | 0.129044000 | -0.002329000 | 2.305893000 | C | 3.126054000 | 1.043971000 | 2.090311000 |
| H | -2.072984000 | -0.190873000 | -2.477493000 | C | 3.411122000 | 0.178135000 | 3.148280000 |

$^{5}[(N4Py)Fe^{IV}(O)]^{2+}$

51
symmetry c1

| | | | |
|---|---|---|---|
| C | 2.953119000 | -3.268480000 | -0.891160000 |
| C | 3.615645000 | -3.045628000 | 0.313631000 |
| C | 3.228112000 | -1.979711000 | 1.129091000 |
| C | 2.187584000 | -1.176915000 | 0.697503000 |
| N | 1.553966000 | -1.392103000 | -0.477975000 |
| C | 1.668955000 | 0.039974000 | 1.434870000 |
| C | 2.166182000 | 1.257508000 | 0.684163000 |
| C | 3.190471000 | 2.085064000 | 1.107980000 |
| C | 3.558857000 | 3.148474000 | 0.280359000 |
| C | 2.894134000 | 3.344171000 | -0.927864000 |
| C | 1.874335000 | 2.468620000 | -1.288740000 |
| N | 1.530175000 | 1.446704000 | -0.494168000 |
| N | 0.167146000 | 0.025431000 | 1.279443000 |
| C | -0.487018000 | 1.264800000 | 1.816004000 |
| C | -0.462662000 | -1.218234000 | 1.834686000 |
| C | -1.618566000 | -1.634676000 | 0.973434000 |
| C | -1.645509000 | 1.651223000 | 0.944051000 |
| C | -2.789547000 | 2.273874000 | 1.423108000 |
| C | -3.784325000 | 2.640370000 | 0.516760000 |
| C | -3.608897000 | 2.376288000 | -0.840780000 |
| C | -2.446484000 | 1.739832000 | -1.253140000 |
| N | -1.489749000 | 1.391864000 | -0.375455000 |
| N | -1.469280000 | -1.395627000 | -0.350343000 |
| C | -2.423420000 | -1.771011000 | -1.219283000 |
| C | -3.576188000 | -2.416194000 | -0.793150000 |
| C | -3.744430000 | -2.660294000 | 0.569095000 |
| C | -2.752660000 | -2.264547000 | 1.466504000 |
| C | 1.916556000 | -2.416750000 | -1.261040000 |
| H | 3.230396000 | -4.095627000 | -1.543337000 |
| H | 4.428782000 | -3.701500000 | 0.624793000 |
| H | 3.716614000 | -1.782857000 | 2.082412000 |
| H | 3.680880000 | 1.908977000 | 2.064402000 |
| H | 4.358437000 | 3.823909000 | 0.584999000 |
| H | 3.156495000 | 4.168597000 | -1.589693000 |
| H | 1.313291000 | 2.567246000 | -2.217015000 |
| H | -0.786367000 | 1.118327000 | 2.861776000 |
| H | 0.246819000 | 2.084175000 | 1.789575000 |
| H | 0.285128000 | -2.025139000 | 1.813858000 |
| H | -0.758774000 | -1.064154000 | 2.880315000 |
| H | -2.900418000 | 2.462206000 | 2.490289000 |
| H | -4.696161000 | 3.120898000 | 0.871679000 |
| H | -4.368265000 | 2.643723000 | -1.574374000 |
| H | -2.255185000 | 1.474949000 | -2.292285000 |
| H | -2.238448000 | -1.521133000 | -2.263339000 |
| H | -4.334259000 | -2.705819000 | -1.519796000 |
| H | -4.648279000 | -3.148023000 | 0.934388000 |
| H | -2.857867000 | -2.436648000 | 2.536940000 |
| H | 1.356311000 | -2.536842000 | -2.187324000 |
| Fe | 0.007421000 | 0.009265000 | -0.799233000 |
| O | -0.113626000 | -0.004913000 | -2.441285000 |
| H | 1.971468000 | 0.048300000 | 2.491526000 |

$^{2}[(N4Py)Fe^{III}(OCH_3)]^{2+}$

55
symmetry c1

| | | | |
|---|---|---|---|
| C | 2.266922000 | 1.638755000 | -1.341294000 |
| C | 3.136080000 | 2.709872000 | -1.166256000 |
| C | 2.980576000 | 3.543153000 | -0.059797000 |
| C | 1.956420000 | 3.288240000 | 0.854358000 |
| C | 1.126358000 | 2.207445000 | 0.617226000 |
| N | 1.279509000 | 1.405070000 | -0.464362000 |
| C | -0.043608000 | 1.773012000 | 1.467117000 |
| C | -1.290933000 | 2.089574000 | 0.677546000 |
| C | -2.224430000 | 3.066684000 | 0.973232000 |
| C | -3.317806000 | 3.211080000 | 0.116531000 |
| C | -3.434100000 | 2.376907000 | -0.994088000 |
| C | -2.458345000 | 1.414983000 | -1.230402000 |
| N | -1.406839000 | 1.286695000 | -0.407893000 |
| N | 0.030736000 | 0.256800000 | 1.486416000 |
| C | -1.161406000 | -0.391697000 | 2.113804000 |
| C | 1.311635000 | -0.267914000 | 2.046911000 |
| C | 1.791225000 | -1.413828000 | 1.210813000 |
| C | -1.624057000 | -1.539668000 | 1.269235000 |
| C | -2.420760000 | -2.557798000 | 1.774950000 |
| C | -2.937168000 | -3.513815000 | 0.903966000 |
| C | -2.647367000 | -3.411815000 | -0.454001000 |
| C | -1.824389000 | -2.383823000 | -0.892906000 |
| N | -1.303254000 | -1.473825000 | -0.048797000 |
| N | 1.411961000 | -1.392407000 | -0.095130000 |
| C | 1.926050000 | -2.315401000 | -0.929573000 |
| C | 2.802591000 | -3.302339000 | -0.497762000 |
| C | 3.155949000 | -3.353291000 | 0.847234000 |
| C | 2.642685000 | -2.388825000 | 1.710986000 |
| Fe | -0.000746000 | -0.076439000 | -0.493710000 |
| O | -0.065891000 | -0.035047000 | -2.310016000 |
| H | 2.330891000 | 0.951953000 | -2.182388000 |
| H | 3.927028000 | 2.881567000 | -1.895053000 |
| H | 3.657409000 | 4.382859000 | 0.097531000 |
| H | 1.810423000 | 3.907206000 | 1.738590000 |
| H | -2.105032000 | 3.688153000 | 1.859705000 |
| H | -4.078199000 | 3.964006000 | 0.322961000 |
| H | -4.277427000 | 2.463212000 | -1.678248000 |
| H | -2.485119000 | 0.733044000 | -2.077875000 |
| H | -0.940215000 | -0.698291000 | 3.143986000 |
| H | -1.979258000 | 0.343684000 | 2.155037000 |
| H | 2.069692000 | 0.527843000 | 1.992757000 |
| H | 1.193387000 | -0.542059000 | 3.102765000 |
| H | -2.639157000 | -2.587738000 | 2.842042000 |
| H | -3.561988000 | -4.323368000 | 1.281215000 |
| H | -3.042405000 | -4.124312000 | -1.177229000 |
| H | -1.568212000 | -2.274890000 | -1.939846000 |
| H | 1.619310000 | -2.257437000 | -1.965942000 |
| H | 3.185869000 | -4.025494000 | -1.216938000 |
| H | 3.823210000 | -4.130282000 | 1.220496000 |
| H | 2.902740000 | -2.380543000 | 2.769012000 |
| H | -0.039427000 | 2.213631000 | 2.473388000 |
| C | 0.057815000 | -1.030909000 | -3.302971000 |
| H | 1.018425000 | -0.906452000 | -3.835380000 |
| H | 0.011758000 | -2.064128000 | -2.924063000 |
| H | -0.755716000 | -0.901773000 | -4.037768000 |

| $^{4}[(N4Py)Fe^{III}(OCH_3)]^{2+}$ | | | |
|---|---|---|---|
| 55 | | | |
| symmetry c1 | | | |
| H | 4.640255000 | 2.593400000 | 0.995585000 |
| C | 2.610831000 | 1.884467000 | 0.871492000 |
| H | 2.468533000 | 1.748317000 | 1.943926000 |
| N | 1.566245000 | 1.562723000 | 0.093720000 |
| C | 0.518971000 | -1.165533000 | -1.855630000 |
| H | 0.914983000 | -1.019756000 | -2.869477000 |
| H | -0.212093000 | -1.985986000 | -1.901829000 |
| C | 1.603068000 | -1.550987000 | -0.890925000 |
| C | 2.711691000 | -2.298112000 | -1.264683000 |
| H | 2.839304000 | -2.583613000 | -2.308324000 |
| C | 3.644237000 | -2.665266000 | -0.296731000 |
| H | 4.523669000 | -3.245808000 | -0.575092000 |
| C | 3.443346000 | -2.272033000 | 1.024879000 |
| H | 4.150418000 | -2.534234000 | 1.810836000 |
| C | 2.327556000 | -1.510242000 | 1.338842000 |
| H | 2.129739000 | -1.140564000 | 2.343283000 |
| N | 1.426647000 | -1.161932000 | 0.399432000 |
| O | 0.144129000 | 0.305825000 | 2.460002000 |
| C | -0.793254000 | -0.256683000 | 3.377142000 |
| H | -0.746958000 | 0.316120000 | 4.314985000 |
| H | -0.529816000 | -1.305929000 | 3.584790000 |
| H | -1.816954000 | -0.213518000 | 2.972133000 |
| H | -3.670783000 | -1.760186000 | -2.269770000 |
| C | -3.563131000 | -3.121979000 | -0.577192000 |
| H | -4.359877000 | -3.775879000 | -0.932510000 |
| C | -2.895463000 | -3.403145000 | 0.612895000 |
| C | -3.190293000 | -2.001910000 | -1.322304000 |
| H | -3.151314000 | -4.277176000 | 1.211134000 |
| C | -1.876432000 | -2.550730000 | 1.032922000 |
| H | -1.313520000 | -2.740725000 | 1.945666000 |
| N | -1.535781000 | -1.461787000 | 0.331923000 |
| C | -2.173254000 | 1.224825000 | -0.698727000 |
| C | -3.244091000 | 2.031158000 | -1.039780000 |
| H | -3.766373000 | 1.869732000 | -1.981946000 |
| C | -3.621402000 | 3.046039000 | -0.159056000 |
| H | -4.458851000 | 3.699394000 | -0.403356000 |
| C | -2.911137000 | 3.220320000 | 1.026463000 |
| H | -3.174147000 | 4.005680000 | 1.733951000 |
| C | -1.838452000 | 2.383356000 | 1.308057000 |
| H | -1.235073000 | 2.476695000 | 2.208931000 |
| N | -1.485756000 | 1.400343000 | 0.461779000 |
| C | 0.424250000 | 1.304288000 | -1.991864000 |
| H | -0.299227000 | 2.125966000 | -1.884526000 |
| H | 0.605028000 | 1.148013000 | -3.064044000 |
| C | 1.674335000 | 1.675423000 | -1.244196000 |
| C | 2.841285000 | 2.112614000 | -1.856096000 |
| H | 2.899696000 | 2.178236000 | -2.942374000 |
| C | 3.928387000 | 2.452459000 | -1.049118000 |
| H | 4.862523000 | 2.788072000 | -1.500168000 |
| C | 3.810554000 | 2.342717000 | 0.335161000 |
| Fe | 0.006270000 | 0.152113000 | 0.701928000 |
| N | -0.172098000 | 0.076564000 | -1.380935000 |
| C | -1.673436000 | 0.053171000 | -1.511474000 |
| H | -1.995283000 | 0.103688000 | -2.561675000 |
| C | -2.171975000 | -1.203388000 | -0.826167000 |

| $^{6}[(N4Py)Fe^{III}(OCH_3)]^{2+}$ | | | |
|---|---|---|---|
| 55 | | | |
| symmetry c1 | | | |
| Fe | 0.025977000 | -0.081680000 | -0.842043000 |
| N | 0.274277000 | -0.021849000 | 1.414924000 |
| C | 1.762728000 | 0.012199000 | 1.481373000 |
| H | 2.144367000 | 0.035513000 | 2.514197000 |
| C | 2.276103000 | -1.206820000 | 0.735933000 |
| C | 3.331827000 | -1.990489000 | 1.171402000 |
| H | 3.828329000 | -1.766543000 | 2.114840000 |
| C | 3.723471000 | -3.071682000 | 0.379819000 |
| H | 4.546627000 | -3.711680000 | 0.697935000 |
| C | 3.049309000 | -3.329296000 | -0.811687000 |
| H | 3.326614000 | -4.168297000 | -1.448660000 |
| C | 2.000535000 | -2.494835000 | -1.185105000 |
| H | 1.433945000 | -2.646409000 | -2.103527000 |
| N | 1.629829000 | -1.454060000 | -0.424353000 |
| C | 2.218921000 | 1.231031000 | 0.700274000 |
| C | 3.242110000 | 2.071798000 | 1.106113000 |
| H | 3.752336000 | 1.898130000 | 2.053002000 |
| C | 3.583536000 | 3.143662000 | 0.279234000 |
| H | 4.379442000 | 3.828583000 | 0.572765000 |
| C | 2.893626000 | 3.334326000 | -0.915706000 |
| H | 3.130342000 | 4.164031000 | -1.580848000 |
| C | 1.879784000 | 2.443978000 | -1.255777000 |
| H | 1.306872000 | 2.546915000 | -2.175239000 |
| N | 1.556556000 | 1.413186000 | -0.462345000 |
| C | -0.383606000 | 1.200036000 | 1.938281000 |
| H | 0.305221000 | 2.050236000 | 1.822721000 |
| H | -0.613186000 | 1.103505000 | 3.009762000 |
| C | -1.623179000 | 1.498077000 | 1.136954000 |
| C | -2.789619000 | 1.987379000 | 1.711399000 |
| H | -2.839996000 | 2.144889000 | 2.788555000 |
| C | -3.885897000 | 2.252745000 | 0.891787000 |
| H | -4.816263000 | 2.622654000 | 1.323667000 |
| C | -3.785565000 | 2.021515000 | -0.478934000 |
| H | -4.623196000 | 2.206512000 | -1.150406000 |
| C | -2.591586000 | 1.522144000 | -0.982951000 |
| H | -2.468012000 | 1.283319000 | -2.037659000 |
| N | -1.534060000 | 1.270602000 | -0.192069000 |
| C | -0.327465000 | -1.261309000 | 1.969048000 |
| H | -0.599602000 | -1.132488000 | 3.026850000 |
| H | 0.416778000 | -2.070360000 | 1.916750000 |
| C | -1.517372000 | -1.684874000 | 1.146996000 |
| C | -2.658111000 | -2.243192000 | 1.709264000 |
| H | -2.732490000 | -2.353585000 | 2.790780000 |
| C | -3.699187000 | -2.641325000 | 0.871192000 |
| H | -4.609489000 | -3.067941000 | 1.293841000 |
| C | -3.570703000 | -2.475866000 | -0.506717000 |
| H | -4.365265000 | -2.769924000 | -1.191503000 |
| C | -2.407981000 | -1.900140000 | -1.001077000 |
| H | -2.256630000 | -1.708871000 | -2.063288000 |
| N | -1.406415000 | -1.515709000 | -0.189474000 |
| O | -0.199257000 | 0.003663000 | -2.624536000 |
| C | -0.280939000 | 0.843188000 | -3.755036000 |
| H | -0.982494000 | 0.416566000 | -4.489976000 |
| H | 0.712585000 | 0.936219000 | -4.224259000 |
| H | -0.632878000 | 1.848248000 | -3.467213000 |

| $^{2}[(N4Py)Fe^{III}(OH_2)]^{3+}$ | | | | $^{4}[(N4Py)Fe^{III}(OH_2)]^{3+}$ | | | |
|---|---|---|---|---|---|---|---|
| 53 | | | | 53 | | | |
| symmetry c1 | | | | symmetry c1 | | | |
| C | 1.720626000 | -2.344969000 | -1.309359000 | C | 1.675336000 | 2.392930000 | 1.281790000 |
| C | 2.770769000 | -3.220742000 | -1.056083000 | C | 2.708296000 | 3.281232000 | 1.007939000 |
| C | 3.536682000 | -3.056242000 | 0.094135000 | C | 3.470982000 | 3.102315000 | -0.142508000 |
| C | 3.234821000 | -2.022176000 | 0.984268000 | C | 3.186067000 | 2.040504000 | -1.004485000 |
| C | 2.178109000 | -1.190239000 | 0.675706000 | C | 2.151843000 | 1.186984000 | -0.672572000 |
| N | 1.448258000 | -1.343724000 | -0.459011000 | N | 1.426064000 | 1.363285000 | 0.458326000 |
| C | 1.679449000 | -0.021039000 | 1.484489000 | C | 1.698085000 | -0.006534000 | -1.485659000 |
| C | 2.054719000 | 1.228516000 | 0.725722000 | C | 2.076842000 | -1.245008000 | -0.701356000 |
| C | 3.009214000 | 2.156214000 | 1.093331000 | C | 3.053579000 | -2.155166000 | -1.056514000 |
| C | 3.219214000 | 3.249128000 | 0.249109000 | C | 3.267211000 | -3.256705000 | -0.224177000 |
| C | 2.462657000 | 3.382735000 | -0.913434000 | C | 2.487524000 | -3.422412000 | 0.917156000 |
| C | 1.511012000 | 2.419195000 | -1.220177000 | C | 1.511474000 | -2.479537000 | 1.213392000 |
| N | 1.336961000 | 1.355579000 | -0.420540000 | N | 1.340071000 | -1.406399000 | 0.425343000 |
| N | 0.158031000 | -0.103006000 | 1.377009000 | N | 0.199239000 | 0.039748000 | -1.498613000 |
| C | -0.542606000 | 1.097254000 | 1.966672000 | C | -0.478981000 | -1.174608000 | -2.009960000 |
| C | -0.407290000 | -1.400136000 | 1.884400000 | C | -0.400989000 | 1.310603000 | -1.966412000 |
| C | -1.527609000 | -1.808488000 | 0.982770000 | C | -1.477774000 | 1.767344000 | -1.017787000 |
| C | -1.572981000 | 1.615675000 | 1.010863000 | C | -1.546709000 | -1.640043000 | -1.053472000 |
| C | -2.621643000 | 2.436312000 | 1.397865000 | C | -2.605882000 | -2.442797000 | -1.456929000 |
| C | -3.467632000 | 2.953868000 | 0.419779000 | C | -3.515708000 | -2.900094000 | -0.508163000 |
| C | -3.240969000 | 2.642301000 | -0.919846000 | C | -3.349262000 | -2.544553000 | 0.829056000 |
| C | -2.184018000 | 1.807304000 | -1.247275000 | C | -2.286136000 | -1.726573000 | 1.176047000 |
| N | -1.377826000 | 1.304016000 | -0.294597000 | N | -1.410905000 | -1.295047000 | 0.247845000 |
| N | -1.429749000 | -1.349880000 | -0.292959000 | N | -1.383490000 | 1.359690000 | 0.270696000 |
| C | -2.376799000 | -1.667111000 | -1.195252000 | C | -2.290902000 | 1.753556000 | 1.184466000 |
| C | -3.442317000 | -2.491324000 | -0.869148000 | C | -3.326698000 | 2.609984000 | 0.848901000 |
| C | -3.547059000 | -2.970516000 | 0.434475000 | C | -3.440380000 | 3.038358000 | -0.472002000 |
| C | -2.581168000 | -2.616406000 | 1.375445000 | C | -2.510761000 | 2.606384000 | -1.413729000 |
| Fe | -0.010264000 | -0.051617000 | -0.558718000 | Fe | -0.001861000 | 0.015251000 | 0.694686000 |
| O | -0.019796000 | 0.062964000 | -2.545742000 | O | 0.074688000 | -0.070295000 | 2.794798000 |
| H | 1.082079000 | -2.436391000 | -2.185132000 | H | 1.038066000 | 2.485354000 | 2.159035000 |
| H | 2.974455000 | -4.023930000 | -1.762392000 | H | 2.899126000 | 4.104498000 | 1.694428000 |
| H | 4.360616000 | -3.736348000 | 0.308499000 | H | 4.278719000 | 3.794249000 | -0.379898000 |
| H | 3.798215000 | -1.873432000 | 1.904147000 | H | 3.745379000 | 1.885758000 | -1.925994000 |
| H | 3.563260000 | 2.034654000 | 2.022671000 | H | 3.624251000 | -2.011998000 | -1.972853000 |
| H | 3.962082000 | 4.002647000 | 0.509808000 | H | 4.030400000 | -3.991466000 | -0.479488000 |
| H | 2.596536000 | 4.232443000 | -1.580895000 | H | 2.618928000 | -4.278963000 | 1.576356000 |
| H | 0.871280000 | 2.481287000 | -2.098166000 | H | 0.855007000 | -2.564453000 | 2.077566000 |
| H | -0.975338000 | 0.837175000 | 2.939249000 | H | -0.901426000 | -0.993925000 | -3.007276000 |
| H | 0.206114000 | 1.886269000 | 2.130480000 | H | 0.259348000 | -1.984419000 | -2.111609000 |
| H | 0.379985000 | -2.166574000 | 1.840857000 | H | 0.377415000 | 2.088077000 | -1.997831000 |
| H | -0.720939000 | -1.296393000 | 2.929745000 | H | -0.796278000 | 1.205091000 | -2.985678000 |
| H | -2.765570000 | 2.667066000 | 2.452445000 | H | -2.708771000 | -2.701689000 | -2.510067000 |
| H | -4.300942000 | 3.596399000 | 0.703232000 | H | -4.355342000 | -3.524748000 | -0.812193000 |
| H | -3.880649000 | 3.029616000 | -1.711106000 | H | -4.038495000 | -2.881069000 | 1.601946000 |
| H | -1.964651000 | 1.520795000 | -2.272946000 | H | -2.113435000 | -1.405399000 | 2.201245000 |
| H | -2.284806000 | -1.235264000 | -2.187917000 | H | -2.181753000 | 1.363050000 | 2.192921000 |
| H | -4.183821000 | -2.733118000 | -1.628599000 | H | -4.038988000 | 2.913194000 | 1.614599000 |
| H | -4.384791000 | -3.604747000 | 0.723441000 | H | -4.258330000 | 3.693717000 | -0.770754000 |
| H | -2.643392000 | -2.957652000 | 2.407615000 | H | -2.579752000 | 2.911676000 | -2.457015000 |
| H | 0.880692000 | 0.049516000 | -2.952978000 | H | 0.957030000 | -0.158347000 | 3.229986000 |
| H | 2.020206000 | -0.022923000 | 2.527394000 | H | 2.134507000 | -0.008271000 | -2.494427000 |
| H | -0.560378000 | -0.546845000 | -3.101691000 | H | -0.493593000 | 0.397295000 | 3.451288000 |

| $^{6}[(N4Py)Fe^{III}(OH_2)]^{3+}$ | | | | $^{2}[(N4Py)Fe^{III}(OH)]^{2+}$ | | | |
|---|---|---|---|---|---|---|---|
| 53 | | | | 52 | | | |
| symmetry c1 | | | | symmetry c1 | | | |
| C | 1.855808000 | 2.476174000 | 1.184583000 | C | 1.592613000 | -1.274910000 | 2.398290000 |
| C | 2.873098000 | 3.345121000 | 0.806734000 | C | 2.598615000 | -1.003834000 | 3.320222000 |
| C | 3.541564000 | 3.120591000 | -0.394025000 | C | 3.375490000 | 0.142211000 | 3.167662000 |
| C | 3.178924000 | 2.036675000 | -1.197508000 | C | 3.128600000 | 1.006607000 | 2.097765000 |
| C | 2.160013000 | 1.212137000 | -0.758919000 | C | 2.116491000 | 0.676831000 | 1.215961000 |
| N | 1.524802000 | 1.427722000 | 0.415073000 | N | 1.375115000 | -0.450094000 | 1.364652000 |
| C | 1.663538000 | -0.015226000 | -1.494179000 | C | 1.690691000 | 1.469330000 | 0.000000000 |
| C | 2.165322000 | -1.223762000 | -0.731676000 | C | 2.116491000 | 0.676831000 | -1.215961000 |
| C | 3.191501000 | -2.050077000 | -1.149358000 | C | 3.128600000 | 1.006607000 | -2.097765000 |
| C | 3.558718000 | -3.114400000 | -0.322135000 | C | 3.375490000 | 0.142211000 | -3.167662000 |
| C | 2.887757000 | -3.318129000 | 0.880888000 | C | 2.598615000 | -1.003834000 | -3.320222000 |
| C | 1.864011000 | -2.447867000 | 1.237695000 | C | 1.592613000 | -1.274910000 | -2.398290000 |
| N | 1.527890000 | -1.418590000 | 0.444876000 | N | 1.375115000 | -0.450094000 | -1.364652000 |
| N | 0.169210000 | -0.017901000 | -1.374864000 | N | 0.168813000 | 1.410427000 | 0.000000000 |
| C | -0.478411000 | -1.263886000 | -1.869106000 | C | -0.458615000 | 1.954711000 | -1.249478000 |
| C | -0.487492000 | 1.212351000 | -1.896101000 | C | -0.458615000 | 1.954711000 | 1.249478000 |
| C | -1.635164000 | 1.607042000 | -1.009971000 | C | -1.516098000 | 1.006452000 | 1.737408000 |
| C | -1.629658000 | -1.640671000 | -0.980170000 | C | -1.516098000 | 1.006452000 | -1.737408000 |
| C | -2.783559000 | -2.247892000 | -1.453053000 | C | -2.555824000 | 1.383855000 | -2.575317000 |
| C | -3.781539000 | -2.604101000 | -0.546496000 | C | -3.436253000 | 0.408269000 | -3.040351000 |
| C | -3.598476000 | -2.353804000 | 0.812224000 | C | -3.253464000 | -0.918049000 | -2.653649000 |
| C | -2.425368000 | -1.738102000 | 1.220994000 | C | -2.205927000 | -1.233611000 | -1.799184000 |
| N | -1.467129000 | -1.391176000 | 0.340752000 | N | -1.360045000 | -0.285344000 | -1.358464000 |
| N | -1.458678000 | 1.399948000 | 0.316206000 | N | -1.360045000 | -0.285344000 | 1.358464000 |
| C | -2.406564000 | 1.774264000 | 1.196413000 | C | -2.205927000 | -1.233611000 | 1.799184000 |
| C | -3.586707000 | 2.371475000 | 0.780307000 | C | -3.253464000 | -0.918049000 | 2.653649000 |
| C | -3.787199000 | 2.573029000 | -0.584137000 | C | -3.436253000 | 0.408269000 | 3.040351000 |
| C | -2.797439000 | 2.192050000 | -1.490031000 | C | -2.555824000 | 1.383855000 | 2.575317000 |
| O | 0.121564000 | 0.073921000 | 2.887751000 | Fe | -0.010345000 | -0.579068000 | 0.000000000 |
| H | 1.287382000 | 2.607273000 | 2.104490000 | O | -0.086289000 | -2.430117000 | 0.000000000 |
| H | 3.126652000 | 4.187386000 | 1.448772000 | H | 0.940402000 | -2.144651000 | 2.458206000 |
| H | 4.338430000 | 3.792001000 | -0.713715000 | H | 2.759858000 | -1.688077000 | 4.152152000 |
| H | 3.669578000 | 1.842972000 | -2.150186000 | H | 4.162454000 | 0.373879000 | 3.885175000 |
| H | 3.684113000 | -1.872890000 | -2.104254000 | H | 3.699996000 | 1.923313000 | 1.958553000 |
| H | 4.360868000 | -3.787168000 | -0.625274000 | H | 3.699996000 | 1.923313000 | -1.958553000 |
| H | 3.144940000 | -4.144672000 | 1.541649000 | H | 4.162454000 | 0.373879000 | -3.885175000 |
| H | 1.295716000 | -2.561333000 | 2.159970000 | H | 2.759858000 | -1.688077000 | -4.152152000 |
| H | -0.795971000 | -1.145691000 | -2.913676000 | H | 0.940402000 | -2.144651000 | -2.458206000 |
| H | 0.256011000 | -2.082357000 | -1.835354000 | H | -0.856277000 | 2.962135000 | -1.075612000 |
| H | 0.243095000 | 2.035081000 | -1.886029000 | H | 0.317849000 | 2.033111000 | -2.024924000 |
| H | -0.809929000 | 1.067192000 | -2.935598000 | H | 0.317849000 | 2.033111000 | 2.024924000 |
| H | -2.901050000 | -2.429696000 | -2.520528000 | H | -0.856277000 | 2.962135000 | 1.075612000 |
| H | -4.702083000 | -3.067227000 | -0.901493000 | H | -2.668677000 | 2.429883000 | -2.857987000 |
| H | -4.354863000 | -2.616453000 | 1.550047000 | H | -4.261441000 | 0.684524000 | -3.696842000 |
| H | -2.237898000 | -1.505577000 | 2.268210000 | H | -3.919907000 | -1.707323000 | -2.999067000 |
| H | -2.202527000 | 1.578795000 | 2.248299000 | H | -2.004968000 | -2.242456000 | -1.442142000 |
| H | -4.335966000 | 2.657170000 | 1.516989000 | H | -2.004968000 | -2.242456000 | 1.442142000 |
| H | -4.714304000 | 3.018815000 | -0.944317000 | H | -3.919907000 | -1.707323000 | 2.999067000 |
| H | -2.927712000 | 2.337948000 | -2.561524000 | H | -4.261441000 | 0.684524000 | 3.696842000 |
| H | 0.980341000 | 0.080606000 | 3.377493000 | H | -2.668677000 | 2.429883000 | 2.857987000 |
| H | 1.994778000 | -0.026048000 | -2.542379000 | H | 0.799819000 | -2.852384000 | 0.000000000 |
| H | -0.587493000 | -0.045499000 | 3.564489000 | H | 2.068309000 | 2.500598000 | 0.000000000 |
| Fe | 0.011499000 | 0.006855000 | 0.855385000 | | | | |

| $^{4}$[(N4Py)Fe$^{III}$(OH)]$^{2+}$ | | | | $^{6}$[(N4Py)Fe$^{III}$(OH)]$^{2+}$ | | | |
|---|---|---|---|---|---|---|---|
| 52 | | | | 52 | | | |
| symmetry c1 | | | | symmetry c1 | | | |
| C | -1.654808000 | 1.270732000 | 2.410818000 | C | 1.925150000 | -1.263864000 | 2.466771000 |
| C | -2.661802000 | 0.975896000 | 3.322719000 | C | 2.949249000 | -0.889793000 | 3.331244000 |
| C | -3.408114000 | -0.188522000 | 3.157651000 | C | 3.611876000 | 0.315107000 | 3.109202000 |
| C | -3.128103000 | -1.038866000 | 2.086693000 | C | 3.233410000 | 1.121056000 | 2.033685000 |
| C | -2.113972000 | -0.685795000 | 1.212980000 | C | 2.202957000 | 0.683838000 | 1.218564000 |
| N | -1.404273000 | 0.455232000 | 1.375560000 | N | 1.570377000 | -0.489965000 | 1.430699000 |
| C | -1.687765000 | -1.489894000 | 0.000000000 | C | 1.702220000 | 1.436915000 | 0.000000000 |
| C | -2.113972000 | -0.685795000 | -1.212980000 | C | 2.202957000 | 0.683838000 | -1.218564000 |
| C | -3.128103000 | -1.038866000 | -2.086693000 | C | 3.233410000 | 1.121056000 | -2.033685000 |
| C | -3.408114000 | -0.188522000 | -3.157651000 | C | 3.611876000 | 0.315107000 | -3.109202000 |
| C | -2.661802000 | 0.975896000 | -3.322719000 | C | 2.949249000 | -0.889793000 | -3.331244000 |
| C | -1.654808000 | 1.270732000 | -2.410818000 | C | 1.925150000 | -1.263864000 | -2.466771000 |
| N | -1.404273000 | 0.455232000 | -1.375560000 | N | 1.570377000 | -0.489965000 | -1.430699000 |
| N | -0.199306000 | -1.506204000 | 0.000000000 | N | 0.215702000 | 1.332475000 | 0.000000000 |
| C | 0.446569000 | -1.980501000 | -1.236356000 | C | -0.428137000 | 1.852561000 | -1.231275000 |
| C | 0.446569000 | -1.980501000 | 1.236356000 | C | -0.428137000 | 1.852561000 | 1.231275000 |
| C | 1.515026000 | -1.018940000 | 1.699290000 | C | -1.605468000 | 0.993982000 | 1.612412000 |
| C | 1.515026000 | -1.018940000 | -1.699290000 | C | -1.605468000 | 0.993982000 | -1.612412000 |
| C | 2.567131000 | -1.423616000 | -2.513173000 | C | -2.758108000 | 1.511709000 | -2.189203000 |
| C | 3.479774000 | -0.478440000 | -2.973203000 | C | -3.783859000 | 0.639295000 | -2.551087000 |
| C | 3.320553000 | 0.856481000 | -2.605659000 | C | -3.629482000 | -0.728550000 | -2.331994000 |
| C | 2.262918000 | 1.202125000 | -1.778057000 | C | -2.456067000 | -1.178745000 | -1.742815000 |
| N | 1.383777000 | 0.278834000 | -1.341565000 | N | -1.469681000 | -0.332723000 | -1.393921000 |
| N | 1.383777000 | 0.278834000 | 1.341565000 | N | -1.469681000 | -0.332723000 | 1.393921000 |
| C | 2.262918000 | 1.202125000 | 1.778057000 | C | -2.456067000 | -1.178745000 | 1.742815000 |
| C | 3.320553000 | 0.856481000 | 2.605659000 | C | -3.629482000 | -0.728550000 | 2.331994000 |
| C | 3.479774000 | -0.478440000 | 2.973203000 | C | -3.783859000 | 0.639295000 | 2.551087000 |
| C | 2.567131000 | -1.423616000 | 2.513173000 | C | -2.758108000 | 1.511709000 | 2.189203000 |
| Fe | 0.008272000 | 0.768496000 | 0.000000000 | Fe | 0.015985000 | -0.946604000 | 0.000000000 |
| O | 0.097562000 | 2.624883000 | 0.000000000 | O | -0.087148000 | -2.769829000 | 0.000000000 |
| H | -1.029321000 | 2.158724000 | 2.484554000 | H | 1.366595000 | -2.191367000 | 2.589209000 |
| H | -2.847727000 | 1.655741000 | 4.153097000 | H | 3.216113000 | -1.537250000 | 4.165630000 |
| H | -4.197665000 | -0.442355000 | 3.864981000 | H | 4.415982000 | 0.633136000 | 3.773141000 |
| H | -3.676412000 | -1.968210000 | 1.936904000 | H | 3.720808000 | 2.075440000 | 1.837825000 |
| H | -3.676412000 | -1.968210000 | -1.936904000 | H | 3.720808000 | 2.075440000 | -1.837825000 |
| H | -4.197665000 | -0.442355000 | -3.864981000 | H | 4.415982000 | 0.633136000 | -3.773141000 |
| H | -2.847727000 | 1.655741000 | -4.153097000 | H | 3.216113000 | -1.537250000 | -4.165630000 |
| H | -1.029321000 | 2.158724000 | -2.484554000 | H | 1.366595000 | -2.191367000 | -2.589209000 |
| H | 0.866629000 | -2.988758000 | -1.106530000 | H | -0.724312000 | 2.904887000 | -1.112013000 |
| H | -0.306755000 | -2.048891000 | -2.036541000 | H | 0.297124000 | 1.807313000 | -2.057881000 |
| H | -0.306755000 | -2.048891000 | 2.036541000 | H | 0.297124000 | 1.807313000 | 2.057881000 |
| H | 0.866629000 | -2.988758000 | 1.106530000 | H | -0.724312000 | 2.904887000 | 1.112013000 |
| H | 2.662187000 | -2.475954000 | -2.779944000 | H | -2.852408000 | 2.586301000 | -2.343173000 |
| H | 4.312112000 | -0.782688000 | -3.607927000 | H | -4.702114000 | 1.027495000 | -2.992690000 |
| H | 4.012169000 | 1.627919000 | -2.941974000 | H | -4.410584000 | -1.439774000 | -2.597550000 |
| H | 2.089374000 | 2.221870000 | -1.438547000 | H | -2.282081000 | -2.231184000 | -1.519020000 |
| H | 2.089374000 | 2.221870000 | 1.438547000 | H | -2.282081000 | -2.231184000 | 1.519020000 |
| H | 4.012169000 | 1.627919000 | 2.941974000 | H | -4.410584000 | -1.439774000 | 2.597550000 |
| H | 4.312112000 | -0.782688000 | 3.607927000 | H | -4.702114000 | 1.027495000 | 2.992690000 |
| H | 2.662187000 | -2.475954000 | 2.779944000 | H | -2.852408000 | 2.586301000 | 2.343173000 |
| H | -0.714203000 | 3.178772000 | 0.000000000 | H | 0.687728000 | -3.375340000 | 0.000000000 |
| H | -2.141700000 | -2.492755000 | 0.000000000 | H | 2.059402000 | 2.478465000 | 0.000000000 |

$\{[(N4Py)Fe^{II}(OOH)(\mathbf{MeOH})]^{+}\}$-**a** (S=0)

| | | | |
|---|---|---|---|
| 59 | | | |
| symmetry c1 | | | |
| C | 2.214546000 | 1.499865000 | -1.641025000 |
| C | 3.260716000 | 2.414985000 | -1.654587000 |
| C | 3.538649000 | 3.159781000 | -0.506996000 |
| C | 2.766928000 | 2.956627000 | 0.640815000 |
| C | 1.742833000 | 2.026973000 | 0.586516000 |
| N | 1.459065000 | 1.320375000 | -0.541475000 |
| C | 0.833393000 | 1.629512000 | 1.726947000 |
| C | -0.553779000 | 2.116540000 | 1.375492000 |
| C | -1.245063000 | 3.132291000 | 2.012427000 |
| C | -2.535738000 | 3.435199000 | 1.569399000 |
| C | -3.078091000 | 2.705162000 | 0.511022000 |
| C | -2.327559000 | 1.698372000 | -0.085847000 |
| N | -1.081194000 | 1.412758000 | 0.335197000 |
| N | 0.747670000 | 0.112463000 | 1.651707000 |
| C | -0.287801000 | -0.480542000 | 2.551066000 |
| C | 2.067137000 | -0.581370000 | 1.750746000 |
| C | 2.185002000 | -1.619983000 | 0.666073000 |
| C | -1.054164000 | -1.536098000 | 1.800991000 |
| C | -1.713492000 | -2.587609000 | 2.424761000 |
| C | -2.431592000 | -3.495941000 | 1.646367000 |
| C | -2.455087000 | -3.330109000 | 0.261835000 |
| C | -1.765315000 | -2.263567000 | -0.299601000 |
| N | -1.092026000 | -1.374220000 | 0.454251000 |
| N | 1.422297000 | -1.404952000 | -0.435280000 |
| C | 1.522563000 | -2.239893000 | -1.486277000 |
| C | 2.384713000 | -3.328910000 | -1.477344000 |
| C | 3.157500000 | -3.569866000 | -0.341604000 |
| C | 3.055462000 | -2.700089000 | 0.744313000 |
| Fe | 0.097313000 | 0.009180000 | -0.241323000 |
| O | -0.518352000 | -0.218362000 | -1.928601000 |
| O | -0.739172000 | 1.212026000 | -2.761654000 |
| H | 1.954442000 | 0.878723000 | -2.495063000 |
| H | 3.855098000 | 2.535615000 | -2.560306000 |
| H | 4.360017000 | 3.876704000 | -0.497495000 |
| H | 2.969373000 | 3.492957000 | 1.567714000 |
| H | -0.788973000 | 3.662231000 | 2.848412000 |
| H | -3.113193000 | 4.222430000 | 2.054410000 |
| H | -4.083157000 | 2.911039000 | 0.142837000 |
| H | -2.692586000 | 1.110935000 | -0.923156000 |
| H | 0.160169000 | -0.877450000 | 3.472703000 |
| H | -0.991608000 | 0.313531000 | 2.842163000 |
| H | 2.864839000 | 0.160989000 | 1.596760000 |
| H | 2.214497000 | -1.017609000 | 2.748146000 |
| H | -1.657758000 | -2.693658000 | 3.508147000 |
| H | -2.954410000 | -4.329863000 | 2.116143000 |
| H | -2.992107000 | -4.025377000 | -0.383195000 |
| H | -1.722029000 | -2.074855000 | -1.370888000 |
| H | 0.876947000 | -1.988722000 | -2.328470000 |
| H | 2.437875000 | -3.982061000 | -2.348133000 |
| H | 3.829836000 | -4.427263000 | -0.298116000 |
| H | 3.645681000 | -2.854232000 | 1.647904000 |
| H | -0.180597000 | 0.964822000 | -3.534344000 |
| H | 1.183072000 | 1.998229000 | 2.702404000 |
| C | -3.696397000 | -0.561460000 | -2.788054000 |
| H | -4.746847000 | -0.730438000 | -3.067276000 |
| H | -3.072612000 | -1.322856000 | -3.284806000 |
| O | -3.341222000 | 0.769292000 | -3.185276000 |
| H | -3.606841000 | -0.707575000 | -1.696952000 |
| H | -2.344081000 | 0.875843000 | -3.057980000 |

$\{[(N4Py)Fe^{II}(OOH)(\mathbf{MeOH})]^{+}\}$-**a** (S=1)

| | | | |
|---|---|---|---|
| 59 | | | |
| symmetry c1 | | | |
| C | 2.185400000 | 1.455700000 | -1.729200000 |
| C | 3.177600000 | 2.429100000 | -1.772900000 |
| C | 3.455400000 | 3.173600000 | -0.628500000 |
| C | 2.734200000 | 2.932600000 | 0.543200000 |
| C | 1.757300000 | 1.953700000 | 0.517700000 |
| N | 1.493200000 | 1.237600000 | -0.602100000 |
| C | 0.852400000 | 1.563300000 | 1.664600000 |
| C | -0.533700000 | 2.042600000 | 1.302300000 |
| C | -1.209600000 | 3.079500000 | 1.917400000 |
| C | -2.490500000 | 3.395800000 | 1.457100000 |
| C | -3.032200000 | 2.665300000 | 0.404800000 |
| C | -2.301200000 | 1.629400000 | -0.174400000 |
| N | -1.072100000 | 1.334800000 | 0.274800000 |
| N | 0.772400000 | 0.053900000 | 1.624200000 |
| C | -0.271100000 | -0.524000000 | 2.526900000 |
| C | 2.097600000 | -0.623400000 | 1.754200000 |
| C | 2.220700000 | -1.705100000 | 0.720800000 |
| C | -1.056600000 | -1.573400000 | 1.792900000 |
| C | -1.771300000 | -2.571600000 | 2.441800000 |
| C | -2.513800000 | -3.475700000 | 1.685500000 |
| C | -2.508300000 | -3.366600000 | 0.296100000 |
| C | -1.767700000 | -2.354700000 | -0.296900000 |
| N | -1.073800000 | -1.469500000 | 0.442100000 |
| N | 1.466000000 | -1.546500000 | -0.392500000 |
| C | 1.563400000 | -2.424400000 | -1.408200000 |
| C | 2.433700000 | -3.503200000 | -1.347700000 |
| C | 3.209100000 | -3.684500000 | -0.203900000 |
| C | 3.098900000 | -2.773800000 | 0.844100000 |
| Fe | 0.121000000 | -0.123900000 | -0.317400000 |
| O | -0.352800000 | -0.241000000 | -1.912500000 |
| O | -1.483200000 | 1.870800000 | -3.299600000 |
| H | 1.916700000 | 0.833600000 | -2.581000000 |
| H | 3.725500000 | 2.592800000 | -2.700200000 |
| H | 4.236200000 | 3.934400000 | -0.642200000 |
| H | 2.931400000 | 3.484500000 | 1.461400000 |
| H | -0.748200000 | 3.615800000 | 2.745400000 |
| H | -3.056800000 | 4.201200000 | 1.925200000 |
| H | -4.025800000 | 2.884100000 | 0.013600000 |
| H | -2.711400000 | 1.078000000 | -1.031000000 |
| H | 0.183700000 | -0.925000000 | 3.442000000 |
| H | -0.961300000 | 0.278500000 | 2.826400000 |
| H | 2.889700000 | 0.116900000 | 1.566800000 |
| H | 2.242000000 | -1.010100000 | 2.771100000 |
| H | -1.738400000 | -2.635700000 | 3.529000000 |
| H | -3.081000000 | -4.265900000 | 2.177600000 |
| H | -3.061200000 | -4.064400000 | -0.331600000 |
| H | -1.702300000 | -2.219400000 | -1.372900000 |
| H | 0.916000000 | -2.230200000 | -2.261100000 |
| H | 2.489200000 | -4.193800000 | -2.188100000 |
| H | 3.890000000 | -4.532100000 | -0.124500000 |
| H | 3.688400000 | -2.882400000 | 1.754000000 |
| H | -0.923000000 | 1.223100000 | -2.804100000 |
| H | 1.199600000 | 1.946500000 | 2.633900000 |
| C | -3.532400000 | -0.512600000 | -2.701200000 |
| H | -3.992100000 | -1.048000000 | -3.566800000 |
| H | -2.443100000 | -0.775100000 | -2.727900000 |
| O | -3.738500000 | 0.863100000 | -2.707500000 |
| H | -3.919500000 | -1.012100000 | -1.779800000 |
| H | -2.451000000 | 1.483500000 | -3.091400000 |

## {[(N4Py)$Fe^{II}$(OOH)(**MeOH**)]$^{+}$}-**a** (S=2)

59
symmetry c1

| | | | |
|---|---|---|---|
| C | 2.331900000 | 1.953900000 | -1.669800000 |
| C | 3.303700000 | 2.935800000 | -1.510600000 |
| C | 3.484500000 | 3.509900000 | -0.251700000 |
| C | 2.680900000 | 3.086200000 | 0.808000000 |
| C | 1.728200000 | 2.106700000 | 0.566900000 |
| N | 1.554300000 | 1.545800000 | -0.652100000 |
| C | 0.758300000 | 1.602700000 | 1.623000000 |
| C | -0.591500000 | 2.200200000 | 1.268700000 |
| C | -1.163300000 | 3.240200000 | 1.987800000 |
| C | -2.377900000 | 3.767800000 | 1.548600000 |
| C | -2.963000000 | 3.238000000 | 0.399100000 |
| C | -2.330800000 | 2.190300000 | -0.264200000 |
| N | -1.166200000 | 1.666600000 | 0.163200000 |
| N | 0.657900000 | 0.129100000 | 1.485200000 |
| C | -0.409500000 | -0.489800000 | 2.298600000 |
| C | 1.945300000 | -0.584900000 | 1.620200000 |
| C | 1.998500000 | -1.757700000 | 0.670300000 |
| C | -0.942900000 | -1.704500000 | 1.581000000 |
| C | -1.203500000 | -2.906400000 | 2.229100000 |
| C | -1.673100000 | -3.989300000 | 1.485700000 |
| C | -1.866800000 | -3.829500000 | 0.114200000 |
| C | -1.584500000 | -2.595600000 | -0.460300000 |
| N | -1.135700000 | -1.546600000 | 0.252200000 |
| N | 1.508900000 | -1.524200000 | -0.567900000 |
| C | 1.562200000 | -2.511500000 | -1.480500000 |
| C | 2.085100000 | -3.767900000 | -1.195200000 |
| C | 2.566600000 | -4.016900000 | 0.089200000 |
| C | 2.527000000 | -2.990700000 | 1.034200000 |
| Fe | 0.004800000 | 0.069000000 | -0.737700000 |
| O | -0.559100000 | -0.411400000 | -2.498700000 |
| O | -0.714700000 | 0.987400000 | -3.048300000 |
| H | 2.160900000 | 1.463500000 | -2.628200000 |
| H | 3.913400000 | 3.237600000 | -2.362100000 |
| H | 4.246900000 | 4.273000000 | -0.093900000 |
| H | 2.795600000 | 3.503600000 | 1.808500000 |
| H | -0.666800000 | 3.623900000 | 2.879300000 |
| H | -2.859100000 | 4.578100000 | 2.097500000 |
| H | -3.907900000 | 3.624800000 | 0.016300000 |
| H | -2.758400000 | 1.756700000 | -1.168800000 |
| H | -0.053900000 | -0.746000000 | 3.310000000 |
| H | -1.232700000 | 0.232000000 | 2.406400000 |
| H | 2.758000000 | 0.103900000 | 1.344000000 |
| H | 2.122400000 | -0.905800000 | 2.659300000 |
| H | -1.021000000 | -2.996100000 | 3.300400000 |
| H | -1.868700000 | -4.947500000 | 1.968200000 |
| H | -2.216600000 | -4.652200000 | -0.509300000 |
| H | -1.687400000 | -2.424500000 | -1.531600000 |
| H | 1.137400000 | -2.270000000 | -2.455800000 |
| H | 2.095500000 | -4.540400000 | -1.964800000 |
| H | 2.960600000 | -4.998100000 | 0.356100000 |
| H | 2.889900000 | -3.148200000 | 2.050200000 |
| H | -0.098700000 | 0.940100000 | -3.820100000 |
| H | 1.075100000 | 1.923200000 | 2.630100000 |
| C | -3.736600000 | -0.481400000 | -2.256300000 |
| H | -4.823600000 | -0.546500000 | -2.110900000 |
| H | -3.395500000 | -1.381700000 | -2.791600000 |
| O | -3.473200000 | 0.716000000 | -3.011200000 |
| H | -3.244700000 | -0.459900000 | -1.269500000 |
| H | -2.489600000 | 0.764500000 | -3.149000000 |

## {[(N4Py)$Fe^{II}$(OOH)(**MeOH**)]$^{+}$}-**b** (S=0)

59
symmetry c1

| | | | |
|---|---|---|---|
| C | 2.510422000 | 0.711811000 | -1.694761000 |
| C | 3.777019000 | 1.280763000 | -1.750739000 |
| C | 4.274331000 | 1.963627000 | -0.639097000 |
| C | 3.496967000 | 2.032762000 | 0.519397000 |
| C | 2.246611000 | 1.438728000 | 0.506738000 |
| N | 1.744704000 | 0.811318000 | -0.591309000 |
| C | 1.281211000 | 1.367589000 | 1.664870000 |
| C | 0.106271000 | 2.250146000 | 1.314039000 |
| C | -0.216254000 | 3.450911000 | 1.922256000 |
| C | -1.366245000 | 4.121582000 | 1.497520000 |
| C | -2.152393000 | 3.549524000 | 0.495817000 |
| C | -1.770536000 | 2.340549000 | -0.075033000 |
| N | -0.643843000 | 1.709189000 | 0.313121000 |
| N | 0.730260000 | -0.051773000 | 1.624839000 |
| C | -0.417739000 | -0.278084000 | 2.554012000 |
| C | 1.773104000 | -1.119897000 | 1.725146000 |
| C | 1.543884000 | -2.165906000 | 0.664298000 |
| C | -1.462689000 | -1.100318000 | 1.851971000 |
| C | -2.349688000 | -1.932240000 | 2.521770000 |
| C | -3.305127000 | -2.638950000 | 1.790693000 |
| C | -3.328152000 | -2.499285000 | 0.404354000 |
| C | -2.403826000 | -1.660635000 | -0.205636000 |
| N | -1.494617000 | -0.958349000 | 0.499397000 |
| N | 0.851443000 | -1.750581000 | -0.426861000 |
| C | 0.661396000 | -2.598589000 | -1.455609000 |
| C | 1.151663000 | -3.898454000 | -1.433852000 |
| C | 1.848152000 | -4.339417000 | -0.308959000 |
| C | 2.046922000 | -3.457759000 | 0.754071000 |
| Fe | 0.041633000 | -0.001014000 | -0.234716000 |
| O | -0.653160000 | -0.002974000 | -1.981552000 |
| O | -0.294330000 | 1.298924000 | -2.758918000 |
| H | 2.077230000 | 0.154755000 | -2.521460000 |
| H | 4.367000000 | 1.185430000 | -2.662211000 |
| H | 5.265361000 | 2.417302000 | -0.664979000 |
| H | 3.860059000 | 2.521282000 | 1.423202000 |
| H | 0.408485000 | 3.840754000 | 2.725536000 |
| H | -1.654829000 | 5.066148000 | 1.958855000 |
| H | -3.067977000 | 4.034125000 | 0.156134000 |
| H | -2.358949000 | 1.832683000 | -0.839389000 |
| H | -0.088835000 | -0.745623000 | 3.492275000 |
| H | -0.857176000 | 0.698361000 | 2.806341000 |
| H | 2.758269000 | -0.663564000 | 1.545521000 |
| H | 1.794077000 | -1.561760000 | 2.730844000 |
| H | -2.283558000 | -2.029645000 | 3.605380000 |
| H | -4.008893000 | -3.300593000 | 2.296208000 |
| H | -4.046042000 | -3.043075000 | -0.209535000 |
| H | -2.365998000 | -1.538601000 | -1.285358000 |
| H | 0.097568000 | -2.184570000 | -2.291696000 |
| H | 0.977145000 | -4.555319000 | -2.285957000 |
| H | 2.229300000 | -5.359729000 | -0.255408000 |
| H | 2.587073000 | -3.767152000 | 1.648715000 |
| H | 0.155082000 | 0.877666000 | -3.527715000 |
| H | 1.743400000 | 1.629568000 | 2.627859000 |
| C | -3.344236000 | 1.450564000 | -3.413859000 |
| H | -4.415477000 | 1.633747000 | -3.583541000 |
| H | -2.857728000 | 2.409318000 | -3.164671000 |
| O | -3.221246000 | 0.498882000 | -2.346976000 |
| H | -2.898177000 | 1.077762000 | -4.351759000 |
| H | -2.227913000 | 0.311527000 | -2.217434000 |

{[(N4Py)Fe$^{II}$(OOH)(**MeOH**)]$^{+}$}-**b** (S=1)

59
symmetry c1

| | | | |
|---|---|---|---|
| C | 2.330300000 | 0.787200000 | -1.831400000 |
| C | 3.556400000 | 1.439700000 | -1.938700000 |
| C | 4.087600000 | 2.086600000 | -0.827600000 |
| C | 3.386400000 | 2.072600000 | 0.382800000 |
| C | 2.181100000 | 1.398400000 | 0.425300000 |
| N | 1.675600000 | 0.764200000 | -0.660800000 |
| C | 1.251100000 | 1.302500000 | 1.612900000 |
| C | 0.049200000 | 2.155700000 | 1.275100000 |
| C | -0.270900000 | 3.367800000 | 1.858200000 |
| C | -1.419800000 | 4.028600000 | 1.416500000 |
| C | -2.203100000 | 3.447900000 | 0.422600000 |
| C | -1.825800000 | 2.224900000 | -0.124100000 |
| N | -0.711000000 | 1.605100000 | 0.294900000 |
| N | 0.742000000 | -0.122200000 | 1.616500000 |
| C | -0.381200000 | -0.366600000 | 2.572600000 |
| C | 1.821700000 | -1.151800000 | 1.712100000 |
| C | 1.572300000 | -2.244700000 | 0.711700000 |
| C | -1.450800000 | -1.180400000 | 1.900300000 |
| C | -2.361300000 | -1.960100000 | 2.601200000 |
| C | -3.356100000 | -2.637600000 | 1.899600000 |
| C | -3.408400000 | -2.521600000 | 0.512000000 |
| C | -2.463700000 | -1.738400000 | -0.134900000 |
| N | -1.512400000 | -1.076200000 | 0.549900000 |
| N | 0.850800000 | -1.890800000 | -0.378900000 |
| C | 0.632600000 | -2.779300000 | -1.365300000 |
| C | 1.137400000 | -4.070200000 | -1.297600000 |
| C | 1.868500000 | -4.453100000 | -0.174700000 |
| C | 2.088600000 | -3.527100000 | 0.842700000 |
| Fe | -0.012200000 | -0.138200000 | -0.283900000 |
| O | -0.593700000 | -0.155000000 | -1.870100000 |
| O | 0.120500000 | 2.467200000 | -2.910000000 |
| H | 1.849500000 | 0.269400000 | -2.657100000 |
| H | 4.080200000 | 1.437800000 | -2.893800000 |
| H | 5.047600000 | 2.598800000 | -0.894600000 |
| H | 3.770700000 | 2.564600000 | 1.275000000 |
| H | 0.357300000 | 3.776400000 | 2.648300000 |
| H | -1.704900000 | 4.983300000 | 1.858500000 |
| H | -3.111100000 | 3.932700000 | 0.065700000 |
| H | -2.405000000 | 1.711900000 | -0.894100000 |
| H | -0.017100000 | -0.849700000 | 3.488500000 |
| H | -0.815100000 | 0.602300000 | 2.861000000 |
| H | 2.782200000 | -0.675400000 | 1.465000000 |
| H | 1.900700000 | -1.543000000 | 2.734500000 |
| H | -2.282500000 | -2.034800000 | 3.685300000 |
| H | -4.077300000 | -3.258300000 | 2.431500000 |
| H | -4.164400000 | -3.041700000 | -0.074900000 |
| H | -2.436200000 | -1.627100000 | -1.215900000 |
| H | 0.035300000 | -2.414800000 | -2.199200000 |
| H | 0.943300000 | -4.765100000 | -2.113700000 |
| H | 2.260600000 | -5.466500000 | -0.087200000 |
| H | 2.654900000 | -3.791100000 | 1.735200000 |
| H | -0.076700000 | 1.815000000 | -2.213500000 |
| H | 1.728500000 | 1.588500000 | 2.560000000 |
| C | -2.944100000 | 1.558500000 | -3.558600000 |
| H | -3.783100000 | 2.255300000 | -3.414200000 |
| H | -1.984500000 | 2.103500000 | -3.461400000 |
| O | -3.036500000 | 0.528700000 | -2.550600000 |
| H | -3.014200000 | 1.127100000 | -4.573200000 |
| H | -2.089600000 | 0.242200000 | -2.352800000 |

{[(N4Py)Fe$^{II}$(OOH)(**MeOH**)]$^{+}$}-**b** (S=2)

59
symmetry c1

| | | | |
|---|---|---|---|
| C | 2.742000000 | 1.125500000 | -1.761600000 |
| C | 3.951900000 | 1.805700000 | -1.674800000 |
| C | 4.323600000 | 2.369500000 | -0.454300000 |
| C | 3.472100000 | 2.229800000 | 0.642200000 |
| C | 2.280800000 | 1.538000000 | 0.472600000 |
| N | 1.912400000 | 0.997800000 | -0.711700000 |
| C | 1.259200000 | 1.355700000 | 1.580300000 |
| C | 0.113700000 | 2.301000000 | 1.260800000 |
| C | -0.119000000 | 3.479500000 | 1.955700000 |
| C | -1.187900000 | 4.285900000 | 1.561300000 |
| C | -1.986500000 | 3.875000000 | 0.493800000 |
| C | -1.692100000 | 2.677500000 | -0.151100000 |
| N | -0.652100000 | 1.906100000 | 0.216900000 |
| N | 0.738600000 | -0.034500000 | 1.494200000 |
| C | -0.402700000 | -0.316000000 | 2.388500000 |
| C | 1.775800000 | -1.086000000 | 1.572000000 |
| C | 1.409800000 | -2.244500000 | 0.675200000 |
| C | -1.347400000 | -1.294200000 | 1.733100000 |
| C | -1.938100000 | -2.341200000 | 2.431000000 |
| C | -2.829300000 | -3.182700000 | 1.764600000 |
| C | -3.104300000 | -2.940800000 | 0.419500000 |
| C | -2.463100000 | -1.880500000 | -0.212600000 |
| N | -1.596800000 | -1.071700000 | 0.422900000 |
| N | 0.892600000 | -1.908300000 | -0.528500000 |
| C | 0.569800000 | -2.890200000 | -1.391000000 |
| C | 0.741200000 | -4.236600000 | -1.090300000 |
| C | 1.260700000 | -4.584100000 | 0.156200000 |
| C | 1.600700000 | -3.569000000 | 1.051000000 |
| Fe | -0.033800000 | 0.054700000 | -0.677700000 |
| O | -0.950700000 | -0.235400000 | -2.359000000 |
| O | -0.300900000 | 0.943000000 | -3.035700000 |
| H | 2.418100000 | 0.656900000 | -2.689400000 |
| H | 4.593400000 | 1.882500000 | -2.552600000 |
| H | 5.270000000 | 2.901900000 | -0.353500000 |
| H | 3.730200000 | 2.642600000 | 1.617300000 |
| H | 0.518100000 | 3.753500000 | 2.796700000 |
| H | -1.401400000 | 5.215300000 | 2.090300000 |
| H | -2.839400000 | 4.469500000 | 0.165900000 |
| H | -2.306400000 | 2.286700000 | -0.964300000 |
| H | -0.066300000 | -0.684200000 | 3.371300000 |
| H | -0.956200000 | 0.620400000 | 2.556000000 |
| H | 2.727600000 | -0.669500000 | 1.209400000 |
| H | 1.935300000 | -1.420800000 | 2.609400000 |
| H | -1.693700000 | -2.500000000 | 3.481800000 |
| H | -3.295100000 | -4.019200000 | 2.287000000 |
| H | -3.793900000 | -3.571600000 | -0.141700000 |
| H | -2.626000000 | -1.671600000 | -1.270000000 |
| H | 0.135200000 | -2.559000000 | -2.335000000 |
| H | 0.458300000 | -4.996400000 | -1.819000000 |
| H | 1.389800000 | -5.630900000 | 0.433500000 |
| H | 2.000900000 | -3.801000000 | 2.038300000 |
| H | 0.285600000 | 0.461000000 | -3.668100000 |
| H | 1.700100000 | 1.591000000 | 2.563200000 |
| C | -3.451500000 | 1.686600000 | -3.431500000 |
| H | -4.445300000 | 2.154800000 | -3.457300000 |
| H | -2.687300000 | 2.482000000 | -3.393100000 |
| O | -3.394400000 | 0.839000000 | -2.270700000 |
| H | -3.307400000 | 1.114500000 | -4.364100000 |
| H | -2.488000000 | 0.400600000 | -2.291200000 |

| {[(N4Py)Fe$^{II}$(OOH)(**MeOH**)]$^{+}$}-**c** (S=0) | | | |
|---|---|---|---|
| 59 | | | |
| symmetry c1 | | | |
| C | 2.641139000 | -0.876351000 | -0.239435000 |
| C | 3.581164000 | -1.556600000 | -1.003107000 |
| C | 3.152945000 | -2.392570000 | -2.036218000 |
| C | 1.784064000 | -2.498605000 | -2.294364000 |
| C | 0.904934000 | -1.782850000 | -1.498588000 |
| N | 1.317992000 | -1.000369000 | -0.462602000 |
| C | -0.591406000 | -1.713040000 | -1.675305000 |
| C | -1.210475000 | -2.409966000 | -0.485154000 |
| C | -1.894630000 | -3.613064000 | -0.503808000 |
| C | -2.416514000 | -4.098297000 | 0.699273000 |
| C | -2.238064000 | -3.354163000 | 1.868393000 |
| C | -1.540682000 | -2.153194000 | 1.817866000 |
| N | -1.023616000 | -1.695451000 | 0.660397000 |
| N | -0.926103000 | -0.236044000 | -1.492401000 |
| C | -2.391752000 | 0.039739000 | -1.432141000 |
| C | -0.199987000 | 0.683102000 | -2.424511000 |
| C | 0.441405000 | 1.814667000 | -1.657820000 |
| C | -2.681927000 | 0.985777000 | -0.299052000 |
| C | -3.812799000 | 1.790070000 | -0.248666000 |
| C | -4.036260000 | 2.578672000 | 0.880911000 |
| C | -3.116028000 | 2.534478000 | 1.927796000 |
| C | -1.997446000 | 1.718851000 | 1.809258000 |
| N | -1.781292000 | 0.959993000 | 0.717139000 |
| N | 0.604257000 | 1.611060000 | -0.326960000 |
| C | 1.210229000 | 2.552798000 | 0.423962000 |
| C | 1.702811000 | 3.725753000 | -0.130860000 |
| C | 1.543121000 | 3.943658000 | -1.500788000 |
| C | 0.896564000 | 2.977048000 | -2.270875000 |
| Fe | -0.156027000 | -0.030187000 | 0.333340000 |
| O | 0.418071000 | 0.265383000 | 2.101250000 |
| O | 1.493015000 | -0.699291000 | 2.610872000 |
| H | 2.913269000 | -0.206869000 | 0.568184000 |
| H | 4.641860000 | -1.426791000 | -0.788370000 |
| H | 3.874037000 | -2.935271000 | -2.647730000 |
| H | 1.403509000 | -3.106551000 | -3.114771000 |
| H | -2.031765000 | -4.149527000 | -1.442673000 |
| H | -2.968796000 | -5.037851000 | 0.719602000 |
| H | -2.640827000 | -3.701287000 | 2.820181000 |
| H | -1.359671000 | -1.516641000 | 2.683660000 |
| H | -2.770316000 | 0.418796000 | -2.391581000 |
| H | -2.913217000 | -0.907010000 | -1.223030000 |
| H | 0.595766000 | 0.115775000 | -2.930167000 |
| H | -0.872039000 | 1.064812000 | -3.205928000 |
| H | -4.508572000 | 1.796730000 | -1.087852000 |
| H | -4.915140000 | 3.221397000 | 0.938602000 |
| H | -3.251798000 | 3.134178000 | 2.827921000 |
| H | -1.230530000 | 1.638968000 | 2.580312000 |
| H | 1.265186000 | 2.305167000 | 1.485442000 |
| H | 2.194964000 | 4.461183000 | 0.505376000 |
| H | 1.910417000 | 4.860025000 | -1.963898000 |
| H | 0.747495000 | 3.117959000 | -3.341688000 |
| H | 2.272030000 | -0.085324000 | 2.529635000 |
| H | -0.929788000 | -2.124632000 | -2.637976000 |
| O | 3.649125000 | 1.101701000 | 2.334844000 |
| H | 5.480386000 | 0.520343000 | 3.141504000 |
| H | 3.559764000 | 1.768599000 | 1.621482000 |
| C | 4.945319000 | 0.457317000 | 2.184794000 |
| H | 4.799611000 | -0.601302000 | 1.923992000 |
| H | 5.538495000 | 0.944633000 | 1.402282000 |

| {[(N4Py)Fe$^{II}$(OOH)(**MeOH**)]$^{+}$}-**c** (S=1) | | | |
|---|---|---|---|
| 59 | | | |
| symmetry c1 | | | |
| C | 2.192300000 | 2.534700000 | 0.579800000 |
| C | 3.246100000 | 3.246400000 | 0.009900000 |
| C | 3.798900000 | 2.790600000 | -1.184600000 |
| C | 3.309200000 | 1.614700000 | -1.756900000 |
| C | 2.269800000 | 0.958300000 | -1.112500000 |
| N | 1.699800000 | 1.421900000 | 0.018600000 |
| C | 1.769800000 | -0.409400000 | -1.540200000 |
| C | 2.334700000 | -1.379600000 | -0.519100000 |
| C | 3.434200000 | -2.193900000 | -0.731400000 |
| C | 3.884400000 | -2.993500000 | 0.320600000 |
| C | 3.217700000 | -2.948000000 | 1.543500000 |
| C | 2.118100000 | -2.109200000 | 1.686700000 |
| N | 1.687700000 | -1.337900000 | 0.674900000 |
| N | 0.279200000 | -0.476300000 | -1.379400000 |
| C | -0.252500000 | -1.825300000 | -1.755800000 |
| C | -0.476600000 | 0.613200000 | -2.069200000 |
| C | -1.727200000 | 0.877600000 | -1.280400000 |
| C | -1.452200000 | -2.168600000 | -0.914700000 |
| C | -2.603600000 | -2.755700000 | -1.424700000 |
| C | -3.648300000 | -3.050200000 | -0.547200000 |
| C | -3.502700000 | -2.754900000 | 0.807100000 |
| C | -2.323800000 | -2.153300000 | 1.236600000 |
| N | -1.320700000 | -1.866400000 | 0.393100000 |
| N | -1.555900000 | 0.829600000 | 0.073400000 |
| C | -2.617800000 | 1.036300000 | 0.884400000 |
| C | -3.881800000 | 1.293500000 | 0.375500000 |
| C | -4.063700000 | 1.343000000 | -1.004900000 |
| C | -2.966000000 | 1.139900000 | -1.842400000 |
| Fe | 0.073700000 | -0.134400000 | 0.699700000 |
| O | -0.049700000 | 0.075000000 | 2.327000000 |
| O | -0.517700000 | 2.008100000 | 3.053100000 |
| H | 1.716700000 | 2.856700000 | 1.505400000 |
| H | 3.618100000 | 4.147600000 | 0.497200000 |
| H | 4.610300000 | 3.338600000 | -1.664700000 |
| H | 3.728500000 | 1.213900000 | -2.679600000 |
| H | 3.925000000 | -2.203400000 | -1.704100000 |
| H | 4.744000000 | -3.649400000 | 0.182300000 |
| H | 3.540200000 | -3.559000000 | 2.385900000 |
| H | 1.551400000 | -2.031200000 | 2.613500000 |
| H | -0.474500000 | -1.862000000 | -2.831800000 |
| H | 0.532000000 | -2.567200000 | -1.545600000 |
| H | 0.145200000 | 1.520800000 | -2.071900000 |
| H | -0.693000000 | 0.349900000 | -3.114200000 |
| H | -2.685900000 | -2.965700000 | -2.491100000 |
| H | -4.570700000 | -3.495800000 | -0.921300000 |
| H | -4.297700000 | -2.966700000 | 1.521800000 |
| H | -2.167400000 | -1.857300000 | 2.274500000 |
| H | -2.390700000 | 0.998000000 | 1.950200000 |
| H | -4.713200000 | 1.448100000 | 1.062600000 |
| H | -5.049600000 | 1.533800000 | -1.429100000 |
| H | -3.069800000 | 1.166400000 | -2.926800000 |
| H | -0.394000000 | 2.447700000 | 2.180200000 |
| H | 2.086800000 | -0.655200000 | -2.564600000 |
| O | -0.482700000 | 3.608000000 | 0.338700000 |
| H | -2.421800000 | 3.785100000 | 1.158000000 |
| H | -0.526600000 | 2.648600000 | 0.139300000 |
| C | -1.838500000 | 4.112700000 | 0.284100000 |
| H | -1.769200000 | 5.207400000 | 0.291300000 |
| H | -2.348500000 | 3.791200000 | -0.636600000 |

| {[(N4Py)Fe$^{II}$(OOH)(**MeOH**)]$^{+}$}-**c** (S=2) | | | |
|---|---|---|---|
| 59 | | | |
| symmetry c1 | | | |
| C | 2.887800000 | -2.063000000 | 0.533800000 |
| C | 3.691200000 | -2.907600000 | -0.221400000 |
| C | 3.262300000 | -3.303600000 | -1.488600000 |
| C | 2.020800000 | -2.861500000 | -1.943500000 |
| C | 1.271300000 | -2.027600000 | -1.125700000 |
| N | 1.699500000 | -1.604400000 | 0.089900000 |
| C | -0.134900000 | -1.586800000 | -1.468200000 |
| C | -1.061100000 | -2.383800000 | -0.563600000 |
| C | -1.864800000 | -3.417600000 | -1.023200000 |
| C | -2.657700000 | -4.105700000 | -0.104800000 |
| C | -2.617300000 | -3.731200000 | 1.239000000 |
| C | -1.789800000 | -2.682700000 | 1.623500000 |
| N | -1.017100000 | -2.021200000 | 0.742000000 |
| N | -0.270400000 | -0.144700000 | -1.110900000 |
| C | -1.666200000 | 0.349700000 | -1.246600000 |
| C | 0.688600000 | 0.714900000 | -1.866100000 |
| C | 0.612400000 | 2.096000000 | -1.279600000 |
| C | -2.027900000 | 1.281800000 | -0.114800000 |
| C | -2.885500000 | 2.362100000 | -0.292700000 |
| C | -3.218700000 | 3.150600000 | 0.807200000 |
| C | -2.687400000 | 2.826600000 | 2.054300000 |
| C | -1.827500000 | 1.738900000 | 2.150200000 |
| N | -1.493900000 | 0.983200000 | 1.087100000 |
| N | 0.998900000 | 2.189900000 | 0.010200000 |
| C | 0.733600000 | 3.323200000 | 0.678100000 |
| C | 0.090700000 | 4.410300000 | 0.091500000 |
| C | -0.243000000 | 4.337400000 | -1.258300000 |
| C | 0.021700000 | 3.158900000 | -1.955900000 |
| Fe | 0.251800000 | -0.356300000 | 1.086000000 |
| O | 0.741200000 | 0.358100000 | 2.762900000 |
| O | 2.048500000 | -0.340700000 | 3.005000000 |
| H | 3.183400000 | -1.724400000 | 1.523900000 |
| H | 4.647300000 | -3.241500000 | 0.181900000 |
| H | 3.881900000 | -3.949700000 | -2.111600000 |
| H | 1.634200000 | -3.156700000 | -2.919100000 |
| H | -1.876700000 | -3.669200000 | -2.083800000 |
| H | -3.305500000 | -4.917800000 | -0.435900000 |
| H | -3.225900000 | -4.241000000 | 1.985500000 |
| H | -1.730300000 | -2.346200000 | 2.658500000 |
| H | -1.829700000 | 0.826900000 | -2.225400000 |
| H | -2.347700000 | -0.512600000 | -1.190800000 |
| H | 1.696300000 | 0.299000000 | -1.732100000 |
| H | 0.449100000 | 0.717800000 | -2.942500000 |
| H | -3.271000000 | 2.591500000 | -1.286400000 |
| H | -3.875500000 | 4.013200000 | 0.688300000 |
| H | -2.919500000 | 3.416400000 | 2.941200000 |
| H | -1.359400000 | 1.459300000 | 3.094200000 |
| H | 1.038000000 | 3.339600000 | 1.726000000 |
| H | -0.137200000 | 5.295000000 | 0.685500000 |
| H | -0.734400000 | 5.174500000 | -1.756000000 |
| H | -0.270300000 | 3.045900000 | -3.000000000 |
| H | 2.637200000 | 0.226200000 | 2.399900000 |
| H | -0.363500000 | -1.781600000 | -2.529300000 |
| O | 3.242800000 | 1.247000000 | 1.229200000 |
| H | 5.022600000 | 0.375200000 | 0.657500000 |
| H | 2.382700000 | 1.508500000 | 0.768600000 |
| C | 4.174900000 | 0.889500000 | 0.187500000 |
| H | 3.718900000 | 0.209000000 | -0.546800000 |
| H | 4.538000000 | 1.789800000 | -0.332600000 |

| {[(N4Py)Fe$^{II}$(OOH)(**MeOH**)$_2$]$^{+}$}-**a** (S=0) | | | |
|---|---|---|---|
| symmetry c1 | | | |
| C | 2.745000000 | -0.602900000 | -0.567000000 |
| C | 3.748400000 | -1.094700000 | -1.391500000 |
| C | 3.409600000 | -1.673300000 | -2.617400000 |
| C | 2.065600000 | -1.719600000 | -2.999400000 |
| C | 1.117200000 | -1.204900000 | -2.131900000 |
| N | 1.446900000 | -0.679200000 | -0.919600000 |
| C | -0.370600000 | -1.110600000 | -2.380700000 |
| C | -1.036600000 | -2.074800000 | -1.426500000 |
| C | -1.689700000 | -3.244400000 | -1.774400000 |
| C | -2.261400000 | -4.013000000 | -0.756200000 |
| C | -2.157400000 | -3.573800000 | 0.565000000 |
| C | -1.488400000 | -2.387500000 | 0.845200000 |
| N | -0.928400000 | -1.651800000 | -0.135100000 |
| N | -0.761500000 | 0.273200000 | -1.876500000 |
| C | -2.236900000 | 0.502000000 | -1.816600000 |
| C | -0.028700000 | 1.398300000 | -2.534600000 |
| C | 0.498800000 | 2.353100000 | -1.493800000 |
| C | -2.590900000 | 1.161700000 | -0.510400000 |
| C | -3.739300000 | 1.920900000 | -0.324600000 |
| C | -4.010600000 | 2.443300000 | 0.940900000 |
| C | -3.114400000 | 2.197300000 | 1.981000000 |
| C | -1.976200000 | 1.443400000 | 1.724800000 |
| N | -1.723500000 | 0.927000000 | 0.507000000 |
| N | 0.670500000 | 1.829800000 | -0.253500000 |
| C | 1.216600000 | 2.584800000 | 0.718800000 |
| C | 1.610500000 | 3.897200000 | 0.492300000 |
| C | 1.418400000 | 4.451400000 | -0.773700000 |
| C | 0.854300000 | 3.665900000 | -1.779700000 |
| Fe | -0.069300000 | 0.056700000 | -0.020800000 |
| O | 0.488400000 | -0.044600000 | 1.749400000 |
| O | 1.255500000 | -1.358400000 | 2.077900000 |
| H | 2.932800000 | -0.135000000 | 0.395300000 |
| H | 4.789000000 | -1.022400000 | -1.076400000 |
| H | 4.183500000 | -2.064200000 | -3.278200000 |
| H | 1.760000000 | -2.127900000 | -3.962400000 |
| H | -1.763500000 | -3.537800000 | -2.821300000 |
| H | -2.790800000 | -4.935800000 | -0.994600000 |
| H | -2.594500000 | -4.146900000 | 1.383000000 |
| H | -1.361800000 | -2.003000000 | 1.854000000 |
| H | -2.587700000 | 1.086500000 | -2.678200000 |
| H | -2.742300000 | -0.475000000 | -1.853500000 |
| H | 0.832100000 | 0.982300000 | -3.079800000 |
| H | -0.663800000 | 1.911700000 | -3.269500000 |
| H | -4.410800000 | 2.100600000 | -1.164300000 |
| H | -4.905300000 | 3.044300000 | 1.107400000 |
| H | -3.285200000 | 2.594200000 | 2.981600000 |
| H | -1.220500000 | 1.221000000 | 2.476300000 |
| H | 1.315800000 | 2.077100000 | 1.679100000 |
| H | 2.051500000 | 4.477200000 | 1.303000000 |
| H | 1.701600000 | 5.484800000 | -0.976600000 |
| H | 0.691800000 | 4.064600000 | -2.781000000 |
| H | 2.127400000 | -0.938300000 | 2.326700000 |
| H | -0.643100000 | -1.288400000 | -3.431600000 |
| O | 3.624800000 | -0.160900000 | 2.746900000 |
| H | 5.695700000 | -0.516900000 | 2.904700000 |
| H | 3.636900000 | 0.814600000 | 2.860200000 |
| O | -0.372000000 | -2.028500000 | 4.087400000 |
| H | -1.958700000 | -0.720300000 | 3.599800000 |
| H | 0.266300000 | -1.738600000 | 3.360500000 |
| C | -1.215100000 | -0.909900000 | 4.394200000 |
| H | -1.757500000 | -1.136700000 | 5.324300000 |
| H | -0.634300000 | 0.015700000 | 4.542100000 |
| C | 4.885000000 | -0.570100000 | 2.162900000 |
| H | 4.754800000 | -1.609400000 | 1.839700000 |
| H | 5.144200000 | 0.050000000 | 1.293200000 |

$\{[(N4Py)Fe^{II}(OOH)(\mathbf{MeOH})_2]^+\}$-**a** (S=1)

| | | | |
|---|---|---|---|
| symmetry c1 | | | |
| C | 1.700700000 | 2.414000000 | 0.632900000 |
| C | 2.650400000 | 3.391200000 | 0.359700000 |
| C | 3.262400000 | 3.416200000 | -0.891600000 |
| C | 2.912100000 | 2.461200000 | -1.848400000 |
| C | 1.960400000 | 1.516100000 | -1.510600000 |
| N | 1.373300000 | 1.497600000 | -0.288300000 |
| C | 1.460200000 | 0.395400000 | -2.394300000 |
| C | 2.010800000 | -0.884700000 | -1.810500000 |
| C | 2.988500000 | -1.679800000 | -2.377400000 |
| C | 3.411300000 | -2.806600000 | -1.666000000 |
| C | 2.855200000 | -3.074800000 | -0.418700000 |
| C | 1.865200000 | -2.239100000 | 0.101100000 |
| N | 1.457900000 | -1.174400000 | -0.605100000 |
| N | -0.030300000 | 0.335700000 | -2.155600000 |
| C | -0.688400000 | -0.858900000 | -2.769000000 |
| C | -0.740800000 | 1.615000000 | -2.457200000 |
| C | -1.719300000 | 1.922900000 | -1.361200000 |
| C | -1.633600000 | -1.479900000 | -1.782000000 |
| C | -2.700100000 | -2.286400000 | -2.156300000 |
| C | -3.496500000 | -2.859900000 | -1.167400000 |
| C | -3.212500000 | -2.598200000 | 0.171600000 |
| C | -2.141400000 | -1.774400000 | 0.483900000 |
| N | -1.361900000 | -1.243700000 | -0.476300000 |
| N | -1.442800000 | 1.387900000 | -0.146600000 |
| C | -2.261800000 | 1.633400000 | 0.894200000 |
| C | -3.369400000 | 2.459200000 | 0.768700000 |
| C | -3.656400000 | 3.024300000 | -0.472100000 |
| C | -2.823300000 | 2.743800000 | -1.551900000 |
| Fe | 0.028300000 | 0.081200000 | -0.119100000 |
| O | 0.120800000 | -0.128500000 | 1.540100000 |
| O | 1.291400000 | -0.799600000 | 4.821500000 |
| H | 1.186300000 | 2.335100000 | 1.585900000 |
| H | 2.899300000 | 4.124100000 | 1.126200000 |
| H | 4.003900000 | 4.179400000 | -1.127500000 |
| H | 3.358600000 | 2.455700000 | -2.841800000 |
| H | 3.402300000 | -1.426900000 | -3.352400000 |
| H | 4.173700000 | -3.462500000 | -2.087100000 |
| H | 3.182400000 | -3.932400000 | 0.169300000 |
| H | 1.463700000 | -2.355600000 | 1.130000000 |
| H | -1.196700000 | -0.586600000 | -3.703000000 |
| H | 0.087400000 | -1.598400000 | -3.017000000 |
| H | -0.000400000 | 2.428000000 | -2.496500000 |
| H | -1.231100000 | 1.568700000 | -3.438400000 |
| H | -2.900900000 | -2.455800000 | -3.213700000 |
| H | -4.339600000 | -3.494000000 | -1.441800000 |
| H | -3.819100000 | -3.014800000 | 0.974700000 |
| H | -1.876600000 | -1.508000000 | 1.503200000 |
| H | -2.012000000 | 1.141500000 | 1.831400000 |
| H | -4.003800000 | 2.637400000 | 1.636400000 |
| H | -4.528000000 | 3.666100000 | -0.602300000 |
| H | -3.022700000 | 3.153000000 | -2.541800000 |
| H | 0.556200000 | -0.183200000 | 4.583000000 |
| H | 1.718100000 | 0.535000000 | -3.452800000 |
| O | -0.790100000 | 0.853100000 | 3.778100000 |
| H | -0.754900000 | 2.670600000 | 4.743800000 |
| H | -0.424100000 | 0.459300000 | 2.921200000 |
| O | 1.210400000 | -2.526800000 | 2.919300000 |
| H | -0.405900000 | -3.351300000 | 1.878800000 |
| H | 1.259500000 | -1.493000000 | 4.034400000 |
| C | -0.084900000 | -3.033400000 | 2.899600000 |
| H | -0.206600000 | -3.933200000 | 3.549100000 |
| H | -0.847000000 | -2.298900000 | 3.250700000 |
| C | -0.335000000 | 2.217100000 | 3.836100000 |
| H | 0.765200000 | 2.273900000 | 3.891800000 |
| H | -0.682500000 | 2.800500000 | 2.965700000 |

$\{[(N4Py)Fe^{II}(OOH)(\mathbf{MeOH})_2]^+\}$-**a** (S=2)

| | | | |
|---|---|---|---|
| symmetry c1 | | | |
| C | 3.063224000 | -1.888968000 | -0.318537000 |
| C | 3.849900000 | -2.542311000 | -1.258988000 |
| C | 3.376108000 | -2.681003000 | -2.563039000 |
| C | 2.111295000 | -2.182817000 | -2.872774000 |
| C | 1.383006000 | -1.550583000 | -1.875111000 |
| N | 1.853642000 | -1.376916000 | -0.615831000 |
| C | -0.047607000 | -1.092197000 | -2.066987000 |
| C | -0.902025000 | -2.100518000 | -1.317209000 |
| C | -1.667786000 | -3.064807000 | -1.955637000 |
| C | -2.356586000 | -3.995521000 | -1.177418000 |
| C | -2.235771000 | -3.933084000 | 0.210919000 |
| C | -1.453900000 | -2.935149000 | 0.781349000 |
| N | -0.805666000 | -2.019096000 | 0.034342000 |
| N | -0.209897000 | 0.230931000 | -1.402421000 |
| C | -1.624363000 | 0.682074000 | -1.384068000 |
| C | 0.705278000 | 1.269879000 | -1.960286000 |
| C | 0.769830000 | 2.405962000 | -0.977204000 |
| C | -1.955036000 | 1.403291000 | -0.100188000 |
| C | -2.956129000 | 2.368733000 | -0.039581000 |
| C | -3.217755000 | 2.994917000 | 1.176370000 |
| C | -2.455494000 | 2.647886000 | 2.291241000 |
| C | -1.473657000 | 1.678312000 | 2.148572000 |
| N | -1.237941000 | 1.046232000 | 0.982566000 |
| N | 1.391716000 | 2.130338000 | 0.190385000 |
| C | 1.314900000 | 3.033006000 | 1.183042000 |
| C | 0.607104000 | 4.225275000 | 1.069157000 |
| C | -0.015068000 | 4.516407000 | -0.141032000 |
| C | 0.080349000 | 3.595931000 | -1.184964000 |
| Fe | 0.408325000 | -0.402007000 | 0.665782000 |
| O | 0.822873000 | -0.164959000 | 2.518493000 |
| O | 2.024942000 | -1.060997000 | 2.573337000 |
| H | 3.395795000 | -1.759092000 | 0.706937000 |
| H | 4.825493000 | -2.930390000 | -0.966728000 |
| H | 3.978686000 | -3.174081000 | -3.326110000 |
| H | 1.689774000 | -2.284409000 | -3.872813000 |
| H | -1.725395000 | -3.080998000 | -3.044018000 |
| H | -2.975646000 | -4.758583000 | -1.649529000 |
| H | -2.744311000 | -4.650917000 | 0.854411000 |
| H | -1.317345000 | -2.846520000 | 1.860495000 |
| H | -1.853328000 | 1.310959000 | -2.259420000 |
| H | -2.272523000 | -0.205218000 | -1.445559000 |
| H | 1.701822000 | 0.821365000 | -2.071433000 |
| H | 0.356778000 | 1.606419000 | -2.950209000 |
| H | -3.506951000 | 2.637391000 | -0.941466000 |
| H | -3.992213000 | 3.759596000 | 1.247579000 |
| H | -2.606079000 | 3.131214000 | 3.256461000 |
| H | -0.831703000 | 1.389983000 | 2.980811000 |
| H | 1.820899000 | 2.762407000 | 2.110906000 |
| H | 0.542071000 | 4.903243000 | 1.919825000 |
| H | -0.586546000 | 5.436648000 | -0.267278000 |
| H | -0.418725000 | 3.775022000 | -2.137401000 |
| H | 2.711425000 | -0.409550000 | 2.200919000 |
| H | -0.314987000 | -1.064949000 | -3.135951000 |
| O | 3.483069000 | 0.804502000 | 1.349184000 |
| H | 5.423488000 | 0.169395000 | 1.075437000 |
| H | 2.743653000 | 1.177972000 | 0.779582000 |
| O | -1.051755000 | -1.712669000 | 3.747171000 |
| H | -2.503513000 | -0.889572000 | 2.448709000 |
| H | -0.361899000 | -1.110636000 | 3.330655000 |
| C | -2.343646000 | -1.124990000 | 3.514931000 |
| H | -3.102127000 | -1.857210000 | 3.825471000 |
| H | -2.487885000 | -0.199368000 | 4.098321000 |
| C | 4.681718000 | 0.779481000 | 0.545945000 |
| H | 4.493713000 | 0.333424000 | -0.442404000 |
| H | 5.077105000 | 1.798510000 | 0.413269000 |

| {[(N4Py)Fe$^{II}$(OOH)(**MeOH**)$_2$]$^+$}-**b** (S=0) | | | |
|---|---|---|---|
| symmetry c1 | | | |
| C | -2.726000000 | 0.877000000 | -0.323200000 |
| C | -3.724600000 | 1.487200000 | -1.072300000 |
| C | -3.385400000 | 2.196900000 | -2.224800000 |
| C | -2.045900000 | 2.243400000 | -2.614800000 |
| C | -1.106800000 | 1.599300000 | -1.826500000 |
| N | -1.425700000 | 0.949400000 | -0.673200000 |
| C | 0.357200000 | 1.464100000 | -2.148100000 |
| C | 1.128300000 | 2.268200000 | -1.131500000 |
| C | 1.836900000 | 3.428800000 | -1.391400000 |
| C | 2.560700000 | 4.012900000 | -0.348800000 |
| C | 2.564700000 | 3.390300000 | 0.900400000 |
| C | 1.827900000 | 2.227400000 | 1.095100000 |
| N | 1.089400000 | 1.688200000 | 0.102100000 |
| N | 0.683100000 | 0.008400000 | -1.827800000 |
| C | 2.143500000 | -0.297900000 | -1.897500000 |
| C | -0.156600000 | -0.981300000 | -2.576500000 |
| C | -0.730900000 | -2.003900000 | -1.629300000 |
| C | 2.544400000 | -1.120200000 | -0.705000000 |
| C | 3.672800000 | -1.929500000 | -0.694600000 |
| C | 4.008200000 | -2.615800000 | 0.472100000 |
| C | 3.187900000 | -2.479300000 | 1.591000000 |
| C | 2.066300000 | -1.663800000 | 1.511700000 |
| N | 1.750600000 | -0.984500000 | 0.390300000 |
| N | -0.732000000 | -1.661100000 | -0.317400000 |
| C | -1.233500000 | -2.520200000 | 0.593800000 |
| C | -1.801700000 | -3.731200000 | 0.224400000 |
| C | -1.827600000 | -4.081300000 | -1.127000000 |
| C | -1.271100000 | -3.209900000 | -2.062200000 |
| Fe | 0.118900000 | 0.024000000 | 0.073900000 |
| O | -0.302200000 | -0.076700000 | 1.920300000 |
| O | -1.464600000 | 0.783200000 | 2.415700000 |
| H | -2.937200000 | 0.312200000 | 0.576900000 |
| H | -4.762300000 | 1.401200000 | -0.751000000 |
| H | -4.153500000 | 2.684000000 | -2.825700000 |
| H | -1.734400000 | 2.745600000 | -3.530200000 |
| H | 1.841900000 | 3.850800000 | -2.396400000 |
| H | 3.134500000 | 4.924500000 | -0.518900000 |
| H | 3.143700000 | 3.799000000 | 1.728800000 |
| H | 1.821600000 | 1.684200000 | 2.040200000 |
| H | 2.403900000 | -0.794500000 | -2.842200000 |
| H | 2.699200000 | 0.651100000 | -1.861700000 |
| H | -0.989000000 | -0.446400000 | -3.057700000 |
| H | 0.423900000 | -1.463800000 | -3.374700000 |
| H | 4.279800000 | -2.018200000 | -1.595700000 |
| H | 4.889800000 | -3.256600000 | 0.501300000 |
| H | 3.403900000 | -3.005100000 | 2.520900000 |
| H | 1.380400000 | -1.539500000 | 2.347000000 |
| H | -1.152000000 | -2.179100000 | 1.625800000 |
| H | -2.206700000 | -4.393800000 | 0.989200000 |
| H | -2.262300000 | -5.028300000 | -1.447700000 |
| H | -1.255300000 | -3.458800000 | -3.123600000 |
| H | -2.164400000 | 0.073300000 | 2.424100000 |
| H | 0.595000000 | 1.744900000 | -3.185100000 |
| O | -3.473600000 | -1.155500000 | 2.376500000 |
| H | -4.673200000 | 0.177500000 | 3.367800000 |
| H | -3.530900000 | -1.774800000 | 1.615200000 |
| O | 1.302400000 | 0.661100000 | 3.837900000 |
| H | -0.269700000 | 1.601000000 | 4.884900000 |
| H | 0.669900000 | 0.383700000 | 3.081600000 |
| C | 0.722800000 | 1.818600000 | 4.456200000 |
| H | 1.395100000 | 2.149400000 | 5.261400000 |
| H | 0.605100000 | 2.652100000 | 3.739400000 |
| C | -4.773600000 | -0.531800000 | 2.538500000 |
| H | -5.073200000 | 0.011900000 | 1.631100000 |
| H | -5.538400000 | -1.281100000 | 2.788400000 |

| {[(N4Py)Fe$^{II}$(OOH)(**MeOH**)$_2$]$^+$}-**b** (S=1) | | | |
|---|---|---|---|
| symmetry c1 | | | |
| C | -2.031300000 | -1.847700000 | 1.785600000 |
| C | -3.049000000 | -2.799100000 | 1.744700000 |
| C | -3.473700000 | -3.280700000 | 0.508600000 |
| C | -2.876600000 | -2.796200000 | -0.658500000 |
| C | -1.883800000 | -1.837200000 | -0.526900000 |
| N | -1.467600000 | -1.374600000 | 0.669300000 |
| C | -1.198900000 | -1.158700000 | -1.698300000 |
| C | -1.712600000 | 0.266200000 | -1.747300000 |
| C | -2.653800000 | 0.734500000 | -2.648600000 |
| C | -3.058600000 | 2.067500000 | -2.559200000 |
| C | -2.507300000 | 2.880200000 | -1.569900000 |
| C | -1.572500000 | 2.349300000 | -0.688700000 |
| N | -1.185100000 | 1.062100000 | -0.775900000 |
| N | 0.267400000 | -1.052600000 | -1.373600000 |
| C | 1.032000000 | -0.370000000 | -2.463200000 |
| C | 0.916600000 | -2.343100000 | -0.990800000 |
| C | 1.890800000 | -2.106100000 | 0.129700000 |
| C | 2.282300000 | 0.213400000 | -1.866200000 |
| C | 3.561900000 | -0.095600000 | -2.308700000 |
| C | 4.652800000 | 0.440800000 | -1.621100000 |
| C | 4.419300000 | 1.275900000 | -0.531000000 |
| C | 3.103400000 | 1.538800000 | -0.154800000 |
| N | 2.051800000 | 1.013800000 | -0.803000000 |
| N | 1.589100000 | -1.081000000 | 0.968200000 |
| C | 2.391500000 | -0.833800000 | 2.022600000 |
| C | 3.525000000 | -1.591400000 | 2.277900000 |
| C | 3.851900000 | -2.632000000 | 1.409800000 |
| C | 3.018900000 | -2.892000000 | 0.323300000 |
| Fe | 0.163700000 | 0.166200000 | 0.344400000 |
| O | 0.211400000 | 1.129600000 | 1.704500000 |
| O | -1.436700000 | 2.042200000 | 2.347700000 |
| H | -1.660000000 | -1.434300000 | 2.723500000 |
| H | -3.498500000 | -3.153500000 | 2.672200000 |
| H | -4.264000000 | -4.030100000 | 0.448600000 |
| H | -3.179800000 | -3.150000000 | -1.644100000 |
| H | -3.056200000 | 0.066600000 | -3.409900000 |
| H | -3.793500000 | 2.466300000 | -3.259100000 |
| H | -2.797700000 | 3.926000000 | -1.472300000 |
| H | -1.116900000 | 2.921700000 | 0.116400000 |
| H | 1.241200000 | -1.072100000 | -3.283200000 |
| H | 0.408300000 | 0.446700000 | -2.853700000 |
| H | 0.141700000 | -3.037300000 | -0.633600000 |
| H | 1.404400000 | -2.808500000 | -1.859400000 |
| H | 3.702300000 | -0.762200000 | -3.159800000 |
| H | 5.670900000 | 0.199000000 | -1.928900000 |
| H | 5.243300000 | 1.710900000 | 0.034800000 |
| H | 2.865400000 | 2.173900000 | 0.699300000 |
| H | 2.098300000 | 0.015000000 | 2.639200000 |
| H | 4.150100000 | -1.350500000 | 3.137200000 |
| H | 4.749600000 | -3.229900000 | 1.570100000 |
| H | 3.244500000 | -3.691700000 | -0.381700000 |
| H | -2.050100000 | 1.328000000 | 2.038100000 |
| H | -1.379000000 | -1.695000000 | -2.642000000 |
| O | -3.846700000 | 0.521900000 | 1.928000000 |
| H | -4.198400000 | 1.410000000 | 0.044800000 |
| H | -4.289700000 | 1.270400000 | 2.387800000 |
| O | 1.002100000 | 3.653200000 | 1.126700000 |
| H | 0.885700000 | 4.353900000 | 3.116400000 |
| H | 0.687500000 | 2.735000000 | 1.372500000 |
| C | 0.468500000 | 4.551400000 | 2.113500000 |
| H | 0.744600000 | 5.573000000 | 1.816600000 |
| H | -0.628800000 | 4.475400000 | 2.173200000 |
| C | -4.387500000 | 0.474200000 | 0.591000000 |
| H | -3.878300000 | -0.343000000 | 0.071900000 |
| H | -5.469000000 | 0.266000000 | 0.599200000 |

### {[(N4Py)$Fe^{II}$(OOH)(**MeOH**)$_2$]$^+$}-**b** (S=2)

symmetry c1

| | | | |
|---|---|---|---|
| C | -2.360600000 | -2.310000000 | 0.619300000 |
| C | -3.158700000 | -3.246000000 | -0.024000000 |
| C | -3.213700000 | -3.240700000 | -1.418900000 |
| C | -2.474000000 | -2.291700000 | -2.125000000 |
| C | -1.698300000 | -1.391100000 | -1.408700000 |
| N | -1.636500000 | -1.402300000 | -0.058300000 |
| C | -0.927600000 | -0.247000000 | -2.043600000 |
| C | -1.712000000 | 1.012800000 | -1.715200000 |
| C | -2.543000000 | 1.650200000 | -2.624600000 |
| C | -3.260000000 | 2.770200000 | -2.200000000 |
| C | -3.108400000 | 3.215200000 | -0.885700000 |
| C | -2.258200000 | 2.524500000 | -0.032000000 |
| N | -1.581100000 | 1.431900000 | -0.433400000 |
| N | 0.407300000 | -0.170300000 | -1.382200000 |
| C | 1.163500000 | 1.060800000 | -1.714100000 |
| C | 1.197900000 | -1.412800000 | -1.645100000 |
| C | 2.627000000 | -1.285400000 | -1.178500000 |
| C | 1.968400000 | 1.487800000 | -0.509100000 |
| C | 3.253000000 | 2.005600000 | -0.611500000 |
| C | 3.944600000 | 2.340500000 | 0.552500000 |
| C | 3.319200000 | 2.161800000 | 1.785000000 |
| C | 2.025500000 | 1.652000000 | 1.809800000 |
| N | 1.362800000 | 1.311300000 | 0.688500000 |
| N | 2.857500000 | -1.282600000 | 0.151800000 |
| C | 4.102100000 | -1.082600000 | 0.605300000 |
| C | 5.185800000 | -0.884600000 | -0.245900000 |
| C | 4.963400000 | -0.916600000 | -1.619200000 |
| C | 3.667700000 | -1.122700000 | -2.091100000 |
| Fe | -0.420600000 | 0.141800000 | 0.768700000 |
| O | -1.244000000 | 0.508200000 | 2.435000000 |
| O | -0.466300000 | 0.128300000 | 3.681900000 |
| H | -2.294500000 | -2.253300000 | 1.705400000 |
| H | -3.731000000 | -3.965200000 | 0.561600000 |
| H | -3.827100000 | -3.967500000 | -1.952000000 |
| H | -2.495900000 | -2.249900000 | -3.214200000 |
| H | -2.624400000 | 1.278700000 | -3.646300000 |
| H | -3.922100000 | 3.293200000 | -2.890600000 |
| H | -3.646800000 | 4.089900000 | -0.521300000 |
| H | -2.119000000 | 2.811400000 | 1.009400000 |
| H | 1.803800000 | 0.932700000 | -2.601300000 |
| H | 0.448200000 | 1.862100000 | -1.947500000 |
| H | 0.705000000 | -2.241900000 | -1.116700000 |
| H | 1.191700000 | -1.641300000 | -2.724900000 |
| H | 3.718200000 | 2.111100000 | -1.591700000 |
| H | 4.966300000 | 2.717500000 | 0.496100000 |
| H | 3.826800000 | 2.400000000 | 2.719700000 |
| H | 1.490100000 | 1.481600000 | 2.745800000 |
| H | 4.220300000 | -1.068600000 | 1.689700000 |
| H | 6.179100000 | -0.709100000 | 0.165800000 |
| H | 5.786600000 | -0.769800000 | -2.319100000 |
| H | 3.460500000 | -1.136900000 | -3.161100000 |
| H | 0.195800000 | -0.493200000 | 3.264700000 |
| H | -0.852400000 | -0.382200000 | -3.136000000 |
| O | 0.869200000 | -1.382200000 | 1.805700000 |
| H | -0.228700000 | -2.872400000 | 2.728700000 |
| H | 1.713700000 | -1.337200000 | 1.179300000 |
| O | -3.890200000 | 0.904200000 | 2.117000000 |
| H | -4.625400000 | -0.857500000 | 1.208400000 |
| H | -2.940300000 | 0.619300000 | 2.266300000 |
| C | -4.427700000 | 0.205000000 | 0.984800000 |
| H | -5.378500000 | 0.687800000 | 0.719700000 |
| H | -3.762800000 | 0.251900000 | 0.108300000 |
| C | 0.557200000 | -2.784500000 | 1.969200000 |
| H | 0.195200000 | -3.220600000 | 1.026400000 |
| H | 1.450200000 | -3.330300000 | 2.307200000 |

### {[(N4Py)$Fe^{II}$(OOH)(**$H_2O$**)]$^+$}-**a** (S=0)

56

symmetry c1

| | | | |
|---|---|---|---|
| C | 0.927916000 | 2.621200000 | 1.369041000 |
| C | 1.577083000 | 3.839379000 | 1.204483000 |
| C | 2.221446000 | 4.118270000 | -0.002046000 |
| C | 2.189736000 | 3.169726000 | -1.028381000 |
| C | 1.527718000 | 1.976470000 | -0.797040000 |
| N | 0.919410000 | 1.700389000 | 0.388028000 |
| C | 1.362145000 | 0.838987000 | -1.778991000 |
| C | 2.185554000 | -0.314321000 | -1.254914000 |
| C | 3.341753000 | -0.813913000 | -1.827810000 |
| C | 3.965371000 | -1.904730000 | -1.216205000 |
| C | 3.396653000 | -2.455163000 | -0.067381000 |
| C | 2.231959000 | -1.904330000 | 0.456594000 |
| N | 1.645040000 | -0.839653000 | -0.121313000 |
| N | -0.084179000 | 0.390669000 | -1.630103000 |
| C | -0.401174000 | -0.880566000 | -2.348025000 |
| C | -1.080782000 | 1.471655000 | -1.890607000 |
| C | -2.109581000 | 1.498527000 | -0.792139000 |
| C | -1.161754000 | -1.808190000 | -1.437628000 |
| C | -1.938970000 | -2.858512000 | -1.910878000 |
| C | -2.562694000 | -3.707275000 | -0.997435000 |
| C | -2.397739000 | -3.471907000 | 0.367520000 |
| C | -1.617826000 | -2.398004000 | 0.773617000 |
| N | -1.003280000 | -1.588549000 | -0.109084000 |
| N | -1.714198000 | 0.972579000 | 0.394131000 |
| C | -2.541851000 | 1.029453000 | 1.454388000 |
| C | -3.799426000 | 1.613453000 | 1.371715000 |
| C | -4.220236000 | 2.142235000 | 0.152045000 |
| C | -3.361170000 | 2.081656000 | -0.944942000 |
| Fe | -0.007830000 | 0.027699000 | 0.345028000 |
| O | -0.071002000 | -0.323253000 | 2.098929000 |
| O | 1.441793000 | -0.156478000 | 2.909613000 |
| H | 0.392883000 | 2.344364000 | 2.274702000 |
| H | 1.570621000 | 4.564323000 | 2.018512000 |
| H | 2.728817000 | 5.071658000 | -0.151106000 |
| H | 2.655108000 | 3.358184000 | -1.995444000 |
| H | 3.734836000 | -0.368565000 | -2.741554000 |
| H | 4.875827000 | -2.327329000 | -1.642234000 |
| H | 3.849795000 | -3.314626000 | 0.427250000 |
| H | 1.745452000 | -2.289506000 | 1.351025000 |
| H | -0.954106000 | -0.685223000 | -3.277377000 |
| H | 0.543253000 | -1.371157000 | -2.627335000 |
| H | -0.555384000 | 2.438772000 | -1.885207000 |
| H | -1.544561000 | 1.356834000 | -2.879817000 |
| H | -2.051757000 | -3.005241000 | -2.985148000 |
| H | -3.177364000 | -4.536608000 | -1.348197000 |
| H | -2.874764000 | -4.106112000 | 1.114264000 |
| H | -1.457548000 | -2.133387000 | 1.818207000 |
| H | -2.141906000 | 0.590525000 | 2.369075000 |
| H | -4.438614000 | 1.643358000 | 2.253730000 |
| H | -5.207445000 | 2.594492000 | 0.052378000 |
| H | -3.653855000 | 2.486496000 | -1.913734000 |
| H | 1.100244000 | 0.483566000 | 3.575334000 |
| H | 1.625114000 | 1.119893000 | -2.809528000 |
| O | 1.002472000 | -2.690493000 | 3.612442000 |
| H | 0.159471000 | -2.819694000 | 3.131023000 |
| H | 1.191106000 | -1.711152000 | 3.419346000 |

### {[(N4Py)$Fe^{II}$(OOH)($\mathbf{H_2O}$)]$^{+}$}-**a** (S=1)

56

symmetry c1

| | | | |
|---|---|---|---|
| C | 0.842163000 | 2.464329000 | 1.585168000 |
| C | 1.604841000 | 3.624299000 | 1.515805000 |
| C | 2.306916000 | 3.911864000 | 0.347300000 |
| C | 2.236039000 | 3.031820000 | -0.735150000 |
| C | 1.466891000 | 1.890598000 | -0.599463000 |
| N | 0.790378000 | 1.618469000 | 0.544623000 |
| C | 1.306814000 | 0.794024000 | -1.630177000 |
| C | 2.071781000 | -0.397886000 | -1.104306000 |
| C | 3.240868000 | -0.906408000 | -1.636194000 |
| C | 3.824286000 | -2.009380000 | -1.005511000 |
| C | 3.223328000 | -2.544412000 | 0.128596000 |
| C | 2.042705000 | -1.988334000 | 0.617711000 |
| N | 1.488812000 | -0.935181000 | -0.001201000 |
| N | -0.147943000 | 0.390118000 | -1.571824000 |
| C | -0.469617000 | -0.848075000 | -2.346347000 |
| C | -1.095107000 | 1.509722000 | -1.853610000 |
| C | -2.220400000 | 1.473888000 | -0.862521000 |
| C | -1.292597000 | -1.785008000 | -1.508427000 |
| C | -2.066364000 | -2.800850000 | -2.054429000 |
| C | -2.727369000 | -3.682167000 | -1.202705000 |
| C | -2.600532000 | -3.522416000 | 0.176010000 |
| C | -1.828335000 | -2.479216000 | 0.665063000 |
| N | -1.190508000 | -1.634152000 | -0.166450000 |
| N | -1.932051000 | 0.902813000 | 0.331459000 |
| C | -2.852756000 | 0.886032000 | 1.313299000 |
| C | -4.106548000 | 1.453114000 | 1.136082000 |
| C | -4.420848000 | 2.032393000 | -0.091552000 |
| C | -3.464417000 | 2.040583000 | -1.104339000 |
| Fe | -0.220491000 | -0.054282000 | 0.435929000 |
| O | -0.308189000 | -0.357989000 | 2.064510000 |
| O | 2.904515000 | 0.013246000 | 2.863995000 |
| H | 0.273121000 | 2.170853000 | 2.465181000 |
| H | 1.641293000 | 4.291344000 | 2.375918000 |
| H | 2.905198000 | 4.819834000 | 0.273778000 |
| H | 2.762678000 | 3.227312000 | -1.668193000 |
| H | 3.676468000 | -0.456840000 | -2.527587000 |
| H | 4.743149000 | -2.441564000 | -1.402792000 |
| H | 3.663556000 | -3.393208000 | 0.651808000 |
| H | 1.581356000 | -2.308552000 | 1.564448000 |
| H | -0.976247000 | -0.596108000 | -3.286695000 |
| H | 0.470750000 | -1.356785000 | -2.606061000 |
| H | -0.559854000 | 2.463182000 | -1.729286000 |
| H | -1.455984000 | 1.467144000 | -2.889389000 |
| H | -2.141585000 | -2.895677000 | -3.137227000 |
| H | -3.339964000 | -4.484883000 | -1.613206000 |
| H | -3.101852000 | -4.190291000 | 0.875082000 |
| H | -1.697263000 | -2.282761000 | 1.727377000 |
| H | -2.547077000 | 0.399197000 | 2.237311000 |
| H | -4.826606000 | 1.424871000 | 1.953100000 |
| H | -5.404564000 | 2.469589000 | -0.262688000 |
| H | -3.673139000 | 2.482961000 | -2.077779000 |
| H | 2.426976000 | 0.358086000 | 2.086151000 |
| H | 1.625965000 | 1.100737000 | -2.635724000 |
| O | 1.470527000 | -1.942920000 | 3.549725000 |
| H | 0.618667000 | -1.539341000 | 3.276174000 |
| H | 2.277865000 | -0.845665000 | 3.161256000 |

### {[(N4Py)$Fe^{II}$(OOH)($\mathbf{H_2O}$)]$^{+}$}-**a** (S=2)

56

symmetry c1

| | | | |
|---|---|---|---|
| C | 1.235046000 | 2.975201000 | 1.266493000 |
| C | 1.818407000 | 4.170669000 | 0.858408000 |
| C | 2.304496000 | 4.274330000 | -0.445306000 |
| C | 2.195754000 | 3.177664000 | -1.301440000 |
| C | 1.601894000 | 2.020392000 | -0.817038000 |
| N | 1.133953000 | 1.918405000 | 0.445432000 |
| C | 1.442531000 | 0.745976000 | -1.631276000 |
| C | 2.440349000 | -0.241812000 | -1.059355000 |
| C | 3.625337000 | -0.560441000 | -1.707454000 |
| C | 4.523359000 | -1.422571000 | -1.078919000 |
| C | 4.197632000 | -1.927692000 | 0.178889000 |
| C | 2.987387000 | -1.565853000 | 0.760919000 |
| N | 2.110609000 | -0.738621000 | 0.159115000 |
| N | 0.074171000 | 0.227777000 | -1.380371000 |
| C | -0.182354000 | -1.128350000 | -1.917958000 |
| C | -1.002065000 | 1.178420000 | -1.742042000 |
| C | -2.117419000 | 1.155578000 | -0.722742000 |
| C | -1.280584000 | -1.749821000 | -1.095204000 |
| C | -2.437631000 | -2.279203000 | -1.649191000 |
| C | -3.447033000 | -2.738970000 | -0.801419000 |
| C | -3.258190000 | -2.656018000 | 0.576491000 |
| C | -2.068702000 | -2.123445000 | 1.058141000 |
| N | -1.090552000 | -1.675447000 | 0.245446000 |
| N | -1.740155000 | 1.004315000 | 0.565115000 |
| C | -2.683884000 | 1.033161000 | 1.525091000 |
| C | -4.034559000 | 1.196389000 | 1.241449000 |
| C | -4.427019000 | 1.326580000 | -0.089739000 |
| C | -3.450913000 | 1.310224000 | -1.085559000 |
| Fe | 0.193101000 | 0.009152000 | 0.897249000 |
| O | -0.059628000 | -0.001168000 | 2.779401000 |
| O | 0.848407000 | -1.024211000 | 3.421709000 |
| H | 0.828710000 | 2.837900000 | 2.269028000 |
| H | 1.882300000 | 5.008441000 | 1.552581000 |
| H | 2.755540000 | 5.203065000 | -0.796190000 |
| H | 2.552529000 | 3.222469000 | -2.330520000 |
| H | 3.835442000 | -0.142156000 | -2.691820000 |
| H | 5.459268000 | -1.697662000 | -1.565767000 |
| H | 4.868413000 | -2.603538000 | 0.709198000 |
| H | 2.690795000 | -1.960513000 | 1.729256000 |
| H | -0.442370000 | -1.102798000 | -2.988604000 |
| H | 0.731444000 | -1.729350000 | -1.802716000 |
| H | -0.582582000 | 2.196241000 | -1.748923000 |
| H | -1.389302000 | 0.978221000 | -2.753551000 |
| H | -2.557270000 | -2.305466000 | -2.732428000 |
| H | -4.374123000 | -3.139215000 | -1.213437000 |
| H | -4.026550000 | -2.986151000 | 1.275624000 |
| H | -1.876152000 | -2.014116000 | 2.125777000 |
| H | -2.323048000 | 0.886755000 | 2.543590000 |
| H | -4.763518000 | 1.202579000 | 2.052012000 |
| H | -5.480353000 | 1.432061000 | -0.351935000 |
| H | -3.720368000 | 1.403515000 | -2.138015000 |
| H | 1.703376000 | -0.528735000 | 3.425557000 |
| H | 1.645704000 | 0.932417000 | -2.699076000 |
| O | 0.676403000 | -3.408110000 | 1.976378000 |
| H | 0.329754000 | -3.042036000 | 1.131890000 |
| H | 0.736472000 | -2.589298000 | 2.540027000 |

{[(N4Py)$Fe^{II}$(OOH)(**$H_2O$**)]$^+$}-**b** (S=0)

56

symmetry c1

| | | | |
|---|---|---|---|
| C | 2.570016000 | -1.455880000 | -0.406767000 |
| C | 3.798892000 | -1.792168000 | 0.149532000 |
| C | 4.260113000 | -1.103131000 | 1.271791000 |
| C | 3.454674000 | -0.106395000 | 1.827141000 |
| C | 2.237365000 | 0.168317000 | 1.227179000 |
| N | 1.801659000 | -0.473779000 | 0.105855000 |
| C | 1.217977000 | 1.158136000 | 1.731866000 |
| C | 1.169250000 | 2.293363000 | 0.735025000 |
| C | 1.584130000 | 3.596072000 | 0.951663000 |
| C | 1.430894000 | 4.521904000 | -0.085103000 |
| C | 0.862081000 | 4.106350000 | -1.291620000 |
| C | 0.467379000 | 2.782477000 | -1.443935000 |
| N | 0.630126000 | 1.888177000 | -0.448847000 |
| N | -0.116538000 | 0.438313000 | 1.582576000 |
| C | -1.298392000 | 1.309598000 | 1.850472000 |
| C | -0.193053000 | -0.877146000 | 2.293289000 |
| C | -0.771069000 | -1.927205000 | 1.377087000 |
| C | -2.327225000 | 1.120679000 | 0.768753000 |
| C | -3.672506000 | 1.419956000 | 0.940877000 |
| C | -4.542022000 | 1.292574000 | -0.143091000 |
| C | -4.032562000 | 0.867427000 | -1.369494000 |
| C | -2.679817000 | 0.567669000 | -1.471344000 |
| N | -1.840962000 | 0.689483000 | -0.423853000 |
| N | -0.678012000 | -1.662336000 | 0.049698000 |
| C | -1.158417000 | -2.552469000 | -0.842185000 |
| C | -1.711347000 | -3.762160000 | -0.445340000 |
| C | -1.795524000 | -4.051600000 | 0.918027000 |
| C | -1.328152000 | -3.114227000 | 1.839563000 |
| Fe | 0.006213000 | 0.089157000 | -0.376671000 |
| O | -0.071734000 | -0.186840000 | -2.236260000 |
| O | 1.273517000 | -0.339291000 | -2.950169000 |
| H | 2.171628000 | -1.962713000 | -1.279340000 |
| H | 4.387057000 | -2.591741000 | -0.301051000 |
| H | 5.221614000 | -1.351164000 | 1.721582000 |
| H | 3.752357000 | 0.434601000 | 2.724842000 |
| H | 1.998924000 | 3.884545000 | 1.917323000 |
| H | 1.736062000 | 5.559005000 | 0.055392000 |
| H | 0.720678000 | 4.806582000 | -2.115010000 |
| H | 0.016098000 | 2.381057000 | -2.351258000 |
| H | -1.716364000 | 1.123011000 | 2.849163000 |
| H | -0.968981000 | 2.359685000 | 1.822841000 |
| H | 0.826147000 | -1.185491000 | 2.570890000 |
| H | -0.773208000 | -0.794551000 | 3.222943000 |
| H | -4.031920000 | 1.749291000 | 1.916061000 |
| H | -5.602545000 | 1.518543000 | -0.028150000 |
| H | -4.675898000 | 0.755074000 | -2.242193000 |
| H | -2.213282000 | 0.218189000 | -2.392849000 |
| H | -1.076483000 | -2.228415000 | -1.880897000 |
| H | -2.081400000 | -4.460341000 | -1.196442000 |
| H | -2.233137000 | -4.989413000 | 1.261332000 |
| H | -1.394387000 | -3.298679000 | 2.912031000 |
| H | 1.281390000 | -1.330076000 | -3.019807000 |
| H | 1.422205000 | 1.496749000 | 2.758559000 |
| O | 1.120678000 | -3.203083000 | -3.063743000 |
| H | 1.955863000 | -3.692862000 | -3.242216000 |
| H | 0.760746000 | -3.634707000 | -2.257327000 |

{[(N4Py)$Fe^{II}$(OOH)(**$H_2O$**)]$^+$}-**b** (S=1)

56

symmetry c1

| | | | |
|---|---|---|---|
| C | 2.663100000 | -1.700100000 | -0.458100000 |
| C | 3.873300000 | -1.974400000 | 0.184000000 |
| C | 4.222000000 | -1.252600000 | 1.321000000 |
| C | 3.356400000 | -0.266300000 | 1.804000000 |
| C | 2.168200000 | -0.051800000 | 1.123100000 |
| N | 1.848800000 | -0.756800000 | 0.018000000 |
| C | 1.135700000 | 0.984400000 | 1.527700000 |
| C | 1.118400000 | 2.045400000 | 0.440500000 |
| C | 1.735000000 | 3.279200000 | 0.559600000 |
| C | 1.717800000 | 4.147100000 | -0.533100000 |
| C | 1.079000000 | 3.757800000 | -1.706900000 |
| C | 0.467900000 | 2.511400000 | -1.764800000 |
| N | 0.497100000 | 1.673600000 | -0.713300000 |
| N | -0.211300000 | 0.322500000 | 1.516200000 |
| C | -1.339200000 | 1.251900000 | 1.783100000 |
| C | -0.309500000 | -0.966000000 | 2.251100000 |
| C | -0.991900000 | -2.030700000 | 1.427200000 |
| C | -2.482300000 | 0.998100000 | 0.838400000 |
| C | -3.794800000 | 1.314100000 | 1.165400000 |
| C | -4.800500000 | 1.140300000 | 0.219200000 |
| C | -4.469100000 | 0.645900000 | -1.039600000 |
| C | -3.147700000 | 0.326800000 | -1.312100000 |
| N | -2.175800000 | 0.506000000 | -0.392600000 |
| N | -0.985700000 | -1.898900000 | 0.073100000 |
| C | -1.524000000 | -2.863000000 | -0.703400000 |
| C | -2.077200000 | -4.010700000 | -0.158400000 |
| C | -2.094400000 | -4.158400000 | 1.226900000 |
| C | -1.549700000 | -3.155800000 | 2.023400000 |
| Fe | -0.333000000 | -0.171600000 | -0.686000000 |
| O | -0.427700000 | -0.492600000 | -2.291800000 |
| O | 2.820700000 | 0.265300000 | -2.935400000 |
| H | 2.346800000 | -2.162700000 | -1.414800000 |
| H | 4.531100000 | -2.744500000 | -0.219100000 |
| H | 5.161400000 | -1.452600000 | 1.837800000 |
| H | 3.592100000 | 0.312600000 | 2.696200000 |
| H | 2.219000000 | 3.553400000 | 1.496000000 |
| H | 2.198300000 | 5.123000000 | -0.462500000 |
| H | 1.047600000 | 4.409000000 | -2.579100000 |
| H | -0.043200000 | 2.141000000 | -2.651400000 |
| H | -1.665500000 | 1.183900000 | 2.830400000 |
| H | -0.990400000 | 2.281900000 | 1.613300000 |
| H | 0.706000000 | -1.321600000 | 2.480600000 |
| H | -0.828600000 | -0.826700000 | 3.209700000 |
| H | -4.016400000 | 1.694800000 | 2.161900000 |
| H | -5.834600000 | 1.381100000 | 0.466400000 |
| H | -5.223200000 | 0.490500000 | -1.809700000 |
| H | -2.825900000 | -0.086700000 | -2.265300000 |
| H | -1.494400000 | -2.674200000 | -1.774700000 |
| H | -2.497300000 | -4.767900000 | -0.819000000 |
| H | -2.534800000 | -5.044400000 | 1.684900000 |
| H | -1.549700000 | -3.237600000 | 3.110100000 |
| H | 2.376200000 | 0.583500000 | -2.126200000 |
| H | 1.371800000 | 1.421900000 | 2.509000000 |
| O | 1.781400000 | -2.028200000 | -3.248900000 |
| H | 0.881600000 | -1.758200000 | -2.963200000 |
| H | 2.373600000 | -0.717600000 | -3.074200000 |

## {[(N4Py)$Fe^{II}$(OOH)($\mathbf{H_2O}$)]$^+$}-**b** (S=2)

56

symmetry c1

| | | | |
|---|---|---|---|
| C | 3.301220000 | -1.049622000 | -0.795290000 |
| C | 4.548280000 | -1.176940000 | -0.196137000 |
| C | 4.752648000 | -0.630642000 | 1.071040000 |
| C | 3.699918000 | 0.047357000 | 1.684278000 |
| C | 2.482887000 | 0.130249000 | 1.022080000 |
| N | 2.266743000 | -0.424701000 | -0.196894000 |
| C | 1.311858000 | 0.923524000 | 1.561990000 |
| C | 1.233090000 | 2.191600000 | 0.726341000 |
| C | 1.560584000 | 3.448918000 | 1.214616000 |
| C | 1.457562000 | 4.544894000 | 0.356932000 |
| C | 1.030357000 | 4.339472000 | -0.955345000 |
| C | 0.720319000 | 3.048736000 | -1.370141000 |
| N | 0.822912000 | 1.989459000 | -0.548111000 |
| N | 0.068287000 | 0.145885000 | 1.311954000 |
| C | -1.170045000 | 0.889943000 | 1.647070000 |
| C | 0.075527000 | -1.210642000 | 1.920725000 |
| C | -0.978341000 | -2.009165000 | 1.203881000 |
| C | -2.248673000 | 0.667552000 | 0.611307000 |
| C | -3.598588000 | 0.648398000 | 0.947048000 |
| C | -4.546180000 | 0.496058000 | -0.063614000 |
| C | -4.112414000 | 0.379490000 | -1.383307000 |
| C | -2.746701000 | 0.394394000 | -1.638443000 |
| N | -1.828029000 | 0.526578000 | -0.662787000 |
| N | -0.791526000 | -2.098850000 | -0.134268000 |
| C | -1.778343000 | -2.624507000 | -0.883400000 |
| C | -2.977923000 | -3.071966000 | -0.340283000 |
| C | -3.154902000 | -3.003519000 | 1.039195000 |
| C | -2.133822000 | -2.464917000 | 1.824452000 |
| Fe | 0.277313000 | -0.044399000 | -0.958013000 |
| O | -0.118221000 | -0.545933000 | -2.744234000 |
| O | 1.223888000 | -1.028145000 | -3.219165000 |
| H | 3.106418000 | -1.438947000 | -1.791090000 |
| H | 5.346013000 | -1.700722000 | -0.722910000 |
| H | 5.716665000 | -0.725291000 | 1.571521000 |
| H | 3.815904000 | 0.505012000 | 2.666807000 |
| H | 1.875635000 | 3.567924000 | 2.251248000 |
| H | 1.696623000 | 5.547376000 | 0.712687000 |
| H | 0.931451000 | 5.170597000 | -1.653455000 |
| H | 0.372785000 | 2.836416000 | -2.381410000 |
| H | -1.531569000 | 0.631878000 | 2.655115000 |
| H | -0.940790000 | 1.966534000 | 1.651685000 |
| H | 1.062430000 | -1.662984000 | 1.749599000 |
| H | -0.108621000 | -1.167804000 | 3.006372000 |
| H | -3.899896000 | 0.735903000 | 1.991417000 |
| H | -5.609227000 | 0.461403000 | 0.178048000 |
| H | -4.818036000 | 0.258242000 | -2.205162000 |
| H | -2.349656000 | 0.270443000 | -2.646510000 |
| H | -1.591924000 | -2.639604000 | -1.958183000 |
| H | -3.759731000 | -3.457972000 | -0.994058000 |
| H | -4.085566000 | -3.338724000 | 1.498075000 |
| H | -2.250074000 | -2.357242000 | 2.902966000 |
| H | 1.227187000 | -1.948744000 | -2.808631000 |
| H | 1.458784000 | 1.165983000 | 2.627462000 |
| O | 1.176390000 | -3.328382000 | -1.715102000 |
| H | 2.049307000 | -3.522619000 | -1.307823000 |
| H | 0.662990000 | -2.859327000 | -0.997774000 |

## {[(N4Py)$Fe^{II}$(OOH)($\mathbf{H_2O}$)$_2$]$^+$} (S=0)

59

symmetry c1

| | | | |
|---|---|---|---|
| C | 2.007714000 | -2.293676000 | -0.517824000 |
| C | 3.052973000 | -3.069115000 | -0.027549000 |
| C | 3.602642000 | -2.774325000 | 1.219435000 |
| C | 3.085076000 | -1.705728000 | 1.955340000 |
| C | 2.044007000 | -0.976668000 | 1.408037000 |
| N | 1.514253000 | -1.261038000 | 0.187554000 |
| C | 1.359693000 | 0.206431000 | 2.054158000 |
| C | 1.763268000 | 1.428924000 | 1.262850000 |
| C | 2.569516000 | 2.462004000 | 1.706676000 |
| C | 2.814981000 | 3.527148000 | 0.836286000 |
| C | 2.235584000 | 3.518466000 | -0.432431000 |
| C | 1.435146000 | 2.447583000 | -0.815366000 |
| N | 1.216362000 | 1.416980000 | 0.019252000 |
| N | -0.118886000 | 0.015949000 | 1.767035000 |
| C | -0.966869000 | 1.189584000 | 2.139758000 |
| C | -0.659049000 | -1.281054000 | 2.275196000 |
| C | -1.557600000 | -1.883225000 | 1.233372000 |
| C | -1.877409000 | 1.555177000 | 0.997469000 |
| C | -3.025434000 | 2.320166000 | 1.165859000 |
| C | -3.777851000 | 2.672443000 | 0.046647000 |
| C | -3.361560000 | 2.242613000 | -1.213076000 |
| C | -2.213157000 | 1.469864000 | -1.311555000 |
| N | -1.483622000 | 1.137258000 | -0.230517000 |
| N | -1.291415000 | -1.519482000 | -0.047876000 |
| C | -2.077056000 | -1.993374000 | -1.035567000 |
| C | -3.110069000 | -2.888841000 | -0.795235000 |
| C | -3.364734000 | -3.291079000 | 0.514522000 |
| C | -2.583654000 | -2.766716000 | 1.542820000 |
| Fe | 0.035860000 | -0.085382000 | -0.232459000 |
| O | 0.090997000 | -0.176529000 | -2.007477000 |
| O | 1.740681000 | 0.064977000 | -2.684145000 |
| H | 1.545861000 | -2.478407000 | -1.484368000 |
| H | 3.426559000 | -3.900188000 | -0.625288000 |
| H | 4.417571000 | -3.375643000 | 1.622550000 |
| H | 3.471234000 | -1.450132000 | 2.941436000 |
| H | 2.981410000 | 2.439982000 | 2.715199000 |
| H | 3.438878000 | 4.362548000 | 1.154248000 |
| H | 2.396892000 | 4.340363000 | -1.129567000 |
| H | 0.954411000 | 2.383471000 | -1.790280000 |
| H | -1.534726000 | 0.988982000 | 3.058424000 |
| H | -0.309454000 | 2.048462000 | 2.342301000 |
| H | 0.183484000 | -1.966573000 | 2.448717000 |
| H | -1.178598000 | -1.149372000 | 3.234298000 |
| H | -3.322720000 | 2.633545000 | 2.166788000 |
| H | -4.683323000 | 3.269607000 | 0.158296000 |
| H | -3.922792000 | 2.492591000 | -2.112937000 |
| H | -1.834734000 | 1.079500000 | -2.254669000 |
| H | -1.862425000 | -1.621355000 | -2.034109000 |
| H | -3.711293000 | -3.250306000 | -1.628894000 |
| H | -4.174144000 | -3.986853000 | 0.736475000 |
| H | -2.769219000 | -3.031639000 | 2.583645000 |
| H | 1.773399000 | -0.803778000 | -3.151400000 |
| H | 1.580592000 | 0.296087000 | 3.127260000 |
| O | 0.138437000 | -2.357888000 | -3.447686000 |
| H | -0.170271000 | -3.132255000 | -2.932341000 |
| H | 0.034596000 | -1.560336000 | -2.813274000 |
| O | 0.535501000 | 1.897313000 | -4.181236000 |
| H | -0.388840000 | 1.759146000 | -3.888963000 |
| H | 1.023606000 | 1.171491000 | -3.661681000 |

### {[(N4Py)$Fe^{II}$(OOH)($\mathbf{H_2O}$)$_2$]$^+$} (S=1)

59
symmetry c1

| | | | |
|---|---|---|---|
| C | 1.800339000 | -2.330969000 | -0.623674000 |
| C | 2.880097000 | -3.089024000 | -0.183198000 |
| C | 3.498320000 | -2.767074000 | 1.022426000 |
| C | 3.022724000 | -1.689794000 | 1.772789000 |
| C | 1.951593000 | -0.970251000 | 1.276679000 |
| N | 1.362331000 | -1.284283000 | 0.094829000 |
| C | 1.310501000 | 0.230198000 | 1.934154000 |
| C | 1.678776000 | 1.432782000 | 1.097823000 |
| C | 2.528571000 | 2.455151000 | 1.473553000 |
| C | 2.761282000 | 3.484964000 | 0.557093000 |
| C | 2.145856000 | 3.444167000 | -0.689883000 |
| C | 1.296534000 | 2.386709000 | -1.012852000 |
| N | 1.080629000 | 1.409913000 | -0.120408000 |
| N | -0.178774000 | 0.045440000 | 1.737029000 |
| C | -0.992155000 | 1.238406000 | 2.130473000 |
| C | -0.707203000 | -1.232088000 | 2.308018000 |
| C | -1.634472000 | -1.880168000 | 1.322004000 |
| C | -1.967282000 | 1.583489000 | 1.042222000 |
| C | -3.082901000 | 2.382323000 | 1.257532000 |
| C | -3.886445000 | 2.730421000 | 0.175167000 |
| C | -3.554904000 | 2.267355000 | -1.097088000 |
| C | -2.438358000 | 1.459330000 | -1.250158000 |
| N | -1.665721000 | 1.130817000 | -0.197477000 |
| N | -1.438570000 | -1.550640000 | 0.023347000 |
| C | -2.190360000 | -2.116450000 | -0.939342000 |
| C | -3.161351000 | -3.059149000 | -0.637389000 |
| C | -3.372465000 | -3.408954000 | 0.695118000 |
| C | -2.602239000 | -2.807211000 | 1.686715000 |
| Fe | -0.160063000 | -0.102709000 | -0.307822000 |
| O | -0.157333000 | -0.258195000 | -1.965993000 |
| O | 2.761002000 | 0.313780000 | -2.848747000 |
| H | 1.279255000 | -2.535043000 | -1.559150000 |
| H | 3.223701000 | -3.926584000 | -0.788820000 |
| H | 4.340431000 | -3.355908000 | 1.385360000 |
| H | 3.465643000 | -1.417872000 | 2.729618000 |
| H | 2.986949000 | 2.450692000 | 2.461472000 |
| H | 3.418906000 | 4.312666000 | 0.823767000 |
| H | 2.318663000 | 4.227225000 | -1.427849000 |
| H | 0.844612000 | 2.259046000 | -2.015110000 |
| H | -1.499947000 | 1.060736000 | 3.086845000 |
| H | -0.316916000 | 2.095883000 | 2.270456000 |
| H | 0.137062000 | -1.913970000 | 2.488135000 |
| H | -1.197033000 | -1.054078000 | 3.273891000 |
| H | -3.309458000 | 2.727261000 | 2.265860000 |
| H | -4.766293000 | 3.356577000 | 0.324783000 |
| H | -4.156949000 | 2.518559000 | -1.969310000 |
| H | -2.126767000 | 1.051533000 | -2.209155000 |
| H | -1.992592000 | -1.776159000 | -1.952825000 |
| H | -3.749318000 | -3.499544000 | -1.441524000 |
| H | -4.137729000 | -4.138207000 | 0.961578000 |
| H | -2.744543000 | -3.049486000 | 2.739292000 |
| H | 2.355553000 | -0.054274000 | -2.040250000 |
| H | 1.587138000 | 0.339008000 | 2.991499000 |
| O | 0.476450000 | -2.225756000 | -3.700871000 |
| H | -0.370235000 | -2.719369000 | -3.749815000 |
| H | 0.260066000 | -1.441323000 | -3.121965000 |
| O | 0.801484000 | 1.606193000 | -3.840466000 |
| H | 0.102808000 | 0.959154000 | -3.613918000 |
| H | 1.930283000 | 0.849299000 | -3.281131000 |

### {[(N4Py)$Fe^{II}$(OOH)($\mathbf{H_2O}$)$_2$]$^+$} (S=2)

59
symmetry c1

| | | | |
|---|---|---|---|
| C | 2.397414000 | -2.354244000 | -0.447511000 |
| C | 3.435279000 | -3.047926000 | 0.166362000 |
| C | 3.820909000 | -2.688803000 | 1.457801000 |
| C | 3.157835000 | -1.637736000 | 2.092972000 |
| C | 2.138309000 | -0.988592000 | 1.412030000 |
| N | 1.757160000 | -1.338605000 | 0.160686000 |
| C | 1.389323000 | 0.204969000 | 1.978524000 |
| C | 1.905474000 | 1.421020000 | 1.230458000 |
| C | 2.763110000 | 2.353111000 | 1.796684000 |
| C | 3.202308000 | 3.417756000 | 1.008908000 |
| C | 2.762475000 | 3.507878000 | -0.311290000 |
| C | 1.899180000 | 2.534178000 | -0.805744000 |
| N | 1.479360000 | 1.503115000 | -0.051230000 |
| N | -0.050493000 | 0.045696000 | 1.644894000 |
| C | -0.883392000 | 1.230567000 | 1.950332000 |
| C | -0.656489000 | -1.204132000 | 2.150883000 |
| C | -1.725508000 | -1.672588000 | 1.195609000 |
| C | -1.958299000 | 1.402076000 | 0.903662000 |
| C | -3.252309000 | 1.800100000 | 1.220746000 |
| C | -4.181998000 | 1.966742000 | 0.194277000 |
| C | -3.784772000 | 1.726429000 | -1.120618000 |
| C | -2.478186000 | 1.316193000 | -1.355847000 |
| N | -1.576903000 | 1.157951000 | -0.369740000 |
| N | -1.395569000 | -1.606913000 | -0.114549000 |
| C | -2.297807000 | -2.016964000 | -1.023674000 |
| C | -3.554896000 | -2.497811000 | -0.673868000 |
| C | -3.897575000 | -2.552973000 | 0.676002000 |
| C | -2.963474000 | -2.135737000 | 1.624821000 |
| Fe | 0.174523000 | -0.128009000 | -0.627000000 |
| O | -0.101844000 | -0.396111000 | -2.523697000 |
| O | 1.314145000 | -0.044121000 | -2.917860000 |
| H | 2.050316000 | -2.612236000 | -1.447741000 |
| H | 3.925383000 | -3.864967000 | -0.363476000 |
| H | 4.622109000 | -3.224152000 | 1.968247000 |
| H | 3.422511000 | -1.328153000 | 3.104249000 |
| H | 3.072286000 | 2.250610000 | 2.836877000 |
| H | 3.870414000 | 4.172262000 | 1.425352000 |
| H | 3.075859000 | 4.329179000 | -0.955970000 |
| H | 1.515225000 | 2.572595000 | -1.825970000 |
| H | -1.318875000 | 1.166733000 | 2.960325000 |
| H | -0.244567000 | 2.125680000 | 1.921864000 |
| H | 0.123934000 | -1.979058000 | 2.191785000 |
| H | -1.056016000 | -1.076796000 | 3.169644000 |
| H | -3.529532000 | 1.966392000 | 2.262160000 |
| H | -5.205719000 | 2.267393000 | 0.420156000 |
| H | -4.479374000 | 1.834532000 | -1.953511000 |
| H | -2.130171000 | 1.059998000 | -2.357402000 |
| H | -1.999563000 | -1.926949000 | -2.068137000 |
| H | -4.253802000 | -2.806427000 | -1.451449000 |
| H | -4.881997000 | -2.904369000 | 0.987328000 |
| H | -3.196831000 | -2.154269000 | 2.689852000 |
| H | 1.600970000 | -0.925701000 | -3.289000000 |
| H | 1.571609000 | 0.304068000 | 3.061412000 |
| O | 0.688073000 | -2.791748000 | -3.296468000 |
| H | 0.415566000 | -2.915268000 | -4.232490000 |
| H | 0.131150000 | -2.017987000 | -2.977953000 |
| O | 0.283114000 | 2.337205000 | -3.856038000 |
| H | -0.540553000 | 2.270518000 | -3.328937000 |
| H | 0.713034000 | 1.456344000 | -3.689084000 |

## {[(N4Py)$Fe^{II}$(OOH)(**HOOH**)]$^{+}$}  (S=0)

57
symmetry c1

| | | | |
|---|---|---|---|
| H | 1.272009000 | 1.548731000 | -2.569001000 |
| O | 1.860702000 | 2.309976000 | -2.997903000 |
| O | 1.527846000 | 3.471836000 | -2.125874000 |
| H | 2.154687000 | 3.332353000 | -1.372017000 |
| C | -1.234180000 | 2.616031000 | -0.711615000 |
| C | -2.218484000 | 3.560094000 | -0.443104000 |
| C | -3.230164000 | 3.262669000 | 0.470429000 |
| C | -3.221729000 | 2.020622000 | 1.110316000 |
| C | -2.217979000 | 1.123106000 | 0.791892000 |
| N | -1.245731000 | 1.402378000 | -0.122809000 |
| C | -2.038396000 | -0.251831000 | 1.390077000 |
| C | -2.388693000 | -1.244855000 | 0.310054000 |
| C | -3.485978000 | -2.088931000 | 0.300796000 |
| C | -3.630712000 | -2.971334000 | -0.772083000 |
| C | -2.665446000 | -2.979492000 | -1.780176000 |
| C | -1.590840000 | -2.100492000 | -1.709369000 |
| N | -1.464194000 | -1.232738000 | -0.687392000 |
| N | -0.535801000 | -0.401473000 | 1.587542000 |
| C | -0.106645000 | -1.782792000 | 1.974357000 |
| C | 0.054006000 | 0.655923000 | 2.466744000 |
| C | 1.356867000 | 1.113180000 | 1.875645000 |
| C | 1.034691000 | -2.236849000 | 1.101183000 |
| C | 1.879422000 | -3.283276000 | 1.449379000 |
| C | 2.861837000 | -3.691760000 | 0.547153000 |
| C | 2.967582000 | -3.039879000 | -0.681150000 |
| C | 2.100985000 | -1.992222000 | -0.964509000 |
| N | 1.155413000 | -1.597984000 | -0.090176000 |
| N | 1.468001000 | 0.953828000 | 0.530561000 |
| C | 2.630850000 | 1.294743000 | -0.065607000 |
| C | 3.682978000 | 1.871619000 | 0.633202000 |
| C | 3.553200000 | 2.075896000 | 2.006658000 |
| C | 2.377463000 | 1.670765000 | 2.635896000 |
| Fe | 0.010009000 | -0.053742000 | -0.280364000 |
| O | 0.575818000 | 0.323865000 | -2.048832000 |
| O | -0.564267000 | 0.318052000 | -3.104011000 |
| H | -0.400792000 | 2.810850000 | -1.386860000 |
| H | -2.181537000 | 4.526009000 | -0.947047000 |
| H | -4.005853000 | 3.993946000 | 0.697980000 |
| H | -3.972037000 | 1.757881000 | 1.855628000 |
| H | -4.198656000 | -2.067556000 | 1.124839000 |
| H | -4.478883000 | -3.655172000 | -0.811113000 |
| H | -2.739759000 | -3.665774000 | -2.623547000 |
| H | -0.811030000 | -2.051115000 | -2.465324000 |
| H | 0.156845000 | -1.829684000 | 3.039587000 |
| H | -0.952005000 | -2.469240000 | 1.815515000 |
| H | -0.637918000 | 1.510491000 | 2.496686000 |
| H | 0.177807000 | 0.295889000 | 3.497261000 |
| H | 1.765796000 | -3.770688000 | 2.417807000 |
| H | 3.537589000 | -4.508403000 | 0.802996000 |
| H | 3.720207000 | -3.330099000 | -1.414186000 |
| H | 2.125754000 | -1.429313000 | -1.897215000 |
| H | 2.701469000 | 1.082079000 | -1.130771000 |
| H | 4.595828000 | 2.139975000 | 0.102064000 |
| H | 4.364102000 | 2.520318000 | 2.583993000 |
| H | 2.248999000 | 1.776941000 | 3.713055000 |
| H | -1.243088000 | 0.852449000 | -2.626962000 |
| H | -2.615114000 | -0.397501000 | 2.315373000 |

## {[(N4Py)$Fe^{II}$(OOH)(**HOOH**)]$^{+}$}  (S=1)

57
symmetry c1

| | | | |
|---|---|---|---|
| C | -1.927100000 | 2.403400000 | 0.331800000 |
| C | -2.915000000 | 3.043800000 | 1.072800000 |
| C | -3.438000000 | 2.418700000 | 2.202200000 |
| C | -2.962700000 | 1.158500000 | 2.570300000 |
| C | -1.982300000 | 0.576400000 | 1.788500000 |
| N | -1.477300000 | 1.191200000 | 0.689100000 |
| C | -1.355600000 | -0.778900000 | 2.020900000 |
| C | -1.856300000 | -1.691400000 | 0.923400000 |
| C | -2.708800000 | -2.769000000 | 1.074300000 |
| C | -3.047600000 | -3.500000000 | -0.068600000 |
| C | -2.518700000 | -3.132900000 | -1.303500000 |
| C | -1.655900000 | -2.040000000 | -1.395600000 |
| N | -1.360300000 | -1.343600000 | -0.289300000 |
| N | 0.119200000 | -0.585000000 | 1.735700000 |
| C | 0.909400000 | -1.854700000 | 1.713800000 |
| C | 0.765500000 | 0.476900000 | 2.566600000 |
| C | 1.699900000 | 1.270900000 | 1.702200000 |
| C | 1.754300000 | -1.922500000 | 0.473000000 |
| C | 2.849800000 | -2.767000000 | 0.355600000 |
| C | 3.503200000 | -2.859000000 | -0.872200000 |
| C | 3.026700000 | -2.121200000 | -1.953600000 |
| C | 1.935300000 | -1.279700000 | -1.775900000 |
| N | 1.334100000 | -1.175400000 | -0.576200000 |
| N | 1.365000000 | 1.332500000 | 0.389500000 |
| C | 2.129900000 | 2.036800000 | -0.466100000 |
| C | 3.249800000 | 2.732300000 | -0.032800000 |
| C | 3.600600000 | 2.680300000 | 1.314700000 |
| C | 2.816100000 | 1.934700000 | 2.192100000 |
| Fe | -0.071900000 | 0.119400000 | -0.173200000 |
| O | -0.262600000 | 0.742400000 | -1.725200000 |
| O | -0.443700000 | -0.895500000 | -3.863900000 |
| H | -1.484500000 | 2.843500000 | -0.559800000 |
| H | -3.262200000 | 4.027800000 | 0.759600000 |
| H | -4.205000000 | 2.910200000 | 2.800400000 |
| H | -3.334200000 | 0.640900000 | 3.453600000 |
| H | -3.084200000 | -3.038400000 | 2.060500000 |
| H | -3.710300000 | -4.362000000 | 0.014400000 |
| H | -2.763200000 | -3.693900000 | -2.205800000 |
| H | -1.190700000 | -1.681800000 | -2.348500000 |
| H | 1.513500000 | -1.950000000 | 2.624900000 |
| H | 0.209500000 | -2.703700000 | 1.691100000 |
| H | -0.015100000 | 1.152500000 | 2.945800000 |
| H | 1.276000000 | 0.040200000 | 3.434800000 |
| H | 3.177200000 | -3.347800000 | 1.217400000 |
| H | 4.371300000 | -3.509300000 | -0.983100000 |
| H | 3.496400000 | -2.185600000 | -2.934900000 |
| H | 1.470900000 | -0.717600000 | -2.588900000 |
| H | 1.823700000 | 2.015300000 | -1.509400000 |
| H | 3.842900000 | 3.293200000 | -0.754200000 |
| H | 4.484800000 | 3.204100000 | 1.678000000 |
| H | 3.066000000 | 1.858000000 | 3.249700000 |
| H | -0.464200000 | -0.004900000 | -3.453100000 |
| H | -1.545800000 | -1.172600000 | 3.028400000 |
| O | -0.670000000 | 2.820600000 | -4.042900000 |
| O | -0.261800000 | 3.130900000 | -2.647400000 |
| H | -0.243500000 | 2.176100000 | -2.263000000 |
| H | -1.654800000 | 2.880000000 | -3.960900000 |

### {[(N4Py)$Fe^{II}$(OOH)(**HOOH**)$]^+$} (S=2)

57
symmetry c1

| | | | |
|---|---|---|---|
| H | 1.740378000 | 1.035780000 | -2.989019000 |
| O | 2.238195000 | 1.921330000 | -3.203220000 |
| O | 1.210586000 | 2.867430000 | -2.676780000 |
| H | 1.479142000 | 2.930136000 | -1.724719000 |
| C | -1.667434000 | 2.756225000 | -0.711477000 |
| C | -2.707015000 | 3.593959000 | -0.321061000 |
| C | -3.569819000 | 3.172622000 | 0.690065000 |
| C | -3.364807000 | 1.922561000 | 1.273764000 |
| C | -2.307916000 | 1.144613000 | 0.823284000 |
| N | -1.466275000 | 1.547920000 | -0.157928000 |
| C | -2.032279000 | -0.252992000 | 1.349053000 |
| C | -2.524320000 | -1.206094000 | 0.273486000 |
| C | -3.686055000 | -1.956628000 | 0.379760000 |
| C | -4.063731000 | -2.753239000 | -0.702819000 |
| C | -3.264720000 | -2.766269000 | -1.846636000 |
| C | -2.111753000 | -1.988876000 | -1.873436000 |
| N | -1.747790000 | -1.220504000 | -0.832559000 |
| N | -0.559275000 | -0.408966000 | 1.462615000 |
| C | -0.114289000 | -1.778951000 | 1.799999000 |
| C | 0.089731000 | 0.611050000 | 2.315952000 |
| C | 1.477365000 | 0.896657000 | 1.801159000 |
| C | 1.203590000 | -2.072947000 | 1.125510000 |
| C | 2.257435000 | -2.705590000 | 1.773782000 |
| C | 3.434722000 | -2.955696000 | 1.067131000 |
| C | 3.509956000 | -2.578232000 | -0.272571000 |
| C | 2.416056000 | -1.940855000 | -0.847743000 |
| N | 1.287370000 | -1.679519000 | -0.165095000 |
| N | 1.595900000 | 1.009974000 | 0.455644000 |
| C | 2.822090000 | 1.244901000 | -0.052009000 |
| C | 3.957502000 | 1.380745000 | 0.738775000 |
| C | 3.830863000 | 1.259940000 | 2.120427000 |
| C | 2.567250000 | 1.014599000 | 2.655769000 |
| Fe | -0.011847000 | 0.013044000 | -0.719251000 |
| O | 0.672507000 | -0.012970000 | -2.543031000 |
| O | -0.531901000 | 0.702829000 | -3.104643000 |
| H | -0.967521000 | 3.055841000 | -1.489665000 |
| H | -2.827489000 | 4.565532000 | -0.799997000 |
| H | -4.387714000 | 3.810699000 | 1.025812000 |
| H | -4.009631000 | 1.556569000 | 2.072841000 |
| H | -4.279070000 | -1.922074000 | 1.293454000 |
| H | -4.967676000 | -3.360374000 | -0.650954000 |
| H | -3.527252000 | -3.375817000 | -2.711188000 |
| H | -1.446367000 | -1.963238000 | -2.736743000 |
| H | -0.050250000 | -1.927237000 | 2.889798000 |
| H | -0.856357000 | -2.491359000 | 1.408509000 |
| H | -0.494366000 | 1.540955000 | 2.260592000 |
| H | 0.115201000 | 0.296660000 | 3.371673000 |
| H | 2.161357000 | -2.984311000 | 2.823428000 |
| H | 4.283338000 | -3.432212000 | 1.559647000 |
| H | 4.409105000 | -2.754717000 | -0.863296000 |
| H | 2.435208000 | -1.598590000 | -1.883527000 |
| H | 2.895012000 | 1.294339000 | -1.138891000 |
| H | 4.924330000 | 1.560942000 | 0.268868000 |
| H | 4.702379000 | 1.338880000 | 2.771106000 |
| H | 2.426218000 | 0.894204000 | 3.730235000 |
| H | -0.195862000 | 1.641294000 | -3.050470000 |
| H | -2.559154000 | -0.425673000 | 2.302242000 |

### Reactants

$OCH_3^-$

| | | | |
|---|---|---|---|
| C | -0.540670000 | -0.000001000 | -0.000001000 |
| H | -1.037404000 | 0.926877000 | -0.441525000 |
| H | -1.037396000 | -0.845815000 | -0.581926000 |
| H | -1.037401000 | -0.081061000 | 1.023453000 |
| O | 0.794528000 | 0.000001000 | 0.000000000 |

$CH_3OH$

| | | | |
|---|---|---|---|
| C | 0.667181000 | -0.020330000 | 0.000000000 |
| H | 1.083462000 | 0.987269000 | -0.000046000 |
| H | 1.029384000 | -0.544565000 | 0.892941000 |
| H | 1.029378000 | -0.544643000 | -0.892898000 |
| O | -0.749497000 | 0.122014000 | 0.000000000 |
| H | -1.149333000 | -0.752194000 | 0.000001000 |

$CH_3CN$

| | | | |
|---|---|---|---|
| C | 0.000003000 | -1.176046000 | 0.000000000 |
| C | 0.000000000 | 0.280459000 | 0.000000000 |
| N | -0.000005000 | 1.433101000 | 0.000000000 |
| H | -1.024784000 | -1.552730000 | 0.000000000 |
| H | 0.512400000 | -1.552729000 | 0.887491000 |
| H | 0.512400000 | -1.552729000 | -0.887491000 |

$H_2O_2$

| | | | |
|---|---|---|---|
| O | 0.717114000 | -0.119554000 | -0.052056000 |
| O | -0.717114000 | 0.119554000 | -0.052056000 |
| H | 1.026804000 | 0.667969000 | 0.416449000 |
| H | -1.026805000 | -0.667968000 | 0.416449000 |

$OH^-$

| | | | |
|---|---|---|---|
| O | 0.000000000 | 0.000000000 | 0.107349000 |
| H | 0.000000000 | 0.000000000 | -0.858789000 |

OH•

| | | | |
|---|---|---|---|
| O | 0.000000000 | 0.000000000 | 0.108429000 |
| H | 0.000000000 | 0.000000000 | -0.867435000 |

$H_3O^+$

| | | | |
|---|---|---|---|
| O | 0.000000000 | -0.000001000 | -0.069018000 |
| H | 0.822050000 | -0.470228000 | 0.184047000 |
| H | -0.818256000 | -0.476798000 | 0.184047000 |
| H | -0.003794000 | 0.947032000 | 0.184046000 |

$O_2$

| | | | |
|---|---|---|---|
| O | 0.000000000 | 0.000000000 | 0.602881000 |
| O | 0.000000000 | 0.000000000 | -0.602881000 |

$H_2O$

| | | | |
|---|---|---|---|
| O | 0.000000000 | 0.000000000 | 0.117047000 |
| H | 0.000000000 | 0.763573000 | -0.468188000 |
| H | 0.000000000 | -0.763573000 | -0.468188000 |

**Coordinates for structures in Figure 7**

Reactant **2a**($H_2O_2$) {[(N4Py)FeII(MeOH)](HOOH)}$^{2+}$

Product H**2b**$^+$(MeOH) {[(N4Py)FeII(HOOH)](MeOH)}$^{2+}$

| Singlet | reactant | | | TS | | | Product | | |
|---|---|---|---|---|---|---|---|---|---|
| C | 1.995414 | -2.31957 | -1.0496 | 1.836939 | 2.179848 | 1.294556 | 1.541887 | -2.08394 | -1.55153 |
| C | 3.027035 | -3.20983 | -0.77821 | 2.871187 | 3.083479 | 1.083185 | 2.595517 | -2.98095 | -1.41964 |
| C | 3.599458 | -3.22559 | 0.492797 | 3.486976 | 3.141391 | -0.16559 | 3.297783 | -3.0379 | -0.21779 |
| C | 3.109865 | -2.3575 | 1.471425 | 3.031408 | 2.308474 | -1.18955 | 2.928535 | -2.19155 | 0.829738 |
| C | 2.083585 | -1.49342 | 1.133666 | 1.995934 | 1.43469 | -0.90995 | 1.8758 | -1.31693 | 0.630728 |
| N | 1.543576 | -1.45409 | -0.11974 | 1.423027 | 1.346302 | 0.322703 | 1.189152 | -1.25519 | -0.54862 |
| C | 1.450911 | -0.48233 | 2.066965 | 1.38397 | 0.456153 | -1.88826 | 1.355799 | -0.33312 | 1.658122 |
| C | 2.01535 | 0.879724 | 1.704886 | 1.86613 | -0.92102 | -1.48145 | 1.839218 | 1.049461 | 1.265355 |
| C | 2.922685 | 1.571621 | 2.49311 | 2.840444 | -1.64158 | -2.14939 | 2.807459 | 1.751034 | 1.967148 |
| C | 3.3785 | 2.811204 | 2.039562 | 3.21712 | -2.88374 | -1.63512 | 3.189556 | 3.005615 | 1.488332 |
| C | 2.898468 | 3.316267 | 0.833125 | 2.585949 | -3.36208 | -0.48946 | 2.585929 | 3.510399 | 0.339477 |
| C | 1.98291 | 2.56344 | 0.100769 | 1.606903 | -2.58922 | 0.124735 | 1.612271 | 2.751567 | -0.30495 |
| N | 1.568725 | 1.36226 | 0.522615 | 1.264561 | -1.3753 | -0.34499 | 1.254832 | 1.54166 | 0.147432 |
| N | -0.03824 | -0.46918 | 1.7032 | -0.12076 | 0.470323 | -1.58548 | -0.16579 | -0.34507 | 1.490278 |
| C | -0.79344 | 0.663867 | 2.348994 | -0.86838 | -0.60081 | -2.34139 | -0.85109 | 0.75825 | 2.25968 |
| C | -0.69926 | -1.80585 | 1.955483 | -0.75091 | 1.834236 | -1.75971 | -0.75107 | -1.70348 | 1.802727 |
| C | -1.56672 | -2.18776 | 0.783865 | -1.66594 | 2.138701 | -0.60068 | -1.79115 | -2.06148 | 0.778091 |
| C | -1.79995 | 1.214892 | 1.376127 | -1.96388 | -1.14844 | -1.47056 | -1.97006 | 1.331068 | 1.43514 |
| C | -2.9365 | 1.902235 | 1.779602 | -3.13415 | -1.6896 | -1.98563 | -3.04483 | 2.00902 | 1.992352 |
| C | -3.7982 | 2.421314 | 0.814418 | -4.10106 | -2.18295 | -1.11179 | -4.02156 | 2.546618 | 1.155919 |
| C | -3.50915 | 2.206076 | -0.53206 | -3.88247 | -2.0798 | 0.260415 | -3.90905 | 2.363371 | -0.22086 |
| C | -2.36627 | 1.495659 | -0.86977 | -2.69973 | -1.51066 | 0.711516 | -2.82209 | 1.657668 | -0.7148 |
| N | -1.50277 | 1.028003 | 0.05764 | -1.72956 | -1.08675 | -0.13001 | -1.84762 | 1.171274 | 0.085764 |
| N | -1.23423 | -1.60149 | -0.404 | -1.38756 | 1.477557 | 0.559302 | -1.65165 | -1.44728 | -0.43406 |
| C | -1.89803 | -1.9566 | -1.5262 | -2.11852 | 1.743911 | 1.661999 | -2.50402 | -1.76987 | -1.43061 |
| C | -2.92009 | -2.89582 | -1.50532 | -3.15302 | 2.669332 | 1.653469 | -3.51379 | -2.70695 | -1.2657 |
| C | -3.27982 | -3.4797 | -0.29188 | -3.45429 | 3.332144 | 0.465379 | -3.66634 | -3.32481 | -0.02646 |
| C | -2.59169 | -3.11826 | 0.86659 | -2.69903 | 3.061229 | -0.67576 | -2.79191 | -2.99322 | 1.007413 |
| H | 1.608005 | -0.72764 | 3.127163 | 1.596245 | 0.697125 | -2.93882 | 1.64954 | -0.59813 | 2.683831 |
| Fe | 0.03687 | -0.17301 | -0.26572 | -0.10096 | 0.013799 | 0.3448 | -0.3642 | -0.00299 | -0.45323 |
| O | 0.456462 | 0.121008 | -2.55625 | 1.877137 | -0.35569 | 2.761406 | 2.852025 | 0.82321 | -2.49895 |
| H | 1.505006 | -2.26761 | -2.02081 | 1.337531 | 2.075597 | 2.255035 | 0.959529 | -2.00038 | -2.46792 |
| H | 3.372639 | -3.88379 | -1.56159 | 3.190459 | 3.730517 | 1.899656 | 2.856395 | -3.62622 | -2.25778 |
| H | 4.409364 | -3.91641 | 0.727414 | 4.304321 | 3.838921 | -0.34876 | 4.124907 | -3.73665 | -0.09248 |
| H | 3.512778 | -2.35623 | 2.4839 | 3.465941 | 2.34041 | -2.18824 | 3.446419 | -2.21233 | 1.788091 |
| H | 3.256704 | 1.156385 | 3.443531 | 3.29146 | -1.2394 | -3.05622 | 3.250242 | 1.326667 | 2.867989 |
| H | 4.092663 | 3.382494 | 2.633309 | 3.985453 | -3.47454 | -2.13396 | 3.951864 | 3.582908 | 2.011993 |
| H | 3.223168 | 4.286477 | 0.457576 | 2.842666 | -4.3329 | -0.06663 | 2.862527 | 4.484111 | -0.06353 |
| H | 1.557905 | 2.914785 | -0.83752 | 1.072886 | -2.93431 | 1.007741 | 1.122191 | 3.093521 | -1.21409 |

| | | | | | | | | | |
|---|---|---|---|---|---|---|---|---|---|
| H | -1.26257 | 0.33568 | 3.285787 | -1.2537 | -0.20332 | -3.28926 | -1.20526 | 0.387818 | 3.230243 |
| H | -0.07596 | 1.460186 | 2.593519 | -0.1626 | -1.41063 | -2.57471 | -0.11595 | 1.552802 | 2.451398 |
| H | 0.092311 | -2.56341 | 2.054302 | 0.05511 | 2.582981 | -1.76911 | 0.059435 | -2.44472 | 1.740051 |
| H | -1.26464 | -1.78615 | 2.895673 | -1.27596 | 1.891358 | -2.72143 | -1.15035 | -1.72671 | 2.824483 |
| H | -3.14172 | 2.025737 | 2.842887 | -3.28397 | -1.71653 | -3.06467 | -3.11309 | 2.109519 | 3.075241 |
| H | -4.69295 | 2.968177 | 1.111203 | -5.02305 | -2.6178 | -1.49773 | -4.87023 | 3.085807 | 1.576526 |
| H | -4.16697 | 2.570071 | -1.32086 | -4.62366 | -2.41888 | 0.98357 | -4.66041 | 2.745077 | -0.911 |
| H | -2.12264 | 1.274226 | -1.90496 | -2.50509 | -1.35343 | 1.771402 | -2.72568 | 1.440446 | -1.77832 |
| H | -1.57593 | -1.45941 | -2.44012 | -1.85314 | 1.180518 | 2.556293 | -2.36393 | -1.24899 | -2.37494 |
| H | -3.42815 | -3.15473 | -2.43363 | -3.71814 | 2.851744 | 2.566941 | -4.17463 | -2.93296 | -2.10164 |
| H | -4.08939 | -4.20835 | -0.24502 | -4.27347 | 4.050153 | 0.4236 | -4.46035 | -4.05313 | 0.137126 |
| H | -2.84254 | -3.55779 | 1.831728 | -2.90799 | 3.560895 | -1.62133 | -2.88096 | -3.45374 | 1.991178 |
| C | 1.828942 | 0.554145 | -2.80579 | 3.193104 | -0.54645 | 2.202212 | 3.92165 | 0.522648 | -1.58073 |
| H | 1.884552 | 1.649763 | -2.82595 | 3.629376 | -1.50749 | 2.513525 | 3.851669 | 1.115455 | -0.65589 |
| H | 2.459834 | 0.181807 | -1.99209 | 3.179047 | -0.49633 | 1.101651 | 3.950792 | -0.54645 | -1.32099 |
| H | 2.168139 | 0.142574 | -3.76633 | 3.82231 | 0.271541 | 2.575993 | 4.858178 | 0.782971 | -2.08938 |
| H | -0.10714 | 0.858116 | -2.92563 | 1.365179 | -1.19335 | 2.68049 | 2.008923 | 0.625905 | -2.04087 |
| O | -0.78636 | 2.366262 | -3.45798 | -0.42597 | -1.84654 | 2.696315 | -0.27528 | 0.499859 | -2.72197 |
| O | -0.1836 | 3.234229 | -2.42014 | -0.65486 | -3.2614 | 2.336114 | -0.25351 | 1.971467 | -2.96282 |
| H | -0.37761 | 2.749641 | -4.27778 | -0.73636 | -1.84516 | 3.638684 | -0.99396 | 0.212924 | -3.34135 |
| H | -0.98688 | 3.74218 | -2.13826 | -1.42389 | -3.16125 | 1.715247 | -0.92181 | 2.281803 | -2.29919 |

| Triplet | reactant | | | TS | | | Product | | |
|---|---|---|---|---|---|---|---|---|---|
| C | 2.107093 | 2.207361 | 1.091245 | 1.904304 | -2.18335 | -1.08611 | 1.421012 | 2.150163 | 1.418238 |
| C | 3.109045 | 3.134381 | 0.833904 | 2.96826 | -3.03264 | -0.81313 | 2.526814 | 2.990383 | 1.360746 |
| C | 3.609042 | 3.251756 | -0.46238 | 3.590434 | -2.97035 | 0.432544 | 3.297023 | 3.026466 | 0.201292 |
| C | 3.087802 | 2.44127 | -1.47205 | 3.112752 | -2.07433 | 1.389872 | 2.934978 | 2.23888 | -0.89421 |
| C | 2.095688 | 1.531109 | -1.14422 | 2.047656 | -1.25637 | 1.052606 | 1.821099 | 1.428797 | -0.77467 |
| N | 1.625628 | 1.40848 | 0.122248 | 1.475806 | -1.288 | -0.1775 | 1.100513 | 1.375263 | 0.371803 |
| C | 1.435331 | 0.581719 | -2.12557 | 1.413674 | -0.23225 | 1.97142 | 1.271102 | 0.496569 | -1.83104 |
| C | 1.861805 | -0.82196 | -1.71776 | 1.782931 | 1.123311 | 1.398635 | 1.590952 | -0.90666 | -1.36948 |
| C | 2.691647 | -1.63141 | -2.47678 | 2.687148 | 1.992674 | 1.983765 | 2.548844 | -1.74727 | -1.90374 |
| C | 2.990574 | -2.91346 | -2.01314 | 2.952989 | 3.208024 | 1.351757 | 2.732557 | -2.99732 | -1.30631 |
| C | 2.434218 | -3.34722 | -0.81061 | 2.28493 | 3.514631 | 0.168069 | 1.939738 | -3.36798 | -0.22337 |
| C | 1.611968 | -2.48742 | -0.09416 | 1.388259 | 2.598128 | -0.36654 | 0.981257 | -2.48199 | 0.254684 |
| N | 1.346652 | -1.23737 | -0.52395 | 1.158361 | 1.406885 | 0.220689 | 0.841291 | -1.26925 | -0.29933 |
| N | -0.04216 | 0.672987 | -1.91722 | -0.07254 | -0.37513 | 1.822196 | -0.23642 | 0.619935 | -1.72884 |
| C | -0.85821 | -0.36566 | -2.55728 | -0.88615 | 0.714288 | 2.399766 | -0.98843 | -0.39359 | -2.53429 |
| C | -0.65809 | 2.008346 | -1.85977 | -0.63771 | -1.72998 | 2.007184 | -0.74841 | 2.012941 | -1.92451 |
| C | -1.48855 | 2.224212 | -0.60195 | -1.50616 | -2.14997 | 0.834574 | -1.76402 | 2.334531 | -0.86538 |
| C | -1.79803 | -1.0409 | -1.57675 | -1.95547 | 1.159236 | 1.427098 | -2.0886 | -0.99134 | -1.70363 |
| C | -2.95142 | -1.68474 | -2.01347 | -3.15202 | 1.718777 | 1.85994 | -3.20438 | -1.60862 | -2.25237 |
| C | -3.76927 | -2.3345 | -1.0933 | -4.09733 | 2.132727 | 0.92475 | -4.13116 | -2.21146 | -1.40523 |
| C | -3.42129 | -2.30678 | 0.256655 | -3.83023 | 1.947307 | -0.43037 | -3.91984 | -2.18109 | -0.02815 |
| C | -2.26719 | -1.63708 | 0.633376 | -2.62634 | 1.368242 | -0.80166 | -2.79703 | -1.53425 | 0.466552 |
| N | -1.46131 | -1.02994 | -0.26334 | -1.69093 | 1.005946 | 0.103334 | -1.90577 | -0.95795 | -0.36118 |

| | | | | | | | | | |
|---|---|---|---|---|---|---|---|---|---|
| N | -1.16026 | 1.524154 | 0.519543 | -1.27438 | -1.56319 | -0.37062 | -1.66388 | 1.63033 | 0.288227 |
| C | -1.8224 | 1.762389 | 1.670871 | -1.973 | -1.95905 | -1.4543 | -2.48771 | 1.88839 | 1.321178 |
| C | -2.84901 | 2.692381 | 1.754576 | -2.93786 | -2.95361 | -1.38289 | -3.45191 | 2.882078 | 1.237357 |
| C | -3.21045 | 3.396044 | 0.607737 | -3.19793 | -3.54779 | -0.14965 | -3.57344 | 3.609844 | 0.055299 |
| C | -2.51911 | 3.157306 | -0.57883 | -2.47349 | -3.1383 | 0.968114 | -2.71942 | 3.331161 | -1.00925 |
| H | 1.74598 | 0.802292 | -3.15948 | 1.763657 | -0.34381 | 3.008747 | 1.648814 | 0.716031 | -2.83838 |
| Fe | 0.125822 | 0.065797 | 0.28491 | -0.06296 | -0.04215 | -0.31853 | -0.4128 | 0.133888 | 0.254013 |
| O | 0.668243 | -0.26946 | 2.512166 | 1.823698 | 0.150357 | -2.84525 | 2.030564 | -1.03178 | 2.815561 |
| H | 1.665174 | 2.077426 | 2.078116 | 1.395713 | -2.171 | -2.04677 | 0.785044 | 2.058537 | 2.297176 |
| H | 3.484099 | 3.75893 | 1.643885 | 3.302935 | -3.73311 | -1.57771 | 2.778942 | 3.601102 | 2.226541 |
| H | 4.388444 | 3.97888 | -0.69061 | 4.429331 | -3.62635 | 0.66553 | 4.171611 | 3.673897 | 0.141157 |
| H | 3.434973 | 2.521302 | -2.50164 | 3.550905 | -2.01529 | 2.385649 | 3.498791 | 2.259826 | -1.82554 |
| H | 3.080829 | -1.26755 | -3.42751 | 3.164897 | 1.724379 | 2.92551 | 3.135928 | -1.43129 | -2.76472 |
| H | 3.635158 | -3.57215 | -2.59563 | 3.660255 | 3.912264 | 1.789649 | 3.488217 | -3.67986 | -1.69531 |
| H | 2.628924 | -4.34748 | -0.42518 | 2.451709 | 4.46012 | -0.34741 | 2.058313 | -4.33236 | 0.267488 |
| H | 1.136775 | -2.77608 | 0.842196 | 0.837587 | 2.804926 | -1.28234 | 0.334694 | -2.70922 | 1.099281 |
| H | -1.43263 | 0.043112 | -3.40346 | -1.33573 | 0.413829 | 3.35768 | -1.3729 | 0.053741 | -3.45962 |
| H | -0.19455 | -1.14053 | -2.97106 | -0.23236 | 1.575368 | 2.605221 | -0.29634 | -1.19938 | -2.8215 |
| H | 0.136705 | 2.770648 | -1.86154 | 0.188119 | -2.45332 | 2.088167 | 0.092066 | 2.715895 | -1.82383 |
| H | -1.27739 | 2.196708 | -2.75066 | -1.20996 | -1.79244 | 2.944237 | -1.15984 | 2.136921 | -2.93405 |
| H | -3.20253 | -1.66902 | -3.07448 | -3.33869 | 1.8191 | 2.929089 | -3.338 | -1.61471 | -3.33354 |
| H | -4.67566 | -2.84218 | -1.42331 | -5.03921 | 2.573521 | 1.251065 | -5.01469 | -2.69824 | -1.81852 |
| H | -4.04044 | -2.783 | 1.015944 | -4.55092 | 2.225395 | -1.19852 | -4.62125 | -2.64169 | 0.665937 |
| H | -1.97046 | -1.55612 | 1.675769 | -2.38372 | 1.154916 | -1.84096 | -2.57806 | -1.45371 | 1.529661 |
| H | -1.49943 | 1.177304 | 2.530457 | -1.74082 | -1.44448 | -2.38593 | -2.34649 | 1.271223 | 2.206593 |
| H | -3.35943 | 2.847138 | 2.704618 | -3.48148 | -3.24175 | -2.2815 | -4.10291 | 3.068602 | 2.090395 |
| H | -4.02456 | 4.120883 | 0.633517 | -3.96155 | -4.31996 | -0.0561 | -4.3323 | 4.386296 | -0.04077 |
| H | -2.77621 | 3.693045 | -1.49273 | -2.65428 | -3.58222 | 1.947051 | -2.78641 | 3.880025 | -1.9479 |
| C | 2.02846 | -0.72153 | 2.76502 | 3.113777 | 0.48949 | -2.29738 | 3.163174 | -0.59514 | 2.037179 |
| H | 2.052213 | -1.80747 | 2.925974 | 3.490894 | 1.440139 | -2.70465 | 4.009218 | -1.23437 | 2.318212 |
| H | 2.631773 | -0.47707 | 1.882254 | 3.086284 | 0.554595 | -1.19785 | 2.991418 | -0.7012 | 0.956288 |
| H | 2.432048 | -0.20391 | 3.646519 | 3.803244 | -0.31811 | -2.57621 | 3.418845 | 0.45178 | 2.258329 |
| H | 0.063764 | -0.9136 | 2.977354 | 1.246631 | 0.949882 | -2.83719 | 1.932806 | -2.76077 | 2.915348 |
| O | -0.84973 | -2.2678 | 3.54217 | -0.49213 | 1.666472 | -3.00792 | -0.45273 | -0.26051 | 1.867759 |
| O | -0.22012 | -3.29917 | 2.685681 | -0.65855 | 3.128787 | -2.84763 | 1.878483 | -3.75849 | 2.952895 |
| H | -0.557 | -2.5688 | 4.442944 | -0.7399 | 1.557943 | -3.96308 | 1.185025 | -0.6484 | 2.449336 |
| H | -1.01268 | -3.85792 | 2.476902 | -1.51353 | 3.155239 | -2.34432 | 1.803349 | -3.94677 | 3.913475 |

| Quintet | reactant | | | TS | | | Product | | |
|---|---|---|---|---|---|---|---|---|---|
| Fe | -0.45669 | -0.03868 | -1.00379 | -0.07385 | 0.005736 | 0.709663 | -0.23147 | 0.050594 | 0.40891 |
| O | -1.37114 | 0.158607 | -2.61607 | -1.47706 | 0.264442 | 2.408379 | -0.57488 | 0.405009 | 2.378643 |
| H | -3.98145 | 3.902887 | 1.636216 | -4.62622 | -3.64442 | -0.77587 | -4.6244 | -3.77746 | -1.10709 |
| C | -2.04387 | 1.178421 | 1.130349 | -2.1406 | -1.35467 | -0.96179 | -2.27665 | -1.34252 | -1.22662 |
| N | -1.62629 | 1.42083 | -0.13308 | -1.59394 | -1.47744 | 0.266429 | -1.80901 | -1.38422 | 0.041527 |

| | | | | | | | | | |
|---|---|---|---|---|---|---|---|---|---|
| C | -1.46241 | -0.08161 | 1.73401 | -1.48223 | -0.29442 | -1.82399 | -1.6225 | -0.27619 | -2.08798 |
| C | -2.17058 | -1.27103 | 1.118041 | -1.97528 | 1.057547 | -1.33669 | -2.17177 | 1.053912 | -1.60244 |
| C | -3.04418 | -2.10647 | 1.795419 | -2.95684 | 1.797834 | -1.98012 | -3.11709 | 1.796478 | -2.29449 |
| C | -3.63623 | -3.15168 | 1.083081 | -3.33238 | 3.021939 | -1.42553 | -3.57282 | 2.985937 | -1.72435 |
| C | -3.32607 | -3.32835 | -0.26379 | -2.70331 | 3.465345 | -0.26261 | -3.06023 | 3.39003 | -0.49222 |
| C | -2.43354 | -2.45078 | -0.8727 | -1.72883 | 2.6631 | 0.319573 | -2.11085 | 2.591467 | 0.135841 |
| N | -1.88654 | -1.44015 | -0.1849 | -1.38513 | 1.473529 | -0.19783 | -1.68252 | 1.438494 | -0.4028 |
| N | -0.03039 | -0.12834 | 1.299997 | -0.02062 | -0.34478 | -1.56037 | -0.16085 | -0.29609 | -1.79155 |
| C | 0.652156 | -1.37162 | 1.778292 | 0.741332 | 0.718496 | -2.2503 | 0.607119 | 0.818394 | -2.39957 |
| C | 0.724824 | 1.100196 | 1.66464 | 0.594854 | -1.66685 | -1.79398 | 0.492727 | -1.60927 | -2.01699 |
| C | 1.668339 | 1.459178 | 0.54742 | 1.75827 | -1.86654 | -0.86091 | 1.59119 | -1.81753 | -1.00365 |
| C | 2.126882 | -1.32472 | 1.490316 | 1.890809 | 1.198814 | -1.40448 | 1.723832 | 1.243864 | -1.47737 |
| C | 3.090227 | -1.15448 | 2.472804 | 3.138543 | 1.468777 | -1.9593 | 2.992712 | 1.566263 | -1.94051 |
| C | 4.428444 | -1.00794 | 2.105524 | 4.13025 | 2.033961 | -1.161 | 3.96995 | 1.964697 | -1.02752 |
| C | 4.784977 | -1.03832 | 0.759889 | 3.82139 | 2.356465 | 0.158908 | 3.639283 | 2.041417 | 0.323583 |
| C | 3.794886 | -1.23918 | -0.18805 | 2.55506 | 2.041779 | 0.638849 | 2.348941 | 1.703124 | 0.713494 |
| N | 2.518027 | -1.3829 | 0.196744 | 1.612382 | 1.42837 | -0.10174 | 1.407994 | 1.302364 | -0.1611 |
| N | 1.230475 | 1.219195 | -0.70623 | 1.570569 | -1.47581 | 0.423372 | 1.296273 | -1.4381 | 0.263201 |
| C | 2.021681 | 1.49457 | -1.75885 | 2.545046 | -1.75811 | 1.312086 | 2.218994 | -1.61583 | 1.226664 |
| C | 3.293608 | 2.026269 | -1.59602 | 3.742032 | -2.37085 | 0.961166 | 3.471149 | -2.16192 | 0.968673 |
| C | 3.759535 | 2.267141 | -0.30468 | 3.951738 | -2.71657 | -0.37116 | 3.781615 | -2.53784 | -0.33637 |
| C | 2.93411 | 1.984434 | 0.780583 | 2.934269 | -2.47202 | -1.29007 | 2.822376 | -2.367 | -1.33529 |
| H | -1.56451 | -0.07976 | 2.830759 | -1.72994 | -0.4438 | -2.88783 | -1.84571 | -0.43447 | -3.15504 |
| C | -2.90072 | 2.040887 | 1.793422 | -3.22906 | -2.10813 | -1.37451 | -3.28576 | -2.17993 | -1.67762 |
| H | -3.25119 | -1.94787 | 2.853317 | -3.41051 | 1.428075 | -2.89967 | -3.48219 | 1.454679 | -3.26292 |
| H | -4.32795 | -3.8297 | 1.583264 | -4.09893 | 3.630175 | -1.90623 | -4.31271 | 3.595833 | -2.24312 |
| H | -3.76498 | -4.13975 | -0.84324 | -2.95884 | 4.423867 | 0.188124 | -3.38451 | 4.31765 | -0.02164 |
| H | -2.13433 | -2.53951 | -1.91837 | -1.20377 | 2.958608 | 1.228008 | -1.66659 | 2.868929 | 1.091424 |
| H | 0.504415 | -1.48626 | 2.86387 | 1.086759 | 0.380239 | -3.23965 | 0.993698 | 0.540473 | -3.39204 |
| H | 0.196062 | -2.23145 | 1.267443 | 0.07878 | 1.582059 | -2.41232 | -0.06724 | 1.677167 | -2.53408 |
| H | 0.02132 | 1.933072 | 1.801802 | -0.14314 | -2.45187 | -1.57487 | -0.25551 | -2.40217 | -1.87264 |
| H | 1.258348 | 0.980093 | 2.618879 | 0.900889 | -1.78596 | -2.84499 | 0.874307 | -1.69433 | -3.04589 |
| H | 2.789485 | -1.11229 | 3.518357 | 3.326642 | 1.235628 | -3.00749 | 3.216697 | 1.49151 | -3.00474 |
| H | 5.187977 | -0.85341 | 2.871563 | 5.121865 | 2.233909 | -1.56727 | 4.977377 | 2.204789 | -1.36853 |
| H | 5.817402 | -0.90739 | 0.44115 | 4.545429 | 2.839899 | 0.81383 | 4.369913 | 2.347739 | 1.072082 |
| H | 3.981944 | -1.27666 | -1.25899 | 2.272441 | 2.326269 | 1.655321 | 2.051234 | 1.729767 | 1.760214 |
| H | 1.617533 | 1.249197 | -2.74008 | 2.350104 | -1.48542 | 2.3483 | 1.940975 | -1.27819 | 2.225092 |
| H | 3.911798 | 2.224253 | -2.47088 | 4.49501 | -2.56015 | 1.725427 | 4.190635 | -2.27549 | 1.779275 |
| H | 4.765503 | 2.65449 | -0.14248 | 4.888992 | -3.17329 | -0.68989 | 4.761189 | -2.95102 | -0.57823 |
| H | 3.27326 | 2.141766 | 1.803939 | 3.047741 | -2.74501 | -2.33926 | 3.031775 | -2.64427 | -2.3687 |
| C | -2.78761 | 0.312214 | -2.72758 | -2.90735 | 0.499393 | 2.327872 | -1.92826 | 0.551171 | 2.90611 |
| H | -3.19291 | -0.51575 | -3.3321 | -3.13385 | 1.562733 | 2.476839 | -2.07346 | 1.573896 | 3.274793 |
| H | -3.28861 | 0.304424 | -1.7473 | -3.23152 | 0.201044 | 1.324994 | -2.61813 | 0.355437 | 2.078372 |
| H | -3.01589 | 1.263507 | -3.23454 | -3.42176 | -0.11064 | 3.082193 | -2.08489 | -0.17746 | 3.71115 |
| H | -0.9444 | -1.21093 | -3.60597 | -1.07942 | 0.84491 | 3.105781 | 0.084966 | 0.804536 | 3.034375 |
| C | -1.99138 | 2.554026 | -0.7547 | -2.10488 | -2.38522 | 1.115889 | -2.32664 | -2.28282 | 0.895515 |

| | | | | | | | | | |
|---|---|---|---|---|---|---|---|---|---|
| C | -2.83706 | 3.470627 | -0.14419 | -3.19309 | -3.18173 | 0.777149 | -3.34459 | -3.15381 | 0.521612 |
| H | -3.11693 | 4.3799 | -0.67381 | -3.58088 | -3.90339 | 1.495944 | -3.74041 | -3.86664 | 1.244603 |
| H | -3.22108 | 1.818078 | 2.810325 | -3.64171 | -1.97331 | -2.37413 | -3.62789 | -2.11854 | -2.71052 |
| C | -3.31132 | 3.20246 | 1.137845 | -3.76985 | -3.03635 | -0.48364 | -3.83301 | -3.10088 | -0.78343 |
| H | -1.59196 | 2.699191 | -1.75663 | -1.62977 | -2.44315 | 2.094991 | -1.89996 | -2.29341 | 1.898753 |
| O | -0.63592 | -2.11228 | -3.91078 | 0.640874 | 1.512904 | 3.496469 | 1.197811 | 1.442012 | 4.050659 |
| O | 0.576047 | -1.27056 | -1.49908 | 1.103506 | 0.364168 | 2.643292 | 2.196059 | 0.348894 | 3.979939 |
| H | 1.733518 | -1.44618 | -0.56596 | 1.997545 | 0.699522 | 2.377081 | 3.030408 | 0.874019 | 4.096843 |
| H | 0.019195 | -2.28938 | -3.19441 | 0.884033 | 1.159594 | 4.394756 | 0.875139 | 1.33536 | 4.985399 |
